# The effect of live attenuated influenza vaccine on pneumococcal colonisation densities among children aged 24–59 months in The Gambia: a phase 4, open label, randomised, controlled trial

**DOI:** 10.1016/S2666-5247(21)00179-8

**Published:** 2021-12

**Authors:** Chikondi Peno, Edwin P Armitage, Melanie Clerc, Carlos Balcazar Lopez, Ya Jankey Jagne, Sainabou Drammeh, Sheikh Jarju, Hadijatou Sallah, Elina Senghore, Benjamin B Lindsey, Janko Camara, Sulayman Bah, Nuredin I Mohammed, David H Dockrell, Beate Kampmann, Ed Clarke, Debby Bogaert, Thushan I de Silva

**Affiliations:** a Centre for Inflammation Research, Queen's Medical Research Institute, University of Edinburgh, Edinburgh, UK; b Vaccines and Immunity Theme, Medical Research Council Unit The Gambia at the London School of Hygiene & Tropical Medicine, Banjul, The Gambia; c The Vaccine Centre, Faculty of Infectious and Tropical Diseases, The London School of Hygiene & Tropical Medicine, London, UK; d Department of Paediatric Immunology and Infectious Diseases, Wilhelmina Children's Hospital/University Medical Center Utrecht, Utrecht, The Netherlands; e The Florey Institute & Department of Infection, Immunity and Cardiovascular Disease, The Medical School, The University of Sheffield, UK

## Abstract

**Background:**

Influenza and other respiratory viruses promote *Streptococcus pneumoniae* proliferation in the upper respiratory tract. We sought to investigate for what we believe is the first time, the effect of intranasal live attenuated influenza vaccine (LAIV) on nasopharyngeal *S pneumoniae* density in a low-income to middle-income country population with high pneumococcal carriage rates.

**Methods:**

In an open-label, randomised, controlled trial in The Gambia, 330 healthy children aged 24–59 months were randomly assigned 2:1 to receive one trivalent LAIV dose at enrolment (day 0, intervention) or at the end of active follow-up (day 21, control). The investigator team were initially masked to block size and randomisation sequence to avoid allocation bias. Group allocation was later revealed to the investigator team. The primary outcome was PCR-quantified day 7 and 21 pneumococcal density. Asymptomatic respiratory viral infection at baseline and LAIV strain shedding were included as covariates in generalised mixed-effects models, to assess the effect of LAIV and other variables on pneumococcal densities. The study is registered at ClinicalTrials.gov, NCT02972957, and is closed to recruitment.

**Findings:**

Between Feb 8 and April 12, 2017, and Jan 15 and March 28, 2018, of 343 children assessed for eligibility, 213 in the intervention group and 108 in the control group completed the study and were included in the final analysis. Although no significant differences were seen in pneumococcal carriage or density at each timepoint when comparing groups, changes from baseline were observed in the LAIV group. The baseline *S pneumoniae* carriage prevalence was high in both LAIV and control groups (75%) and increased by day 21 in the LAIV group (85%, p=0·0037), but not in the control group (79%, p=0·44). An increase in pneumococcal density from day 0 amounts was seen in the LAIV group at day 7 (+0·207 log_10_ copies per μL, SE 0·105, p=0·050) and day 21 (+0·280 log_10_ copies per μL, SE 0·105, p=0·0082), but not in the control group. Older age was associated with lower pneumococcal density (−0·015 log_10_ copies per μL, SE 0·005, p=0·0030), with the presence of asymptomatic respiratory viruses at baseline (+0·259 log_10_ copies per μL, SE 0·097, p=0·017), and greater LAIV shedding at day 7 (+0·380 log_10_ copies per μL, SE 0·167, p=0·024) associated with higher pneumococcal density. A significant increase in rhinorrhoea was reported in the LAIV group compared with the control group children during the first 7 days of the study (103 [48%] of 213, compared with 25 [23%] of 108, p<0·0001), and between day 7 and 21 (108 [51%] of 213, compared with 28 [26%] of 108, p<0·0001).

**Interpretation:**

LAIV was associated with a modest increase in nasopharyngeal pneumococcal carriage and density in the 21 days following vaccination, with the increase in density lower in magnitude than previously described in the UK. This increase was accelerated when LAIV was administered in the presence of pre-existing asymptomatic respiratory viruses, suggesting that nasopharyngeal *S pneumoniae* proliferation is driven by cumulative mixed-viral co-infections. The effect of LAIV on pneumococcal density is probably similar to other respiratory viral infections in children. Our findings provide reassurance for the use of LAIV to expand influenza vaccine programmes in low-income to middle-income country populations with high pneumococcal carriage.

**Funding:**

Wellcome Trust.

## Introduction

*Streptococcus pneumoniae* is a leading cause of respiratory tract infections (RTIs), meningitis, and sepsis globally.^1^ The highest burden of pneumococcal disease is found in children from low-income and middle-income countries (LMICs).^1^ Nasopharyngeal colonisation with *S pneumoniae* is common and usually asymptomatic. However, *S pneumoniae* colonisation represents a necessary precursor to invasive pneumococcal disease and a source of pneumococcal transmission between individuals.^2^ Higher pneumococcal densities are associated with pneumococcal disease, although pneumococcal density thresholds that result in higher risk of disease are not defined.^3^

**Research in context**

**Evidence before this study**

We searched PubMed and Web of Science for research articles published in English up to April 7, 2021 with the terms: “*Streptococcus pneumoniae*” OR “pneumococcus” AND “colonisation” OR “colonisation density” OR “bacterial load” AND “live attenuated influenza vaccine” OR “asymptomatic influenza infection” OR “asymptomatic viral infection”. This search identified five studies on the effect of live attenuated influenza vaccine (LAIV) or asymptomatic influenza−viral infection on *S pneumoniae* colonisation dynamics. The first study used a murine model to investigate the effect of LAIV on nasopharyngeal *S pneumoniae* and *Staphylococcus aureus* densities, showing increased bacterial loads for both following LAIV. The second study was a randomised, clinical trial in children aged 2–4 years from the UK, examining the effect of LAIV on upper respiratory tract colonisation with *S pneumoniae* and other bacteria. This study found a six-times increase in pneumococcal colonisation density at 28 days following one dose of LAIV in children colonised with *S pneumoniae* at baseline (increase from 2687 copies per mL at baseline to 16687 copies per mL at day 28). The third study was a longitudinal study in children aged between 48 and 96 months in the USA, which showed increased colonisation densities of *S pneumoniae* in the upper respiratory tract during symptomatic and asymptomatic respiratory viral infections. The fourth study was a cross-sectional prospective study investigating nasopharyngeal carriage of *S pneumoniae* and other bacteria among children in Greece, aged 3 months to 6 years old, presenting with and without symptomatic respiratory symptoms. This study reported that carriage prevalences were significantly higher in symptomatic compared with asymptomatic children for *S pneumoniae* (37·1% compared with 23·6%). The fifth study consisted of two randomised, control trials in adults, which used a human co-challenge model with LAIV and *S pneumoniae* in the UK. The first trial investigated pneumococcal density in adults who were randomly assigned to receive LAIV or placebo before challenge with *S pneumoniae* 3 days later. In this trial, a ten-times increase in *S pneumoniae* densities was observed by day 9 in LAIV recipients but not in the control group. In the second trial, adults were first challenged with *S pneumoniae* before receiving LAIV or placebo 3 days later. This study paradoxically observed reduced *S pneumoniae* densities in adults in the LAIV group. We did not find any published studies from low-income and middle-income countries (LMICs) or with high pneumococcal carriage prevalences, investigating the effect of LAIV or asymptomatic viral infections on *S pneumoniae* colonisation dynamics.

**Added value of this study**

To the best of our knowledge, our study is the first longitudinal, randomised, controlled trial reporting the effect of LAIV and asymptomatic viral infections on nasopharyngeal colonisation and density of *S pneumoniae* in a low-income to middle-income country population with high pneumococcal carriage prevalences. Our results show that LAIV leads to an increase in nasopharyngeal pneumococcal density in this population, similar to the effect caused by asymptomatic viral infections in this cohort. Although, to a much lower magnitude (1·78 times the original magnitude) than previously shown in the study of UK children given LAIV (six times). We also report a small increase in the proportion of children with pneumococcal carriage 21 days after receiving LAIV (75–85%). Additionally, we show that LAIV administered in the presence of pre-existing asymptomatic respiratory viruses accelerates pneumococcal proliferation in the nasopharynx. Our study is, we believe, the first to show that LAIV leads to modest increases in *S pneumoniae* colonisation and density in children residing in an area of high pneumococcal carriage, and to a degree not dissimilar to fluctuations seen due to repeated asymptomatic respiratory viral infections.

**Implications of all the available evidence**

Our study contributes to the existing knowledge highlighting the importance of asymptomatic viral infections on *S pneumoniae* colonisation densities in the nasopharynx, as well as the specific effect of LAIV on *S pneumoniae* dynamics in young children. Asymptomatic viral infections are common and could be an important contributing factor to high colonisation densities and consequently transmission rates, even after introduction of pneumococcal vaccines in LMICs. Strategies to reduce pneumococcal carriage, transmission rates, and disease should also consider interventions to reduce respiratory viral infections, especially in at risk populations including children. Our data provides evidence that the effect of LAIV on *S pneumoniae* density in children residing in an area of high pneumococcal carriage is modest and supports the wider rollout of LAIV in LMICs to help reduce the burden of influenza-related morbidity and mortality.

Viral-bacterial co-infections are commonly observed in RTIs and associated with greater disease severity. Increased risk of secondary pneumococcal pneumonia following influenza infection is well documented. Influenza virus infection has been shown to enhance acquisition, proliferation, and transmission of *S pneumoniae* in the upper respiratory tract (URT).^4^ Although the underlying mechanisms are not exhaustively defined, existing evidence suggests that influenza viruses induce mucosal epithelial disruption, leading to elevated production of both pro-inflammatory and anti-inflammatory cytokines and impaired macrophage phagocytic function.^5^ This provides a conducive environment for proliferation and invasion of *S pneumoniae*, and increases host susceptibility to severe lower RTIs and invasive disease.^5^, ^6^

Live attenuated influenza vaccine (LAIV) is administered as a nasal spray for the prevention of influenza infection and is safe and highly efficacious in young children.^7^ LAIV strains are able to replicate in the URT similar to wild-type influenza viruses; however, their cold-adapted and temperature sensitive nature prevents replication in the lower respiratory tract.^8^ LAIV has been shown to increase pneumococcal colonisation densities in murine models and in one study of children from a high-income setting, where a six-times increase from baseline was observed.^9^, ^10^ These effects are similar to wild-type influenza infection, but without progression to clinical symptoms or disease.^9^, ^11^ These data highlight the potential indirect consequences of LAIV on *S pneumoniae* density in the URT, as well as the potential importance of paucisymptomatic or asymptomatic viral infection on colonisation dynamics of residing nasopharyngeal bacteria such as *S pneumoniae*.

There are no data available on the effect of LAIV on nasopharyngeal *S pneumoniae* proliferation in children from LMICs, where in many settings high rates of pneumococcal carriage and disease are seen. Despite high uptake of pneumococcal conjugate vaccines (PCVs), pneumococcal carriage rates in The Gambia in children younger than 5 years continues to be over 70%, primarily with non-PCV serotypes. The burden of influenza in LMICs is not well known due to lack of robust surveillance data in these settings, but influenza-related mortality in sub-Saharan Africa is estimated to be higher compared with other regions.^12^ Routine influenza vaccination programmes for at-risk groups such as children younger than 5 years are not in place in most of sub-Saharan Africa. LAIV could be a good option to address this gap in LMICs, given its needle-free delivery, lower manufacturing costs and high acceptability.^13^, ^14^, ^15^ Evaluating any potential unwanted effects such as the effect on nasopharyngeal *S pneumoniae* proliferation are, therefore, vital. We describe results from a randomised, controlled trial to explore the effect of LAIV on pneumococcal colonisation densities in children aged 24–59 months in The Gambia, in addition to examining the contribution of asymptomatic respiratory viral infections to these viral–bacterial interactions.

## Methods

### Study design and participants

We did an open-label, phase 4 RCT (NCT02972957) across 2 years (2017 and 2018). Recruiment was done outside the peak influenza transmission season in The Gambia, which coincides with the rainy season during June to October each year.^16^ We have previously shown that all respiratory viral infections are lower during the dry season in The Gambia, although year round transmission of many viruses still occurs, in particular human rhinoviruses.^16^ Participants were healthy, influenza vaccine naive children aged 24–59 months from a peri-urban community setting (Sukuta), with no history of respiratory infections within the past 14 days (full inclusion and exclusion criteria in the appendix pp 2–3).

The study was approved by The Gambia Government and UK Medical Research Council joint ethics committee and the Medicines Control Agency of The Gambia. Parents provided written or thumbprinted informed consent for their children to participate. If parents were not English literate, an impartial witness was present during the informed consent discussion done in a local language, who signed to confirm completeness of the consent provided. The trial protocol is included in the appendix.

### Randomisation and masking

Children were randomly assigned to receive LAIV on the day of enrolment (day 0) or delayed until day 21 (control group). Block randomisation stratified by sex was done by use of sealed opaque envelopes and a computer-generated randomisation sequence pre-prepared by an independent statistician not involved in the rest of the study. The investigator team was masked to block size and randomisation sequence to avoid allocation bias. Group allocation was revealed on opening the envelope, cross-checked and co-signed by a second individual. As an open-label study, from this point on, the investigator team were aware of group allocation. Because of the need to carry out LAIV quantitative PCR (qPCR) on samples in the LAIV group only, group allocation was also revealed to the local laboratory team after recruitment. The external laboratory where pneumococcal qPCR was done was masked to group allocation until analysis.

### Procedures

Northern hemisphere Russian-backbone trivalent LAIV (Nasovac-S, Serum Institute of India, Pune, India) was used in both years, containing 2009 pandemic H1N1 (A/17/California/2009/38 in 2017 and A/17/New York/15/5364 in 2018), H3N2 (A/17/Hong Kong/2014/8296), and influenza B-Victoria (B/Texas/02/2013) viruses. Nasopharyngeal samples were collected by means of flocked paediatric swabs stored in RNAprotect (Qiagen, UK) at day 0 (pre-LAIV), day 2, day 7, and day 21. Samples were processed within 4 h of collection and stored at −70°C until further processing. The occurrence of adverse events since the last visit were recorded at day 7 and day 21.

DNA was extracted from nasopharyngeal by means of the AGOWA Mag Mini DNA extraction kit (LGC Genomics, Berlin, Germany) in combination with phenol–bead beating as previously described.^17^ A quantitative real-time PCR targeting the conserved autolysin (*lytA*) gene of *S pneumoniae* was used to detect and quantify pneumococcal loads in all samples, with a cycle threshold (Ct) value of less than 40 considered positive.^18^ Bacterial quantities were estimated by means of a standard curve prepared from six 1:10 serial dilutions of a *S pneumoniae* strain of known concentration, ranging from 1 ng/μL to 0·00001 ng/μL (441 000 to 4·41 genome copies per μL).

A multiplex respiratory virus real-time PCR was done as previously described on all day 0 samples to detect influenza A, influenza B, respiratory syncytial virus (RSV) A, RSV B, human parainfluenza viruses (HPIV) 1–4, human metapneumovirus, adenovirus, seasonal coronaviruses (CoV; 229E, OC43, NL63), and human rhinovirus.^19^ Day 7 samples from control group participants were also tested for a post-hoc analysis. Shedding of LAIV strains was assessed on day 2 and day 7 by means of a monoplex RT-PCR, with haemagglutinin-specific primers and probes as previously described.^20^

### Outcomes

The density of *S pneumoniae* as established by quantitative PCR at day 7 and day 21, compared with day 0, was defined as a primary outcome in the clinical trial protocol. Exploratory outcomes were LAIV strain shedding (day 2 and day 7) and the prevalence of asymptomatic respiratory viruses at baseline.

### Statistical analysis

Sample size was calculated on the basis of previously described estimates by means of a stochastic-simulation approach that takes into account variance in bacterial prevalence and densities.^21^ At a baseline pneumococcal carriage prevalence of 75% and accounting for 10% loss to follow-up, 220 LAIV-immunised participants would provide >99% power at an alpha of 0·05 to detect a two times increase in colonisation density following LAIV.

Children completing their day 21 visit with available samples were included in the final modified intention-to-treat analysis. χ^2^ or Fisher's exact tests were used to compare *S pneumoniae* carriage prevalence and adverse events between groups at each timepoint. In addition, generalised logistic mixed-effect models were used to explore the change in carriage prevalence over time within each group.

Pneumococcal colonisation densities were transformed to log_10_ +1 copies per μL for analysis. Differences in colonisation densities between groups per timepoint were assessed by means of the Mann-Whitney *U* test (for data distributions see the appendix p 10). Generalised linear mixed-effect models were used to study the association between colonisation densities (response variable), LAIV administration, and other covariates, with a random effect on participants. In order to capture the effect of new *S pneumoniae* acquisition during the study period, all participants were included and not just those with positive pneumococcal PCR at baseline. A multivariable model was initially built containing all covariates of interest and possible interactions, including those suspected to be associated with *S pneumoniae* colonisation densities to understand vaccine effects, potential confounders, and their estimated effect sizes. Stepwise backwards selection was used to arrive at the most parsimonious model, with comparisons between models done by means of likelihood ratio tests (appendix p 3). Covariates with a p value of <0·05 were deemed significant. Additional exploratory analyses were done by fitting a series of stratified, post-hoc models to obtain more detailed insight into the relationship between pneumococcal density and the same covariates in specific groups (eg, LAIV recipients or controls). All analyses were done by means of R software (version 3.5.1). No data monitoring committee was used. The study is registered at ClinicalTrials.gov, NCT02972957.

### Role of the funding source

The funder had no role in study design, data collection, data analysis, data interpretation, or writing of the report.

## Results

**Figure 1:**
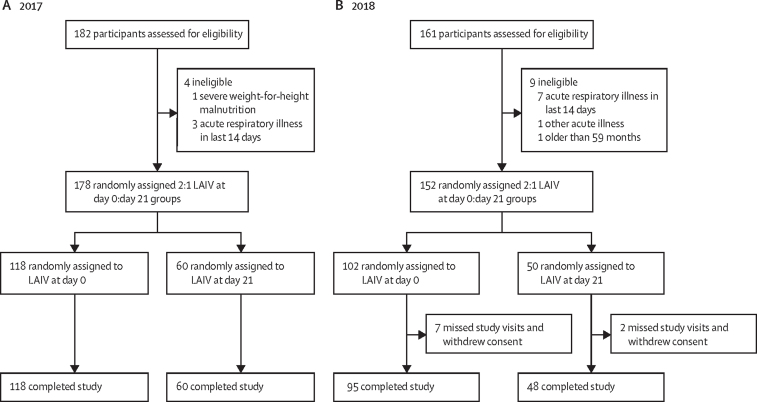
Trial profile Overview of participants recruited and retained in the study. Recruitment in 2017 (A). Recruitment in 2018 (B). Children randomly assigned to the LAIV group received one dose of Russian-backbone trivalent LAIV containing either A/17/California/2009/38 in 2017 or A/17/New York/15/5364 in 2018 at day 0. H3N2 strain and influenza B lineages included in the vaccine remained consistent across the 2 years. LAIV=live attenuated influenza vaccine.

Between Feb 8 and April 12, 2017, 178 children of 182 assessed for eligibility were recruited and randomly assigned to receive LAIV at day 0 (LAIV group; n=118) or day 21 (control group; n=60). Between Jan 15 and March 28, 2018, 152 children of 161 assessed for eligibility were randomly assigned to the LAIV (n=102) or control (n=50) groups. All children in 2017 and 143 (94%) in 2018 completed the study (figure 1). All study visits were within protocol-defined windows (+1 day for day 2, +7 days for day 7, +7 days for day 21) and 96% were on the exact visit day. In the LAIV group, samples were available for *lytA* PCR in 211 of 213 samples at day 0 and all samples at day 7 and day 21, whereas in the control group, samples from all 108 children were available from all three timepoints.

Baseline characteristics were similar between the two groups, with a pneumococcal carriage prevalence of 74·9% at day 0 (table 1). Of the children in the LAIV group who had D0 samples available for respiratory viral RT-PCR, 67 (33%) of 205 had an asymptomatic viral infection, compared with 41 (39%) of 104 in the control group. Overall, 82 (76%) of 108 of these infections were due to a rhinovirus (table 1).

**Table 1:** Baseline characteristics

		**Live attenuated influenza vaccine (n=213)**	**Control (n=108)**
Age, months		36·4 (28·4–42·4)	35·1 (27·6–40·3)
Sex			
	Male	116 (55%)	58 (54%)
	Female	97 (46%)	50 (46%)
Baseline asymptomatic virus present*		67 (33%)	41 (39%)
	Adenovirus	3 (4%)	3 (7%)
	Seasonal coronaviruses	7 (10%)	3 (7%)
	Parainfluenza 1	2 (3%)	4 (10%)
	Parainfluenza 1 and seasonal coronaviruses	1 (1%)	0
	Parainfluenza 1 and rhinovirus	1 (1%)	0
	Parainfluenza 3	0	1 (2%)
	Rhinovirus	50 (75%)	25 (61%)
	Rhinovirus and adenovirus	1 (1%)	3 (7%)
	Rhinovirus and seasonal coronaviruses	1 (1%)	1 (2%)
	Respiratory syncytial virus A	1 (1%)	0
	Respiratory syncytial virus B	0	1 (2%)
Presence of smoker in the house		51 (24%)	21 (19%)
Outdoor cooking area†		189 (89%)	97 (90%)
Baseline *Streptococcus pneumoniae* colonisation‡		158 (75%)	81 (75%)

Data are median (IQR) or n (%). Percentages might not sum to 100 due to rounding.

* 205 children in LAIV group and 104 children in control group had day 0 data available for respiratory viral co-infection. These data were not available for eight children in the LAIV group and four children in the control group due to the lack of sample availability for respiratory viral assays. Percentages given for individual or combinations of viruses detected use the total number of children with asymptomatic viruses detected for each group as the denominator.

† Cooking inside (under a roof) compared with cooking using an indoor kitchen. Note that 99% of children lived in households where wood or charcoal was the primary fuel used to cook regardless of location of cooking.

‡ *Streptococcus pneumoniae* detection was done by *lytA* PCR; *S pneumoniae positive* samples were defined as having a cycle threshold value of <40 on *lytA* PCR. For the LAIV group, 211 of 213 day 0 samples were available for testing.

The difference in prevalence of *S pneumoniae* colonisation was not significant between groups at day 7 (174 [82%] of 213 LAIV and 83 [77%] of 108 control, p=0·38) or day 21 (180 [85%] of 213 LAIV and 85 [79%] of 108 control, p=0·25, table 2). However, by means of a logistic mixed-effect regression model to take into account within-individual changes over time, the presence of pneumococcal colonisation was higher at day 7 (p=0·042) and day 21 (p=0·0037) compared with day 0 in the LAIV group (table 3), but not in the control group (p=0·70 for day 7 *vs* day 0; p=0·44 for day 21 *vs* day 0, appendix p 4). Similarly, when comparing median pneumococcal density between each group at each timepoint, no significant differences were observed (table 2). Generalised linear mixed-effect regression models were done to study the effect of LAIV and other covariates on the dynamics of pneumococcal density. We observed a significant association between LAIV administration at day 0 and higher *S pneumoniae* colonisation density during the study period (p=0·0097, appendix p 4), adjusting for year of recruitment, presence of baseline asymptomatic viruses, age, and household cooking practice. Increases in *S pneumoniae* density were also seen with baseline asymptomatic viral infection (p=0·017), younger age (p=0·0030), day 21 of study (p=0·0015), and recruitment in 2018 (p=0·0091). However, a significant interaction was seen between year of recruitment and group (appendix p 4, p=0·016).

**Table 2:** *Streptococcus pneumoniae* carriage rate and colonisation density during study period

	**LAIV group**	**Control group**	p value*
S pneumoniae **carriage prevalence**			
Day 0	158/211 (75%)†	81/108 (75%)	1·00
Day 7	174/213 (82%)	83/108 (77%)	0·38
Day 21	180/213 (85%)	85/108 (79%)	0·25
***S pneumoniae* colonisation density, log_10_ copies per μL**			
Day 0	1·17 (0·02–2·08)	1·21 (0·06–2·08)	0·73
Day 7	1·43 (0·44–2·32)	1·18 (0·05–2·32)	0·084
Day 21	1·24 (0·34–2·49)	1·34 (0·16–2·49)	0·80

Data are n (%) or median (IQR).

* Comparisons of carriage rates between groups done using the χ^2^ test. Cross-sectional comparisons of *S pneumoniae* density between groups at each timepoint were done using the Mann-Whitney *U* test.

**Table 3:** Factors associated with *Streptococcus pneumoniae* carriage prevalence in the live attenuated influenza vaccine group

	**Odds ratio**	**95% CI**	p value*
Asymptomatic respiratory virus at day 0	2·03	0·93–4·43	0·076
Day 7 (*vs* day 0)	1·82	1·02–3·24	0·042
Day 21 (*vs* day 0)	2·44	1·34–4·47	0·0037
Age, months	0·96	0·92–0·99	0·030
Household cooking indoors (*vs* outdoors)†	0·49	0·17–1·45	0·20

*S pneumoniae* detection was done by lytA PCR.

* p values for factors associated with *S pneumoniae* carriage prevalence in the live attenuated influenza vaccine group are derived from a generalised logistic mixed-effect model, taking into account changes within individuals over time. Backwards model selection was done as described in the appendix before deciding on variables to include in the optimum model. Reference levels for each variable are given within brackets.

† Cooking inside (under a roof) compared with cooking using an indoor kitchen. Note, 99% of children lived in households in which wood or charcoal was the primary fuel used to cook regardless of location of cooking.

To investigate this interaction, generalised mixed-effect models were stratified by study group (LAIV or control, table 4; appendix p 5). A significant increase in pneumococcal density at day 21 was seen in the LAIV group (+0·280 log_10_ copies per μL, SE 0·105, p=0·0082, table 2), adjusting for year of recruitment, presence of asymptomatic viruses at baseline, age, and household cooking practice; but not in the control group (appendix p 5). No effect of year of recruitment was seen in the LAIV group (p=0·61, table 4), but greater *S pneumoniae* densities were observed in the control group recruited in 2018 compared with 2017 (+0·402 log_10_ copies per μL, p=0·018, appendix p 5). These differences between control groups recruited in 2017 and 2018 were confirmed in generalised mixed-effects models stratified by year of recruitment (appendix pp 5–6).

**Table 4:** Factors associated with *S pneumoniae* colonisation density in the live attenuated influenza vaccine group

	**Change in log_10_ copies per μL**	**SE**	p value*
Asymptomatic respiratory virus at day 0	+0·287	0·122	0·020
Day 7 (*vs* day 0)	+0·207	0·105	0·050
Day 21 (*vs* day 0)	+0·280	0·105	0·0082
Recruitment in 2018 (*vs* 2017)	−0·059	0·116	0·61
Age, months	−0·019	0·006	0·0020
Household cooking indoors (*vs* outdoors)†	−0·290	0·185	0·12

*S pneumoniae* detection was done by *lytA* PCR.

* p values for factors associated with *S pneumoniae* density in the live attenuated influenza vaccine group are derived from a generalised linear mixed-effect model, taking into account changes within individuals over time. Backwards model selection was done as described in the appendix before deciding on variables to include in the optimum model. Reference levels for each variable are given within brackets.

† Cooking inside (under a roof) compared with cooking using an indoor kitchen. Note, 99% of children lived in households in which wood or charcoal was the primary fuel used to cook regardless of location of cooking.

**Figure 2:**
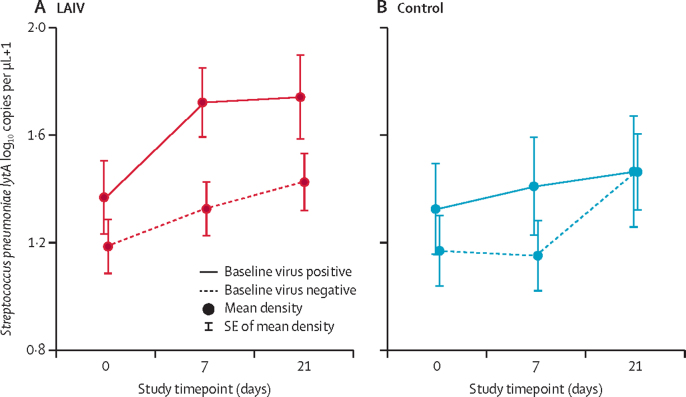
Effect of the presence of asymptomatic respiratory viral infection at baseline on *Streptococcus pneumoniae* densities during the study period in the LAIV group (A) and the control group (B) LAIV=live attenuated influenza vaccine.

The presence of an asymptomatic viral infection at baseline was associated with higher pneumococcal density during the study period in LAIV recipients (+0·287 log_10_ copies per μL, p=0·020, table 4) but not in the control group (+0·135 log_10_ copies per μL, p=0·41, appendix p 4). In the LAIV group, the presence of an asymptomatic respiratory virus at baseline resulted in an earlier peak in pneumococcal density at day 7 (figure 2A).

The greater pneumococcal density in the control recruited in 2018 appeared to be seen mainly in children with no respiratory viruses detected at day 0, and due to an increase between day 7 and day 21 (+0·311 log_10_ copies per μL, SE 0·155, p=0·050, figure 2B and appendix p 6). This probably explains the interaction seen in our main analysis between group and year on the effect of LAIV on pneumococcal density. Because asymptomatic viral infections at baseline in control group participants were not significantly different in 2017 (24 [40%] of 60) compared with 2018 (17 [39%] of 44, p=0·93), we hypothesised that this unexpected increase in pneumococcal density in the control group between day 7 and day 21 in 2018 might have been due to acquisition of new respiratory viral infections between day 0 and day 7, leading to greater pneumococcal proliferation by day 21. Of samples available for testing, 10 (21%) of 48 children in the 2017 control group had acquired a new viral infection by day 7, compared with 17 (40%) of 42 children in the 2018 control group (p=0·07, appendix p 7). Overall, (20 [54%] 37) new viral infections were due to a rhinovirus infection. The median day 21 pneumococcal density in children who acquired a new viral infection was 1·93 log_10_ copies per μL (IQR 0·92–2·65) compared with 1·34 log_10_ copies per μL (0·03–2·15, p=0·12) in those who did not. Because of unavailability of day 7 samples from the LAIV group for further testing, this analysis was done by use of only samples from the control group.

**Figure 3:**
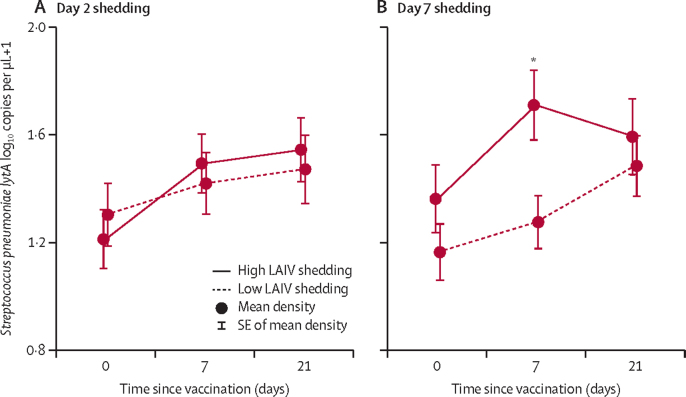
Effect of LAIV viral shedding on *Streptococcus pneumoniae* densities Mean densities of *S pneumoniae* in LAIV recipients stratified by LAIV shedding at (A) day 2 or (B) day 7. High LAIV shedding was defined as a Ct value equal to or lower than the median Ct-value in any of the three LAIV strains at each timepoint. Values above the median Ct value were classified as low LAIV shedding. All individuals with no shedding detected at each timepoint were included in the low shedding group. Note, lower Ct values denote higher amounts of virus. LAIV=live attenuated influenza vaccine. Ct=cycle threshold. *A significant association between high day 7 LAIV shedding and day 7 pneumococcal density seen in generalised linear models (+0·380 log_10_ copies per μL compared with low LAIV shedding at day 7, p=0·024, appendix p 7), but not between day 2 LAIV shedding and day 7 pneumococcal density (+0·036, p=0·83, appendix p 8).

We next investigated whether the degree of LAIV strain viral shedding was a potential driver of the observed increase in pneumococcal densities. We have previously described the detailed shedding dynamics observed in this cohort, with 184 (86%) of 213 children at day 2 and 154 (72%) of 213 at day 7 shedding at least one strain.^22^ Children were categorised as high viral shedders if the Ct value in any of the three strains was equal to or below the median Ct value of all RT-PCR values at each timepoint. Pneumococcal densities among high and low day 2 shedders were similar (figure 3A). In a generalised linear model including baseline asymptomatic respiratory viruses, year of recruitment and age as covariates, high LAIV viral shedding at day 7 was associated with higher day 7 pneumococcal density (+0·380 log_10_ copies per μL compared with low viral shedding, SE 0·167, p=0·024, figure 3B, appendix p 7). This association was not seen between day 2 LAIV shedding and day 7 pneumococcal density (appendix p 8).

Finally, a significant increase in rhinorrhoea was reported in the LAIV group compared with the control group children during the first 7 days of the study (103 [48%] of 213, compared with 25 [23%] of 108, p<0·0001), and between day 7 and 21 (108 [51%] of 213, compared with 28 [26%] of 108, p<0·0001). No other adverse events were significantly increased in the LAIV group (appendix pp 8–9).

## Discussion

We report what we believe is the first study exploring the effect of LAIV on pneumococcal density in children living in an LMIC with a high prevalence of *S pneumoniae* carriage. We found a modest increase in pneumococcal density following LAIV administration, supporting our initial hypothesis. Although these findings are consistent with the only other such study among children in the UK,^10^ the increase in density we observed was less (1·78-times the original magnitude by day 21) than the six-times increase (at day 28) in genome copies per mL reported by Thors and colleagues.^22^ Furthermore, the increase in our study was mainly seen between day 0 and 7 (1·58-times), consistent with the peak seen in a mouse model,^9^, ^10^ and appeared to be driven by higher viral shedding and co-infection with asymptomatic respiratory viruses at baseline.

The reasons for this contrast with the study of UK children are not entirely clear. Our study used the trivalent Russian-backbone LAIV whereas Thors and colleagues used a trivalent Ann Arbor LAIV.^10^ Given the influence of the high LAIV shedding on pneumococcal density we observed, and the effect of pre-existing influenza antibodies on shedding we have previously shown,^20^ it is possible that differences in either LAIV formulation or previous influenza history in the two cohorts could have played a role. Although it is possible that had we extended our timepoints to day 28 we might have seen a similar rise, our observed dynamics with the greatest increase between day 0 and 7 would suggest otherwise. Finally, although comparison of pneumococcal densities across the two studies should be done with caution, it is striking that our baseline pneumococcal density in the LAIV group (1·26 log_10_ copies per μL or 18 197 copies per mL) was higher than that in the UK study even following the six-times rise (16 687 copies per mL). Little is known about the plausible dynamic range of pneumococcal colonisation in the paediatric nasopharynx, but there might be limits to how much perturbation is possible if density is already very high and at relative equilibrium with other microorganisms.

We observed that the presence of an asymptomatic respiratory viral infection at baseline was associated with an earlier peak and higher pneumococcal densities following LAIV. Respiratory viruses are common in asymptomatic children,^23^, ^24^ albeit data are scarce from LMICs. In a 52-week household transmission study in Utah, 79 (31%) of 254 of viral episodes in 21 children younger than 5 years were asymptomatic.^23^ In a Dutch cohort, 250 (83%) of 303 healthy asymptomatic children (median age 14·1 months) had at least one respiratory virus detected, with rhinovirus being most prevalent.^24^ The prevalence of asymptomatic viruses detected in our cohort was lower (35%), but in slightly older children who were deliberately recruited during the dry season in The Gambia when less respiratory viral infections occur. Interestingly, the trajectories of pneumococcal density during the study were very similar between LAIV recipients with no baseline respiratory virus compared with control group participants who had asymptomatic viruses detected at day 0. This suggests that the effect of LAIV on pneumococcal density might be similar to any other mild respiratory viral infection. Importantly, our findings show that the effect of asymptomatic viruses on pneumococcal density is enhanced with cumulative infections, whether acquired naturally or introduced in attenuated forms such as LAIV. We detected a new respiratory viral infection in 27 (30%) of 90 children in the control group at day 7 of the study. This highlights how frequently the nasopharynx of young children is subjected to new viruses and therefore the dynamic nature of viral–bacterial interactions that must exist in this biological niche. Increases in pneumococcal density have been shown in children aged 48–96 months during both symptomatic and asymptomatic acute respiratory viral infections.^25^ Another study in children aged 3 months–6 years old showed pneumococcal carriage prevalences to be higher during symptomatic (37·1%) compared to asymptomatic (23·6%) respiratory viral infections.^26^

Lastly, LAIV receipt was associated with increased rhinorrhoea in our study. Elevated pneumococcal colonisation density during episodes of viral rhinitis in children can potentially facilitate pneumococcal transmission.^22^ It is possible that prolonged nasal discharge combined with the modest increase in pneumococcal density could together increase the chances of pneumococcal transmission in this setting. This could be explored in studies specifically measuring *S pneumoniae* acquisition in household members of children receiving LAIV.

Our study has several strengths. The randomised, controlled design and longitudinal nature allowed us to compare bacterial dynamics over time, enabling us to quantify the effect of LAIV on pneumococcal density while minimising confounding effects. High retention of study participants and adherence to study visit dates greatly reduced variability. Our study was adequately powered to detect at least a 2-times increase in pneumococcal density following LAIV. A 1·5–2·0-times increase has been suggested as being at the lower end of a clinically meaningful increase with respect to enhanced transmission potential.^21^

Our study has limitations. We used a qPCR to detect and quantify *S pneumoniae* and therefore could not establish the burden of viable bacteria. Nevertheless, a strong correlation between culture and *lytA* qPCR in quantifying *S pneumoniae* is reported.^27^ Although we provide an effect of LAIV on the overall pneumococcal population, analysing the effect on specific PCV and non-PCV *S pneumoniae* serotypes would be important. We also observed an association between higher day 7 LAIV shedding and greater pneumococcal density at that timepoint. This is in keeping with a hypothesis that greater viral burden might drive more bacterial proliferation, whether due to higher viral load of a single virus or co-infections with several viruses. However, as both LAIV and *S pneumoniae* quantification was done by means of a single swab, we cannot exclude that this association was an artefact of variability in sample material collected with each swab.

Our study was not powered to detect a difference in pneumococcal carriage rates between groups. However, we did observe an increase in pneumococcal colonisation over time in the LAIV group. It was not possible for us to establish whether this was due to an increase in pre-existing *S pneumoniae* below the level of detection of our qPCR assay or new acquisitions. Of note, human co-challenge study in adults has observed that receipt of LAIV 3 days before pneumococcal challenge increased the *S pneumoniae* carriage prevalence detected by molecular methods (60% compared with 40% in controls).^11^ Our study was also not designed to establish how long the effect of either LAIV or other acute respiratory viral infections on pneumococcal density might last. We did not know when the asymptomatic respiratory viruses detected at baseline might have occurred. An LAIV challenge study with longer follow-up or a longitudinal cohort study characterising the effect of asymptomatic and symptomatic respiratory viral infections on bacterial dynamics would be valuable.

We did an individually randomised study, which theoretically might underestimate the effect on children in the control group as higher pneumococcal densities in vaccinated children might increase transmission in shared environments. Although this might be an issue when recruiting from day care centres,^9^, ^10^ it is less likely to be relevant in our community-based study in which young children spend most days within and around their own households. We recruited 20 sibling pairs, with only six pairs in which one sibling was in the LAIV group and the other in the control group during the same year. Finally, we were unable to fully explain the unexpected increase in pneumococcal density between days 7 and 21 in control group participants recruited in 2018 which confounded our comparisons between groups.

The Gambia introduced PCV into its Expanded Programme of Immunisation in 2009 with nonavalent PCV (PCV9) and switched to 13-valent (PCV13) in 2011.^28^ By 2014, PCV13 coverage in children under 12 months was 94%.^29^ Despite vaccine-attributed reduction of pneumococcal pneumonia and invasive pneumococcal disease,^28^, ^29^ *S pneumoniae* carriage remains high with concurrent rapid replacement of PCV-serotypes by non-vaccine serotypes.^30^ Our findings represent, to the best of our knowledge, the first data elucidating interactions between both LAIV and naturally-acquired asymptomatic viral infections on pneumococcal density in children from an area of high pneumococcal carriage in Africa. Introduction of LAIV and other respiratory virus vaccines might be key to curb the burden of RTIs caused by both viruses and bacteria in LMICs. Although the effect of widespread LAIV rollout in settings with high pneumococcal burden on transmission of *S pneumoniae* has not been established, our data suggest that LAIV behaves like any other asymptomatic viral infection. Considering that LAIV has a wealth of safety data in young children and was well tolerated and highly accepted in our study,^13^ these findings should provide further support around use of LAIV in African countries to urgently reduce the substantial burden of influenza mobidity and mortality that exists in young children.

## Declaration of interests

We declare no competing interests.
