# Supplementary material for: The effect of live attenuated influenza vaccine on pneumococcal colonisation densities among children aged 24–59 months in The Gambia: a phase 4, open label, randomised, controlled trial
Source: Lancet Microbe. 2021 Dec;2(12):e656–65. doi: 10.1016/S2666-5247(21)00179-8 (PMC8632704; doi:10.1016/S2666-5247(21)00179-8)
Supplement: Supplementary appendix [file mmc1.pdf]

THE LANCET
Microbe

Supplementary appendix

This appendix formed part of the original submission and has been peer reviewed. We post it as supplied by the authors.

Supplement to: Peno C, Armitage EP, Clerc M, et al. The effect of live attenuated influenza vaccine on pneumococcal colonisation densities among children aged 24–59 months in The Gambia: a phase 4, open label, randomised, controlled trial. *Lancet Microbe* 2021; published online Sept 13. https://doi.org/10.1016/S2666-5247(21)00179-8.

Appendix

Table of Contents

Inclusion and exclusion criteria... 2

Model selection for statistical analysis...3

Table S1. Factors associated with pneumococcal prevalence in the Control group............4

Table S2. Factors associated with pneumococcal density in the LAIV group 4

Table S3. Factors associated with pneumococcal density in the control group 5

Table S4. Factors associated with pneumococcal density in children recruited in 2017.......5

Table S5. Factors associated with pneumococcal density in children recruited in 2018.......6

Table S6. D7 and D21 change in pneumococcal density in children without asymptomatic respiratory virus at day 0 in the control group...6

Table S7. Viral infections acquired between baseline (day 0) and day 7 in the control group. 7

Table S8. Association between day 7 LAIV shedding and day 7 pneumococcal density....... 7

Table S9. Association between day 2 LAIV shedding and day 7 pneumococcal density.........8

Table S10. Adverse events between baseline (day 0) and day 7 of the study........................8

Table S11. Adverse events between day 7 and day 21 of the study.......................................9

Figure S1. Pneumococcal density data distribution...10

Clinical trial protocol..11

Inclusion Criteria

- Healthy male or female child at least 24 months of age and less than 60 months of age at the time of study entry.
- Resident in the study area and with no plans to travel outside the study area during the period of subject participation.
- Informed consent for the study participation obtained from a parent (or guardian only if neither parent is alive or if guardianship has been legally transferred).
- Willingness and capacity to comply with the study protocol as judged by a member of the clinical trial team.

Exclusion Criteria

- Serious, active, medical condition, including but not limited to:
  - chronic disease of any body system
  - severe protein-energy malnutrition (weight-for-height Z-score of less than -3)
  - known genetic disorders, such as Down’s syndrome or other cytogenetic disorder
- Active wheezing
- History of documented hypersensitivity to eggs or other components of the vaccine (including gelatin, sorbitol, lactalbumin and chicken protein), or with life-threatening reactions to previous influenza vaccinations.
- History of documented hypersensitivity to macrolide antibiotics
- History of Guillain-Barré syndrome.
- Receipt of aspirin therapy or aspirin-containing therapy within the two weeks before planned study vaccination.
- Any suspected or confirmed congenital or acquired state of immune deficiency including but not limited to primary immunodeficiencies including thymus disorders, HIV/AIDS, haematological or lymphoid malignancies.
- Any current immunosuppressive/immunomodulatory treatment or receipt of any such treatment within the six months preceding trial enrolment (for corticosteroids this is defined as a dose of prednisolone (or equivalent) of greater than 2mg/kg/day for one week or 1mg/kg/day for one month. The use of topical corticosteroids is not an exclusion criterion.
- The use of inhaled corticosteroids within the last one month.
- Receipt of an influenza vaccine within the past 12 months.
- Has any condition determined by investigator as likely to interfere with evaluation of the vaccine or be a significant potential health risk to the child or make it unlikely that the child would complete the study?
- Any significant signs or symptoms of an acute illness or infection including:

- an axillary temperature of 38.0°C or above or documented fever of 38°C or above in the preceding 14 days.
- Any acute respiratory infection within 14 days of enrolment visit.

Model selection by backwards selection for generalised linear and logistic mixed-effects models

A multivariable model was first constructed using pneumococcal density (continuous data) as the response variable and including all possible covariates of interest: Group (ref: control vs LAIV), Study timepoint (ref: D0 vs D7 and D21), year of recruitment (ref:2017 vs 2018), presence of asymptomatic respiratory viruses at baseline, age in months, sex, presence of a smoker in the household, type of cooking (re:outdoor open fire vs indoor kitchen). Interactions between group and study timepoint, group and year of recruitment, as well as group and the presence of asymptomatic respiratory viruses were also included.

Backward selection was then undertaken, removing covariates to construct a new model and comparing this reduced model with the previous model using ANOVA-derived P values and Akaike information criteria (AIC). Non-informative covariates were sequentially removed in this way to arrive at the most parsimonious model for our data. Non-significant interaction terms were first removed prior to single covariates. The final model is as stated below:

Pneumococcal density ~ study timepoint + group*year of recruitment + presence of baseline respiratory viruses + age in months + type of cooking + (1 | participant ID)

A logistic mixed-effects model with pneumococcal carriage (categorical data) as the response variable and including covariates of interest was constructed in a similar way, for each group (LAIV or control) individually. The final model is as stated below:

Presence of pneumococcal carriage ~ study timepoint + presence of baseline respiratory viruses + age in months + type of cooking + (1 | participant ID)

Table S1. Factors associated with pneumococcal prevalence in the Control group

Variable	Odds ratio	95% CI	P value#
Asymptomatic respiratory virus at day 0	0.92	0.38 – 2.24	0.86
Day 7 (vs day 0)	1.16	0.55 – 2.44	0.70
Day 21 (vs day 0)	1.35	0.63 – 2.88	0.44
Age (in months)	0.99	0.94 – 1.04	0.64
Household cooking indoors (vs outdoors)^	0.65	0.16 – 2.71	0.55

#P values for factors associated with *S. pneumoniae* prevalence are derived from a generalized logistic mixed-effect model. CI = confidence interval. Reference levels for each variable are given within brackets. ^Cooking inside (under a roof) compared to cooking using an indoor kitchen. Note 99% of children lived in households where wood/charcoal was primary fuel used to cook regardless of location of cooking.

Table S2. Factors associated with pneumococcal density during the study period

Variable	*Change in log10copies/µl*	*Standard Error*	*P value#*
LAIV receipt at Day 0 (vs control)	+0.339	0.129	0.0097
Asymptomatic respiratory virus at day 0	+0·259	0.097	0.017
Day 7 (vs day 0)	+0.153	0.082	0.081
Day 21 (vs day 0)	+0.272	0.082	0.0015
Recruitment in 2018 (vs 2017)	+0.423	0.160	0.0091
Age (in months)	-0.015	0.005	0.0030
Household cooking indoors (vs outdoors)^	-0·275	0.151	0.067
Group:year of recruitment interaction	-0.476	0.195	0.016

#P values for factors associated with *S. pneumoniae* density are derived from a generalized linear mixed-effect model. Reference levels for each variable are given within brackets. ^Cooking inside (under a roof) compared to cooking using an indoor kitchen. Note 99% of children lived in households where wood/charcoal was primary fuel used to cook regardless of location of cooking.

Table S3. Factors associated with pneumococcal density in the control group

Variable	*Change in log10copies/µl*	*Standard Error*	*P value[#]*
Asymptomatic respiratory virus at day 0	+0·135	0·164	0·41
Day 7 (vs day 0)	+0·022	0·130	0·86
Day 21 (vs day 0)	+0·232	0·130	0·076
Recruitment in 2018 (vs 2017)	+0·402	0·166	0·018
Age (in months)	+0·135	0·164	0·45
Household cooking indoors (vs outdoors)^	-0·263	0·277	0·34

[#]P values for factors associated with *S. pneumoniae* density are derived from a generalized linear mixed-effect model. Reference levels for each variable are given within brackets. ^Cooking inside (under a roof) compared to cooking using an indoor kitchen. Note 99% of children lived in households where wood/charcoal was primary fuel used to cook regardless of location of cooking.

Table S4. Factors associated with pneumococcal density in children recruited in 2017

Variable	*Change in log10copies/µl*	*Standard Error*	*Degrees of freedom*	*P value[#]*
LAIV receipt at Day 0 (vs control)	+0·323	0·127	168	0·012
Asymptomatic respiratory virus at day 0	+0·140	0·130	168	0·28
Day 7 (vs day 0)	+0·025	0·112	345	0·82
Day 21 (vs day 0)	-0·065	0·112	345	0·56
Age (in months)	-0·014	0·007	169	0·034
Household cooking indoors (vs outdoors)^	-0·160	0·246	168	0·52

[#]P values for factors associated with *S. pneumoniae* density are derived from a generalized linear mixed-effect model. Reference levels for each variable are given within brackets. ^Cooking inside (under a roof) compared to cooking using an indoor kitchen. Note 99% of children lived in households where wood/charcoal was primary fuel used to cook regardless of location of cooking.

Table S5. **Factors associated with pneumococcal density in children recruited in 2018**

Variable	*Change in log10copies/µl*	*Standard Error*	*P value*[#]
LAIV receipt at Day 0 (vs control)	-0·139	0·155	0·37
Asymptomatic respiratory virus at day 0	+0·346	0·149	0·022
Day 7 (vs day 0)	+0·295	0·116	0·012
Day 21 (vs day 0)	+0·685	0·116	<0.0001
Age (in months)	-0·016	0·008	0·042
Household cooking indoors (vs outdoors)^	-0·359	0·201	0·078

[#]P values for factors associated with *S. pneumoniae* density are derived from a generalized linear mixed-effect model. Reference levels for each variable are given within brackets. ^Cooking inside (under a roof) compared to cooking using an indoor kitchen. Note 99% of children lived in households where wood/charcoal was primary fuel used to cook regardless of location of cooking.

Table S6. **D7 to D21 change in pneumococcal density in children without asymptomatic respiratory virus at day 0 in the control group**

Variable	Change in log10copies/µl	Standard Error	P value[#]
Day 21 (vs day 7)	+0.311	0.155	0.0501
Age (in months)	-0.009	0.013	0.48
Recruitment in 2018 (vs 2017)	+0.573	0.232	0.017
Household cooking indoors (vs outdoors)^	-0.146	0.363	0.69

[#]P values for factors associated with *S. pneumoniae* density are derived from a generalized linear mixed-effect model. Reference levels for each variable are given within brackets. ^Cooking inside (under a roof) compared to cooking using an indoor kitchen. Note 99% of children lived in households where wood/charcoal was primary fuel used to cook regardless of location of cooking.

Table S7. Viral infections acquired between baseline (day 0) and day 7 in the control group.

	2017 n=48	2018 n=42	P value#
**Day 7 respiratory viral infections***	**10 (20·8%)**	**17 (40·5%)**	0·07
Adenovirus	0 (0·0%)	2 (11·8%)	
Seasonal coronaviruses (sCoV)	1 (10·0%)	3 (17·6%)	
Parainfluenza 1	0 (0·0%)	1 (5·9%)	
Influenza B & Rhinovirus	0 (0·0%)	2 (11·8%)	
Rhinovirus	8 (80·0%)	7 (41·2%)	
Rhinovirus & Adenovirus	1 (10·0%)	1 (5·9%)	
Rhinovirus & sCoV	0 (0·0%)	1 (5·9%)	

*Shown are new viruses detected at day 7 that were not present at baseline (day 0). Samples where the same virus was detected at day 7 that was present at baseline are not included (n=11). #P value derived from Fisher's exact test comparing the presence of any respiratory virus across years.

Table S8. Association between day 7 LAIV shedding and day 7 pneumococcal density.

Variable	*Change in log10copies/µl*	*Standard Error*	*P value*
Day 7 high LAIV shedding (vs low shedding)*	+0·380	0·167	0·024
Asymptomatic respiratory virus at day 0	+0·442	0·170	0·0099
Recruitment in 2018 (vs 2017)	-0·259	0·162	0·11
Age (in months)	-0·017	0·009	0·059
Household cooking indoors (vs outdoors)^	+0·006	0·257	0·98

*high LAIV shedding was defined as having at least one LAIV strain (of three measured) with a RT-PCR cycle threshold (Ct) value equal to or lower than the median Ct value of all LAIV RT-PCR data at day 7. Note lower Ct values denote higher amount of virus. ^Cooking inside (under a roof) compared to cooking using an indoor kitchen. Note 99% of children lived in households where wood/charcoal was primary fuel used to cook regardless of location of cooking.

Table S9. **Association between day 2 LAIV shedding and day 7 pneumococcal density.**

Variable	*Change in log10copies/µl*	*Standard Error*	*P value*
Day 2 high LAIV shedding (vs low shedding)*	+0·036	0·167	0·83
Asymptomatic respiratory virus at day 0	+0·42	0·172	0.015
Recruitment in 2018 (vs 2017)	-0·233	0·166	0·16
Age (in months)	-0·023	0·009	0.013
Household cooking indoors (vs outdoors)^	+0·011	0·260	0·97

*high LAIV shedding was defined as having at least one LAIV strain (of three measured) with a RT-PCR cycle threshold (Ct) value equal to or lower than the median Ct value of all LAIV RT-PCR data at day 7. Note lower Ct values denote higher amount of virus. ^Cooking inside (under a roof) compared to cooking using an indoor kitchen. Note 99% of children lived in households where wood/charcoal was primary fuel used to cook regardless of location of cooking.

Table S10. **Adverse events between baseline (day 0) and day 7 of the study**

**Adverse event**	**LAIV (n=213)**	**Control (n=108)**	**P value**
Fever	32 (15.0%)	11 (10.2%)	0.23
Sore throat	1 (0.5%)	0 (0.0%)	0.48
Chills	1 (0.5%)	0 (0.0%)	0.48
Muscle/joint pains	0 (0.0%)	0 (0.0%)	-
Headache	4 (1.9%)	0 (0.0%)	0.15
Epistaxis	0 (0.0%)	0 (0.0%)	-
Nasal congestion	7 (3.3%)	1 (0.9%)	0.20
Reduced appetite	9 (4.2%)	8 (7.4%)	0.23
Rhinorrhoea	103 (48.4%)	25 (23.5%)	<0.0001
Rash	6 (2.8%)	4 (3.7%)	0.67
Dyspnoea	1 (0.5%)	0 (0.0%)	0.48
Cough	54 (25.4%)	19 (17.6%)	0.12
Sputum production	5 (2.3%)	2 (1.9%)	0.77
Irritability	0 (0.0%)	1 (0.9%)	0.16
Vomiting	8 (3.8%)	5 (4.6%)	0.71
Lethargy	5 (2.3%)	1 (0.9%)	0.37
Diarrhoea	13 (6.1%)	12 (11.1%)	0.11
Ear pain	4 (1.9%)	0 (0.0%)	0.15

Data are n (%). P values were derived from Chi-square test or Fisher’s exact test where appropriate. LAIV=live attenuated influenza vaccine.

Table S11. **Adverse events between day 7 and day 21 of the study**

Adverse event	LAIV (n=213)	Control (n=108)	P value
Fever	39 (18·3%)	15 (13·9%)	0·32
Sore throat	3 (1·4%)	0 (0·0%)	0·22
Chills	0 (0·0%)	0 (0·0%)	-
Muscle/joint pains	0 (0·0%)	0 (0·0%)	-
Headache	4 (1·9%)	0 (0·0%)	0·15
Epistaxis	0 (0·0%)	0 (0·0%)	-
Nasal congestion	4 (1·9%)	1 (0·9%)	0·52
Reduced appetite	9 (4·2%)	8 (7·4%)	0·23
Rhinorrhoea	108 (50·7%)	28 (25·9%)	<0·0001
Rash	4 (1·9%)	2 (1·9%)	0·99
Dyspnoea	1 (0·5%)	0 (0·0%)	0·48
Cough	76 (35·7%)	30 (27·8)	0·16
Sputum production	2 (0·9%)	1 (0·9%)	0·99
Irritability	0 (0·0%)	1 (0·9%)	0·16
Vomiting	4 (1·9%)	5 (4·6%)	0·16
Lethargy	3 (1·4%)	1 (0·9%)	0·71
Diarrhoea	14 (6·6%)	8 (7·4%)	0·78
Ear pain	3 (1·4%)	0 (0·0%)	0·22

Data are n (%). P values were derived from Chi-square test or Fisher's exact test where appropriate. LAIV=live attenuated influenza vaccine.

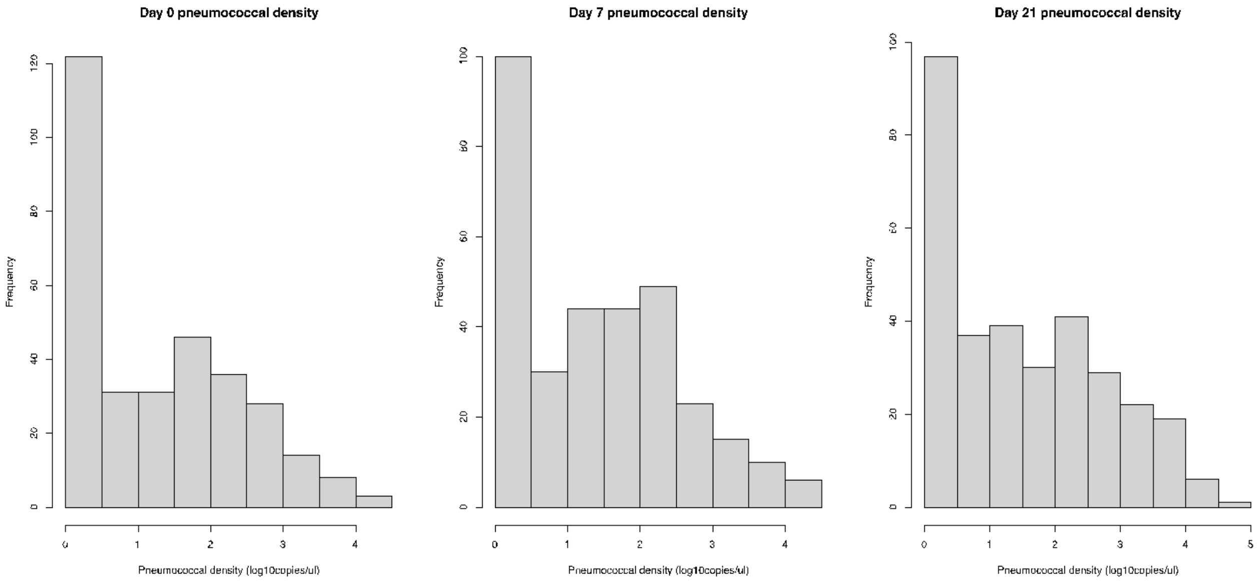

Figure S1. Distribution of *S. pneumoniae* density data at different timepoints.

Clinical Trial Protocol

A Study of Intranasal Live Attenuated Influenza Vaccine Immunogenicity and Associations with the Nasopharyngeal Microbiome Among Children in the Gambia - The NASIMMUNE Study.

**Protocol number:**	TBC
**Brief Title:**	LAIV immunogenicity and association with the nasopharyngeal microbiome (The NASIMMUNE Study)
**Protocol version and date:**	Version 1.1 5th August 2016
**Sponsor:**	Medical Research Council Unit The Gambia<br>PO Box 273 Banjul<br>The Gambia, West Africa
**Principal Investigator:**	Dr. Thushan de Silva
**Co-investigators:**	Dr. Ed Clarke<br>Prof. Beate Kampmann

Key roles

**Authors:**	Thushan de Silva<br>Visiting Scientist, Vaccines and Immunity Theme<br>MRC Unit The Gambia<br>PO Box 273, Banjul<br>The Gambia<br>T: +2207254291<br><br>Wellcome Trust Intermediate Clinical Fellow<br>Section of Paediatrics, Department of Medicine<br>Wright Fleming Institute<br>Imperial College London, St Mary's Campus<br>Norfolk Place, London W2 1PG, UK<br>T: +44(0)7976605320<br>E: t.de-silva@imperial.ac.uk<br><br>With input from MRC Unit The Gambia Clinical Trials Support Office, collaborators, lead Statistician, data management lead, Research Governance & Support Services Manager.
**Sponsor's representative:**	Jonas Lexow<br>Research Governance & Support Services Manager<br>MRC Unit The Gambia<br>PO Box 273, Banjul<br>The Gambia<br>T: +2204495442/6 Ext 2312<br>E: jlexow@mrc.gm
**Principal Investigator:**	Thushan de Silva (as above)
**Co-investigators:**	Ed Clarke<br>Head of Infant Immunology, Vaccines and Immunity Theme<br>MRC Unit The Gambia<br>PO Box 273, Banjul<br>The Gambia<br>T: +2204495442/6 Ext 4014<br>E: eclarke@mrc.gm<br><br>Beate Kampmann<br>Theme Leader Vaccines and Immunity Theme<br>MRC Unit The Gambia<br>PO Box 273, Banjul<br>The Gambia<br>T: +2204495442/6 Ext 3001
**Sub-investigators:**	Research Clinician
**Trial monitors:**	Vivat Thomas-Njie

Muhammed Camara
Gibbi Sey
MRC Unit The Gambia
PO Box 273, Banjul
The Gambia

Local Safety Monitor: **Dr. Idoko Olubukola**
MRC Unit The Gambia
PO Box 273, Banjul
The Gambia
E: oidoko@mrc.gm

Lead statistician: David Jeffries
Head of Statistics and Bioinformatics
MRC Unit The Gambia
PO Box 273, Banjul
The Gambia
T: +2204495442/6 Ext 2002
E: djeffries@mrc.gm

Data management lead: Bai Lamin Dondeh
Head of Data Management
MRC Unit The Gambia
PO Box 273, Banjul
The Gambia
T: +2204495442/6 Ext 2001

Collaborators:

Debby Bogaert
PI pathogenesis of respiratory infections group
Department of Pediatric Infectious Diseases and Immunology
Wilhelmina Children's Hospital/University Medical Center Utrecht
Utrecht, Netherlands
T: +31887551754
E: d.bogaert@umcutrecht.nl

Professor of Paediatric Infectious Diseases
University of Edinburgh Medical School
The Chancellor's Building
49 Little France Crescent
Edinbugh, EH16 4SB, UK

Martin Antonio
Director, WHO Regional Reference Laboratory (West and Central Africa) for Invasive Bacterial Diseases
MRC Unit The Gambia
PO Box 273, Banjul
The Gambia
T: +2204495442/6 Ext 3018
E: mantonio@mrc.gm

Maria Zambon
Director, Reference Microbiology Services
Public Health England
61 Colindale Avenue
London NW9 5HT, UK
T: +442083276810
E: Maria.Zambon@phe.gov.uk

Katja Hoschler
Respiratory Virus Unit
Virus Reference Department
Reference Microbiology Services
Public Health England
61 Colindale Avenue
London NW9 5HT, UK
T: +442083276312
E: Katja.Hoschler@phe.gov.uk

David Dockrell
Professor of Infectious Diseases
Co-director Florey Institute for Host Pathogen Interactions
Department of Infection, Immunity and Cardiovascular Disease
University of Sheffield School of Medicine,
K127 Royal Hallamshire Hospital,
Glossop Road,
Sheffield S10 2JF, UK
T: +441142712160
E: d.h.dockrell@sheffield.ac.uk

John Tregoning
Senior Lecturer
Mucosal Infection and Immunity Group
Section of Virology
Department of Medicine
Imperial College London, St Mary's Campus
Norfolk Place, London W2 1PG, UK
T: +442075943176
E: john.tregoning@imperial.ac.uk

Prof. Peter Ghazal
Division of Infection and Pathway Medicine
University of Edinburgh Medical School
The Chancellor's Building
49 Little France Crescent
Edinbugh, EH16 4SB, UK
T: +44 (0)131 242 6284
E: p.ghazal@ed.ac.uk

Helder Nakaya
Assistant Professor
Computational Systems Biology Laboratory
Department of Clinical and Toxicological Analyses
School of Pharmaceutical Sciences
University of Sao Paulo, Brazil
T: +1130913661/30913642
E: hnakaya@usp.br

Daniela Ferreira
Senior Lecturer, Respiratory Group Lead
Liverpool School of Tropical Medicine
Pembroke Place
Liverpool, L3 5QA, UK
T: +441517053100
E: Daniela.Ferreira@lstmed.ac.uk

Tao Dong
Professor of Immunology
MRC Human Immunology Unit
Weatherall Institute of Molecular Medicine
University of Oxford
John Radcliffe Hospital
Headington, Oxford OX3 9DS, UK
T: +441865222443
E: tao.dong@imm.ox.ac.uk

Adam Meijer
Respiratory Viruses Group
Department Respiratory and Enteric Viruses
Division Virology
Centre for Infectious Disease Research, Diagnostics and Screening (IDS) / PB22
National Institute for Public Health and the Environment (RIVM)
Antonie van Leeuwenhoeklaan 9
3721 MA Bilthoven
The Netherlands
T: +31 (0)30 2743595
E-mail : adam.meijer@rivm.nl

Immunology laboratory:

Immunology Platform Laboratory
MRC Unit The Gambia
PO Box 273, Banjul
The Gambia

Molecular biology laboratory:

Molecular Biology Platform Laboratory
MRC Unit The Gambia
PO Box 273, Banjul
The Gambia

**Influenza serology laboratory:**	Virus Reference Department<br>Reference Microbiology Services<br>Public Health England, London<br>(as above)<br>and University of Edinburgh (exact venue to be confirmed)
**Laboratories for influenza mucosal IgA & IgG assays:**	Respiratory Viruses Group<br>National Institute for Public Health and the Environment (RIVM), The Netherlands (as above).<br><br>Immunology Research Laboratory<br>MRC Unit The Gambia<br>PO Box 273, Banjul<br>The Gambia
**Microbiome sequencing laboratory:**	Bogaert laboratory<br>Exact venue to be confirmed
**Laboratories for transcriptomic assays:**	To be confirmed
**Ethics committee:**	Gambia Government/MRC Joint Ethics Committee<br>c/o MRC Unit The Gambia<br>PO Box 273, Banjul, The Gambia, West Africa<br>T: +2204495442/6 Ext 2308<br>E: ethics@mrc.gm
**National Regulatory Agency:**	The Medicines Control Agency<br>National Pharmaceutical Services<br>Ministry of Health and Social Welfare<br>Kotu, KSMD, The Gambia<br>T: +220 4466302

List of abbreviations

AE	Adverse event
AEFI	Adverse Event Following Immunization
ALRI	Acute lower respiratory infection
CRF	Case Report Form
CSR	Class switch recombination
DCF	Data Clarification Form
DMP	Data Management Plan
EC	Ethics Committee
EID	Egg Infective Dose
ELISA	Enzyme-Linked Immunosorbent Assay
EPI	Expanded Programme on Immunization
FSFV	First Subject First Visit
FW	Field Worker
GCLP	Good Clinical Laboratory Practice
GCP	Good Clinical Practice
HA	Haemagglutinin
HI	Haemagglutinin Inhibition
ICD	Informed Consent Document
ICH	International Conference on Harmonization
IFITM3	Interferon-induced transmembrane protein-3
IgA	Immunoglobulin A
IgG	Immunoglobulin G
IIV	Inactivated Influenza Vaccine
ILI	Influenza-like Illness
LAIV	Live Attenuated Influenza Vaccine
LMIC	Low and Middle Income Countries
LSFV	Last Subject First Visit
MDV	Master Donor Virus
mL	Millilitre
MRC	Medical Research Council
NA	Neuraminidase
NPS	Nasopharyngeal Swab
OTU	Operational Taxanomic Unit
PCR	Polymerase Chain Reaction
pCRF	Paper Case Report Form
PCV	Pneumococcal-Conjugate Vaccine
PD	Protocol Deviation
PI	Principal Investigator
pTfh	Peripheral T-follicular helper
QA	Quality Assurance

QC	Quality Checked
RCT	Randomized Controlled Trial
rRT-PCR	Realtime Reverse Transcriptase Polymerase Chain Reaction
SAE	Serious Adverse Event
SAGE	Strategic Advisory Group of Experts
SAP	Statistical Analysis Plan
SARI	Severe Acute Respiratory Illness
SDV	Source Data Verification
SIIL	Serum Institute of India, Pvt. Limited
SOP	Standard Operating Procedure
SSPs	Study Specific Procedures
SST	Serum Separation Tube
TMF	Trial Master File
UAT	User Acceptance Testing
UK	United Kingdom
URT	Upper Respiratory Tract
US	United States
WHO	World Health Organization

Protocol Summary

**Full Title:**	A Study of Intranasal Live Attenuated Influenza Vaccine Immunogenicity and Associations with the Nasopharyngeal Microbiome Among Children in the Gambia
**Short Title:**	The NASIMMUNE study
**Phase:**	4
**ClinicalTrials.gov identifier:**	TBC
**Population:**	Children aged 24 – 59 months
**Number of participants:**	365
**Number of centres:**	Single centre recruiting through one or more field sites
**Location of field sites:**	Sukuta Health Centre plus additional according to rate of recruitment
**Trial Duration:**	
- **Clinical phase:**	24 months
- **Whole Trial:**	36 months
**Duration for participants:**	Each participant will be enrolled for between approximately 4 and 10 months: either 21 days (n = 330) or 49 days (n = 35) active participation during recruitment months January – June 2017 and January - June 2018, followed by attendance at field site for assessment of any influenza-like illness (ILI) that occurs up to the end of October in the year of recruitment into the study
**Description of agent(s):**	One 0.5 mL intranasal dose of trivalent live-attenuated influenza vaccine (LAIV) formulated according to current WHO recommendations (Nasovac-S, Serum Institute of India Pvt. Ltd, Pune, India)<br><br>A single dose of azithromycin (liquid formulation – Zithromax®) at 20mg/kg (up to a maximum adult dose of 1g) to be given to a subset of subjects.
**Objectives:**	**Primary**<br>• To identify novel early systemic and mucosal molecular signatures following LAIV that are associated with subsequent robust nasal and oral influenza-specific immunoglobulin A (IgA) responses, in order to provide insight into the mechanisms of successful mucosal immunization<br><br>• To identify associations between nasopharyngeal microbiota and nasal and oral influenza-specific IgA

responses post-LAIV in Gambian children, to explore whether microbiome variability can explain suboptimal immune responses in some individuals to live intranasal mucosal vaccines in Sub-Saharan Africa

- To establish whether LAIV impacts the nasopharyngeal microbiome, with a specific focus on the burden of *S. pneumoniae*

Exploratory

- To identify novel early systemic and mucosal molecular signatures following LAIV that are associated with subsequent robust serum haemagglutination inhibition (HI) responses

- To identify novel early systemic and mucosal molecular signatures following LAIV that are associated with subsequent robust influenza-specific T-cell responses

- To describe the association between LAIV virus shedding at day 2 and/or 7 following immunization (as a measure of vaccine 'take') and influenza-specific mucosal IgA, serum HI and systemic influenza-specific T-cell responses to LAIV

- To explore the impact of antibiotic modulation of microbiota on LAIV-induced nasal gene expression and downstream effects on the mucosal immunogenicity of LAIV

- To establish whether LAIV increases the burden of *S. aureus* in the nasopharynx

- To explore associations between nasopharyngeal microbiome profiles and post-LAIV nasal and systemic gene expression profile changes

- To explore whether the presence of respiratory viruses in the nasopharynx at the time of LAIV administration impacts on influenza-specific mucosal IgA, serum HI and systemic influenza-specific T-cell responses to LAIV

- To establish whether an expansion in peripheral T-follicular helper (pTfh) cells occurs following LAIV

- To explore whether early systemic monocyte expansion following LAIV is associated with subsequent robust mucosal IgA responses

- To explore whether an expansion of IgA-expressing B-cells with a mucosal-homing phenotype is detectable in blood following LAIV and whether this expansion correlates with the subsequent nasal and oral influenza-specific IgA response

- To explore how interferon-induced transmembrane protein-3 (IFITM3) host gene polymorphisms may impact on early innate and later influenza-specific immune responses induced by LAIV

- To explore the correlation between systemic HI and oral fluid immunoglobulin G (IgG) responses, to assess the suitability of the latter as a proxy measure of LAIV-mediated seroconversion obtained via non-invasive means

- To explore any differences in early post-LAIV gene expression signatures and influenza-specific adaptive responses post-LAIV (nasal and oral IgA, serum HI or influenza-specific T-cell) in children who acquire laboratory-confirmed influenza following immunization and those who do not. Data will be obtained by passive surveillance and laboratory testing of nasopharyngeal swabs of children who go on to develop ILI as defined by:

    - Sudden onset of fever of ≥38°C (axillary) AND
    - either a cough or sore throat

Endpoints:

Primary endpoints

- Influenza-specific nasal IgA responses at day 21 post-LAIV
- Oral fluid IgA and IgG responses at day 21 post-LAIV
- Gene expression changes in nasal and systemic samples at day 2 following LAIV
- Nasopharyngeal microbiome profiles at baseline, day 7 and day 21 following LAIV
- Density of *S. pneumoniae* before and after LAIV as established by quantitative polymerase chain reaction (PCR)

Exploratory endpoints

- Influenza-specific serum HI responses at day 21 post-LAIV
- Influenza-specific peripheral T-cell responses at day 7 and 21 post-LAIV
- Detectable vaccine virus (A/H3N2, A/pH1N1 and B) in nasopharyngeal samples obtained at day 2 and 7 post-LAIV, as determined by real-time reverse transcriptase polymerase chain reaction (rRT-PCR)
- Nasopharyngeal microbiome profiles at baseline and at day 28 following a single dose of oral azithromycin in n = 30 individuals
- Respiratory viruses present in the nasopharynx at vaccination as determined by multiplex rRT-PCR (e.g. parainfluenza, adenovirus, coronavirus, rhinovirus)
- Frequency of pTfh cells at day 7 and 21 following LAIV
- Frequency of peripheral monocyte subsets at day 2 following LAIV
- Frequency of B-cell subsets, IgA and mucosal homing marker expression at day 7 and 21 following LAIV

- IFITM3-rs12252 genotyping (CC, CT and TT polymorphisms) by amplification and sequencing of genomic DNA
- Detectable influenza (A/H3N2, A/pH1N1 and B) in nasopharyngeal samples obtained from children presenting with ILI during follow up period
- Density of *S. aureus* before and after LAIV as established by quantitative polymerase chain reaction (PCR)

Description of Study Design:

Most objectives will be assessed via a phase 4, randomized, controlled clinical vaccine trial. A total of 330 healthy children aged 24 – 59 months will be enrolled into one of three groups of equal size (3 x 110 children):

(i) LAIV-vaccinated group 1 (blood sample at day 0, 2, 21)
(ii) LAIV-vaccinated group 2 (blood sample at day 0, 7, 21)
(iii) Unvaccinated

Group allocation will be concealed from the investigator team, using sealed opaque envelopes and block randomization stratified by sex, with a randomization sequence generated by a statistician not otherwise involved in the study.

Two LAIV-vaccinated groups are included in order to achieve several exploratory objectives, which require blood sampling at different times following vaccination, whilst minimizing the number of times children are bled. The unvaccinated subjects will serve as a control group for the primary objective of assessing the impact of LAIV on the nasopharyngeal microbiome.

An additional 35 healthy children aged 24 – 59 months will be recruited in the 2nd phase of recruitment (Jan – June 2018) to assess how modulation of the nasopharyngeal microbiome with antibiotics impacts the mucosal immunogenicity to LAIV. These subjects will be matched as closely as possible by pre-antibiotic nasopharyngeal microbiome profile [1], age and sex (in that order of preference), in a nested case-control study, to n = 35 children recruited in the main clinical trial.

1 Background information and rationale

2 Background information and significance

3 Introduction

Acute respiratory infections are a major cause of morbidity and mortality worldwide, of which influenza is a leading cause. It affects all age groups and is responsible for both annual epidemics and intermittent pandemics, but causes particularly severe infections in young children, older adults and those with underlying medical conditions. Many high income countries have recommendations in place to vaccinate these high risk groups, with annual vaccination required because of the frequent antigenic change that influenza undergoes due to evolutionary pressure.

With increasing evidence of the high burden of influenza in children residing in resource-limited countries, in 2012, the WHO Strategic Advisory Group of Experts (SAGE) recommended influenza vaccination in key high-risk groups in these settings, including in children aged 2 – 5 years [2]. Based on data showing a higher efficacy against influenza infections of LAIV over inactivated vaccines in children (mainly from studies conducted in high income countries), the WHO recommend LAIV as the preferential vaccine in this age group. The limited data from Sub-Saharan Africa, however, suggests relatively low efficacy of LAIV against antigenically-matched strains when compared to similar studies in European and US cohorts [3-6] and mucosal immunogenicity may also be lower than expected [7]. In addition, the exact mechanisms by which LAIV induces protective mucosal and systemic immunity are not known. To optimize responses in future generations of influenza vaccines, more detailed insights into these mechanisms are required.

4 Influenza in sub-Saharan Africa

Both the burden of infection and use of influenza vaccines in children from Sub-Saharan Africa have been neglected. Partly driven by the 2009 pandemic of Influenza A (pdmH1N1), several recent efforts have attempted to fill the knowledge gap in influenza epidemiology in Africa. This includes scaling up of influenza surveillance through expanding the number of sentinel sites in Africa, both via the newly established African Network for Influenza Surveillance and Epidemiology (ANISE) and the WHO reporting system FluNet [8]. In a study including data from 15 African countries that conducted at least one completed year of surveillance between 2006 and 2010, the overall proportion of influenza positivity was 21.7% in influenza-like illness cases (ILI) and 10.1% in cases of severe acute respiratory infection (SARI) [9]. The burden of infection was greatest in children <5 years of age.

It is possible that a combination of poor nutrition and socioeconomic conditions, co-infections and poor access to healthcare result in seasonal influenza infections playing a greater role in respiratory disease-related morbidity and mortality than in developed countries [10]. A meta-analysis published in 2011 estimated that there were 90 million influenza cases and 1 million cases of influenza-associated SARI episodes in 2008 in children <5 years globally, with the rate of severe illness in resource-limited settings being almost double that of more industrialized countries [11]. This study also demonstrated that overall, 99% of of influenza-related cases of SARI among children <5 occur in low- and middle-income countries (LMIC).

A more recent systematic analysis of the global role and burden of influenza in paediatric hospitalizations based on datasets from 60 countries in all WHO regions found that the per capita influenza-associated hospitalization rate in children <5 years of age was 174 per 100,000 per year in Africa as compared to 53 per 100,000 per year in Europe [12].

Senegal is one of the only African countries to have regularly contributed to influenza surveillance by reporting to the WHO Flunet program, with sentinel surveillance on-going in Dakar for over 15 years. This data suggests that influenza A H3N2, pdmH1N1 and influenza B are all co-circulating in the subregion, with varying proportions year by year [13]. Molecular studies reveal that the strains identified broadly reflect those circulating globally. Although infections are found throughout the year, the peak influenza transmission season in Senegal is during the rainy season around July to October each year [13, 14].

Surveillance data collected through sentinel health centres in The Gambia within the Pneumonia Etiology Research for Child Health (PERCH) study revealed that hospitalization with influenza-associated severe pneumonia was 190 per 100,000 in children <5 and 488 per 100,000 in children between 6 – 12 months [15]. Furthermore, our preliminary sero-epidemiological data from children aged 18 months old (n = 113) in 2010 from Sukuta, the peri-urban area in the Gambia where the NASIMMUNE study will be based, shows that by this age 24%, 94% and 93% of children were exposed to pdmH1N1, H3N2 and influenza B viruses respectively (de Silva, unpublished). These data emphasize that the burden of influenza infections in Gambian children is likely to be high and broadly reflect the strain circulation data available from Senegal.

5 Live Attenuated Influenza Vaccines and prevention of influenza

There are two broad types of influenza vaccines: inactivated influenza vaccine (IIV), administered usually via an intramuscular injection and live attenuated influenza vaccines, which have been formulated as intranasal preparations. Both are updated yearly, in accordance with WHO recommendations, to contain recombinant strains with haemagglutinin (HA) and neuraminidase (NA) components that match as best possible predicted circulating influenza A (H1N1, H3N2) and influenza B strains in the following season. LAIV contains cold-adapted viral strains that replicate in the cooler upper respiratory tract (URT) but not in warmer temperatures of the lung [16, 17]. It relies on viral replication to evoke an immune response that mimics natural immunity from wild-type influenza infection (unlike IIV). Both IIV and LAIV are widely used in the United States (US) and other high income countries, but at present seldom used routinely in lower resource settings.

There are currently two different LAIVs used globally, based on different attenuated strains. One, developed in the US and marketed as FluMist (or Fluenz in the United Kingdom, UK) by Medimmune, is based on the A/Ann Arbor/6/60 strain. The other, developed in the former Soviet Union from the A/Leningrad/134/57 strain is manufactured in Russia by Microgen. These strains are referred to as a master donor viruses (MDV) and each year, recombinant vaccine viruses are manufactured to contain internal structural proteins from the attenuated MDV and HA and NA from predicted circulating strains.

Recently, via a programme facilitated by the WHO, seed strains from the Russian LAIV were provided to developing country vaccine manufacturers, with the intention of scaling up provision of LAIVs to resource-limited settings [18]. The Serum Institute of India, Ltd, (SIIL) has since developed both monovalent pandemic H1N1 (Nasovac) and trivalent (Nasovac-S) LAIVs which have both since been licensed for use in India and received WHO prequalification for procurement by UN agencies.

6 US-derived LAIV (FluMist/Fluenz, Medimmune)

FluMist was first licensed for use in persons aged 5 to 49 in the US in 2003, with the license extended to children aged 2 – 4 since 2007. FluMist has demonstrated high efficacy in children, including against drifted strains of influenza with a mismatch between the vaccine and circulating strains [19]. A meta-analysis of 6 placebo-controlled studies including over 10,000 children aged 6 months to 7 years showed a pooled vaccine efficacy of 83% (95% CI 69 – 91) [20]. Three RCTs have also demonstrated superior efficacy of FluMist over IIV in children, with a meta-analysis of these and one non-randomized study estimating 46% fewer cases of influenza in children receiving LAIV compared to IIV [21].

The few studies of US-derived LAIV conducted in LMIC have yielded mixed results. A randomized-controlled trial (RCT) recruiting children from Asia and South America showed reasonable seroconversion rates with LAIV in [22]. Only one large placebo-controlled RCT of FluMist conducted in South Africa and South America has, to date, included subjects from sub-Saharan Africa and demonstrated a vaccine efficacy of 73.5% against antigenically similar influenza strains following 2 doses of LAIV, compared to the 93% demonstrated in US [5] and 85.4% in European/Israeli cohorts [6].

The safety profile of FluMist has been extensively studied both pre and post-licensure, with FluMist being associated with only mild increases in symptoms such as a runny nose, nasal congestion, headache, low-grade fever and myalgia. A large post-licensure evaluation in children aged 24 – 59 months has also not revealed any significant safety concerns with FluMist in this age group [23]. The license for US-derived LAIV in the US and UK in children is for children aged 2 years and above.

7 Russian derived-LAIV (Ultravac®, Microgen)

The Russian-derived LAIV based on the A/Leningrad/134/57 MDV has been widely used in Russia for decades, with over 120 vaccine trials including >500,000 adult and >130,000 child participants [24]. These have all demonstrated that the vaccine is safe and immunogenic in children. Although a paucity of studies using virologic endpoints, as well as heterogenous study designs including both children and adults make a robust meta-analysis difficult, unpublished data from PATH estimate a pooled vaccine effectiveness of 0.64 (95% CI 0.58 – 0.71) to decrease influenza-like illness based on five published efficacy studies (John C. Victor, PATH, personal communication) [24-28].

8 Russian derived-LAIV produced by SIIL (Nasovac-S)

Following donation of Russian-derived LAIV MDV seed strain [18], in 2009, SIIL developed a monovalent pandemic H1N1 LAIV (Nasovac), which following Phase I and II/III safety and immunogenicity studies was licensed for in use persons aged 3 and over in 2010. It is estimated that approximately 2.5 million doses of Nasovac were administered in children and adults in India without any significant safety signal. A post-licensure surveillance study conducted in n = 7565 children and adults [29] revealed mild to moderate URT symptoms in only 0.65% subjects, similar to those observed with FluMist (noted above). No serious adverse events or death related to Nasovac were observed. Vaccine efficacy against laboratory-confirmed influenza, estimated by a test-negative case control study design and logistic regression was 75.5% (95% CI 42.1 – 89.7) [30].

SIIL went on to develop a trivalent LAIV based on the Russian derived-LAIV MDV (Nasovac-S), incorporating influenza A/H1N1, A/H3N2 and influenza B strains and has been undergoing

safety, immunogenicity and efficacy studies. A phase II randomized placebo-controlled study in 300 children aged 24 – 59 months in Bangladesh showed similar solicited reactions between vaccine and placebo and no LAIV-related serious adverse events (SAEs) [31]. There was also no difference in protocol-defined wheezing between the LAIV and placebo groups. The final immunogenicity data are awaited from this study. One dose of Nasovac-S for prevention of influenza in persons aged 2 and over was licensed and launched in India in July 2014. Nasovac-S was granted a WHO prequalification certificate in 2015, opening the market for this vaccine in the PAHO region and other UNICEF countries. Nasovac-S will be manufactured and marketed by SIIL with WHO recommended influenza strains for the 2016/2017 season.

Two placebo-controlled efficacy studies using Nasovac-S were undertaken in Bangladesh and Senegal commencing in 2013 (children aged 24 – 59 months and 24 – 71 months respectively), providing mixed and contrasting results. In Senegal, N= 1174 children were given Nasovac-S, which was well tolerated with no safety concerns and equivalent numbers of mild adverse effects as the placebo arm, including no increased incidence of protocol-defined wheezing (John C. Victor, XVII International Symposium on Respiratory Viral Infections**,** Vancouver, CA, 2015**).** Due to a paucity of circulating influenza H3N2 and B cases, the study was not able to assess efficacy against these, but showed no efficacy against the circulating pandemic H1N1 influenza strain (-9.7%, 95% CI -62.6, 26.1). In Bangladesh, again, N = 1174 children were given Nasovac-S and N = 587 placebo. Overall vaccine efficacy to vaccine-matched strains was 57.5% (95% CI 43.6, 68.0), with efficacy against H1N1 and H3N2, 50.0% (95% CI 9.2, 72.5) and 60.4% (95% CI 44.8, 71.6) respectively (John C. Victor, PATH, personal communication). The reason for the discrepancy in these studies or the poorer performance of the H1N1 component is not clear and further emphasizes the need to undertake detailed immunogenicity studies of this vaccine in sub-Saharan Africa.

9 LAIV-induced immune responses

Although the exact correlates of protection from influenza illness following intranasal LAIV are not clear, existing data suggest that mucosal immune responses play a key role [7, 32-34]. Lower serum antibody titres are generated following LAIV when compared with inactivated influenza vaccination, despite the greater efficacy seen following LAIV in children [35, 36]. Several studies demonstrate the efficacy of LAIV in the absence of a robust serological response [32, 37, 38] and LAIV-induced nasal IgA responses were shown to protect from culture-confirmed influenza [7, 32]. The heterogeneity of sample collection techniques and IgA assay methodology make interpretation of many studies difficult. Robust and well-validated methods are therefore required for future exploration of LAIV-induced mucosal immunity. Potent cell mediated immunity is also elicited by LAIV but not inactivated influenza vaccines [35, 39, 40] and may also mediate protection from subsequent influenza [35].

10 Potential effects of the nasopharyngeal microbiome on LAIV immunogenicity

The reasons for geographical variation in vaccine responses are not fully understood and might have implications not only for influenza, but also for other mucosally delivered live vaccines. Emerging data from studies of the oral rotavirus vaccine, which show similar variation in protective efficacy, indicate that gut microbiome variability may explain the suboptimal responses observed in resource-limited countries [41, 42]. Interactions between gut microbiota, innate and adaptive immunity are recognized [43-46], but little is known about how changes in human nasopharyngeal microbiota might influence responses to vaccines delivered to the URT.

Murine data suggest that URT commensal bacteria can impact innate and adaptive immunity following intranasal wild-type influenza challenge in several ways [47-49]. Firstly, by providing

priming signals via Toll-like Receptors, often required for transcriptional activation of genes in the NLRP3-inflammasome-caspase-1 pathway [49]. Commensal bacteria also calibrate the activation thresholds of innate antiviral responses in macrophage interferon-signaling pathways [47]. These can impact on downstream virus-specific T-cell [47, 49] and nasal IgA responses [47], although the mechanisms for the latter are not clear. Possibilities include altered T-follicular helper cell help to germinal centre B-cells producing IgA *or* altered signaling from nasal epithelial cells leading to T-cell independent class-switch recombination.

Establishing whether similar relationships exist between nasopharyngeal microbiota and post-LAIV immunity in humans has the potential to explain both inter-individual response variability and why live mucosal vaccines perform poorly in resource-limited settings. It can also provide avenues to develop strategies for improving mucosal vaccine responses in the future via modulation of nasopharyngeal microbiota and/or circumventing these immunological defects via adjuvantation.

11 Potential effects of LAIV on nasopharyngeal microbiota

The human URT is a dynamic and complex environment, where communities of commensal and potentially pathogenic organisms co-exist. Bacterial and viruses are able to colonise this niche, dependent on interactions with other microbes and host factors. In a steady state, this dynamic process has no adverse consequences for the host and can provide beneficial effects influencing immune function [50], but imbalances can result in pathogenic species outgrowth and subsequent respiratory and invasive disease [51, 52]. Interactions between influenza and bacteria (E.g. *S. pneumoniae* and *S. aureus*) are well documented, resulting in secondary bacterial pneumonia [53, 54]. This is partly explained by an increase in nasopharyngeal bacterial carriage [55] due to epithelial barrier disruption [56, 57], increase in conditions favouring bacterial adhesion [58] and modulation of host innate immunity resulting in increased bacterial load [59-61].

The immediate effects of LAIV on the nasopharyngeal microbiome, however, are less clear. Although an attenuated virus, LAIV infects nasal epithelial cells and parallels some aspects of wild-type influenza host-pathogen interactions. Mouse models show that LAIV induces changes in nasopharyngeal microbiota, temporarily changing pneumococcal and staphylococcal bacterial burden in a similar way to wild-type influenza [62], possibly due to increased type I interferon, known to play a key role in enhanced bacterial colonization [55]. Whereas there are no safety concerns regarding LAIV leading to bacterial pneumonia given the hundreds of thousands of children who have received this vaccine, a temporary increase in bacterial burden could have implications on onward transmission in areas of high pneumococcal colonization, resulting in greater rates of pneumococcal colonization at a population level. Although underpowered, a recent study from a cohort of UK children given LAIV suggested similar observations may be seen in human studies [63]. In a setting like The Gambia where pneumococcal colonisation in children is extremely high [64], this interaction needs to be explored and results used to form the basis for more complex pneumococcal transmission studies if widespread LAIV use is considered.

12 Rationale

The WHO considers young children as a priority for influenza vaccination in developing countries [2]. In this context, data from high-income countries show that intranasal LAIV is more effective in children at preventing influenza than IIV [21, 65]. No robust data exist on the immunogenicity of LAIV in children from sub-Saharan Africa, where influenza infection in young children is common but no vaccine is currently in routine use. Limited data suggest that

the efficacy of intranasal LAIV may not be as robust in this setting as in higher resource settings where the majority of studies have been conducted.

Cutting edge bioinformatic methods used in systems biology now allow in depth exploration of the relationship between early innate gene expression and later adaptive responses to vaccines, highlighting previously unrecognised links between these two aspects of immunity. Professor Nakaya, a collaborator on this study, has used such techniques to identify novel associations between early expression of the kinase CaMKIV post-TIV and systemic anti-influenza antibody responses, as well as the B-cell growth factor *TNFRS17* and neutralizing antibody responses following Yellow Fever vaccination [66, 67]. To optimize responses in future generations of influenza vaccines, more detailed insights into the mechanisms associated with mucosal protection are required. Applying systems vaccinology methods to clinical studies of LAIV in African children would provide a means of dissecting the mechanisms by which better mucosal immunity may be elicited by intranasal vaccines in this setting.

13 Aims and Objectives

14 Study objectives

15 Primary

(i) To identify novel early systemic and mucosal molecular signatures following LAIV that are associated with subsequent robust nasal and oral influenza-specific immunoglobulin A (IgA) responses, in order to provide insight into the mechanisms of successful mucosal immunization.

(ii) To identify associations between nasopharyngeal microbiota and nasal and oral influenza-specific IgA responses post-LAIV in Gambian children, to explore whether microbiome variability can explain suboptimal immune responses in some individuals to live intranasal mucosal vaccines in Sub-Saharan Africa.

(iii) To establish whether LAIV impacts the nasopharyngeal microbiome, with a specific focus on the burden of *S. pneumoniae.*

16 Exploratory

(i) To identify novel early systemic and mucosal molecular signatures following LAIV that are associated with subsequent robust serum haemagglutination inhibition (HI) responses.

(ii) To identify novel early systemic and mucosal molecular signatures following LAIV that are associated with subsequent robust influenza-specific T-cell responses.

(iii) To describe the association between LAIV virus shedding at day 2 and/or 7 following immunization (as a measure of vaccine 'take') and influenza-specific mucosal IgA, serum HI and systemic influenza-specific T-cell responses to LAIV.

(iv) To explore the impact of antibiotic modulation of microbiota on LAIV-induced nasal gene expression and downstream effects on the mucosal immunogenicity of LAIV.

(v) To establish whether LAIV increases the burden of *S. aureus* in the nasopharynx.

(vi) To explore associations between nasopharyngeal microbiome profiles and post-LAIV nasal and systemic gene expression profile changes.

(vii) To explore whether the presence of respiratory viruses in the nasopharynx at the time of LAIV administration impacts on influenza-specific mucosal IgA, serum HI and systemic influenza-specific T-cell responses to LAIV

(viii) To establish whether an expansion in peripheral T-follicular helper (pTfh) cells occurs following LAIV.

(ix) To explore whether early systemic monocyte expansion following LAIV is associated with subsequent robust mucosal IgA responses.

(x) To explore whether an expansion of IgA-expressing B-cells with a mucosal-homing phenotype is detectable in blood following LAIV and whether this expansion correlates with the subsequent nasal and oral influenza-specific IgA response.

(xi) To explore how interferon-induced transmembrane protein-3 (IFITM3) host gene polymorphisms may impact on early innate and later influenza-specific immune responses induced by LAIV.

(xii) To explore the correlation between systemic HI and oral fluid immunoglobulin G (IgG) responses, to assess the suitability of the latter as a proxy measure of LAIV-mediated seroconversion obtained via non-invasive means.

(xiii) To explore any differences in early post-LAIV gene expression signatures and influenza-specific adaptive responses post-LAIV (nasal and oral IgA, serum HI or influenza-specific T-cell) in children who acquire laboratory-confirmed influenza following immunization and those who do not. Data will be obtained by passive surveillance and laboratory testing of nasopharyngeal swabs of children who go on to develop ILI as defined by:

- Sudden onset of fever of ≥38°C (axillary) AND
- either a cough or sore throat

17 Study endpoints

18 Primary endpoints

- Influenza-specific nasal IgA responses at day 21 post-LAIV
- Oral fluid IgA and IgG responses at day 21 post-LAIV
- Gene expression changes in nasal and systemic samples at day 2 following LAIV
- Nasopharyngeal microbiome profiles at baseline, day 7 and day 21 following LAIV
- Density of *S. pneumoniae* before and after LAIV as established by quantitative polymerase chain reaction (PCR)

19 Exploratory endpoints

- o Influenza-specific serum HI responses at day 21 post-LAIV
- o Influenza-specific peripheral T-cell responses at day 7 and 21 post-LAIV
- o Detectable vaccine virus (A/H3N2, A/pH1N1 and B) in nasopharyngeal samples obtained at day 2 and 7 post-LAIV, as determined by real-time reverse transcriptase polymerase chain reaction (rRT-PCR)
- o Respiratory viruses present in the nasopharynx at vaccination as determined by mulitplex rRT-PCR (e.g. parainfluenza, adenovirus, coronavirus, rhinovirus)
- o Nasopharyngeal microbiome profiles at baseline and at day 28 following a single dose of oral azithromycin in n = 30 individuals
- o Frequency of pTfh cells at day 7 and 21 following LAIV
- o Frequency of peripheral monocyte subsets at day 2 following LAIV
- o Frequency of B-cell subsets, IgA and mucosal homing marker expression at day 7 and 21 following LAIV
- o IFITM3-rs12252 genotyping (CC, CT and TT polymorphisms) by amplification and sequencing of genomic DNA
- o Detectable influenza (A/H3N2, A/pH1N1 and B) in nasopharyngeal samples obtained from children presenting with ILI during follow up period
- o Density of *S. aureus* before and after LAIV as established by quantitative polymerase chain reaction (PCR)

20 Study design

21 Type of study and design

All primary objectives and most exploratory objectives will be addressed through the conduct of a phase 4, randomized, controlled clinical vaccine trial. A total of 330 healthy children aged 24 – 59 months will be enrolled into one of three groups of equal size (3 x 110 children):

(i) LAIV-vaccinated (Group A - blood sample at day 0, 2, 21)
(ii) LAIV-vaccinated (Group B - blood sample at day 0, 7, 21)
(iii) Control group (Group C)

Allocation concealment will be maintained from the though the use of sealed envelopes. Block randomization stratified by sex will be used and a randomization sequence will be generated by an MRC Statistician not involved in the study. Investigators will not be blinded to group allocation from this point onwards as the sampling schedule differs between groups. Local laboratory staff will therefore not be blinded for this reason, but samples sent to external laboratories will be blinded (see section 3.2.3).

Two LAIV-vaccinated groups are included in order to achieve several exploratory objectives, which require blood sampling at different time points following vaccination, whilst minimizing the number of times children are bled times (i.e. 2nd blood sample at either day 3 or day 7 post-LAIV, but not both). The unvaccinated subjects will serve as a control group for the primary objective of assessing the impact of LAIV on the nasopharyngeal microbiome. Section 8 details the statistical considerations made to decide on the sample sizes to be recruited. The vaccination and sampling schedules for groups A, B and C are detailed in Tables 1.

Group C subjects will be administered LAIV after the NPS is taken on their day 21 visit, to offer them the same level of protection against influenza illness by participating in the study as those in groups A, B and D.

An additional 35 healthy children aged 24 – 59 months will be recruited in the 2nd phase of recruitment (Jan – June 2018) to assess the objective of how modulation of the nasopharyngeal microbiome with antibiotics impacts the mucosal immunogenicity to LAIV

(Group D – vaccination and sampling schedule in Table 2), by administration of a single dose of oral azithromycin 28 days prior to receiving LAIV. This timeframe has been chosen based on pilot data from the the Gambia showing significant alternation in the nasopharyngeal microbiome approximately 1 month after single dose oral azithromycin in Gambian children aged 1 – 7 years in mass trachoma treatment campaigns (S. Burr, unpublished, personal communication). These subjects will be matched as closely as possible by pre-antibiotic nasopharyngeal microbiome profile [1], age (24 – 35, 36 – 47, 48 – 59 months) and sex (in that hierarchical preference), in a nested case-control study, to n = 35 children recruited in the main clinical trial. This will be done after all children have been recruited and microbiome sequencing and analysis has been completed. To avoid allocation bias of these controls, an individual in the Bogaert laboratory blinded to the immunogenicity data will undertake this matching process. In brief, the microbiome profile matching will be performed by first categorizing which of eight described profiles each child's microbiome clusters with depending on the dominant taxa (e.g. *Staphylococcus, Streptococcus, Moraxella* etc.) as described previously by the Bogaert group [1].

The day 2 visit window for groups A, B and D is deliberately restrictive to allow both blood and NPS to be taken at a time when peak gene expression changes post-LAIV can be captured. The other visit windows are intentionally liberal to maximize the opportunity to obtain a full set of blood and NPS samples for the purpose of the analysis, whilst in each case, not compromising the scientific objectives they are designed to deliver. However, in each case, the intention will be to obtain the sample on or as close to the day specified as possible. The sampling schedule and associated laboratory assays to be performed on each clinical sample is detailed in Table 3. The procedure to deal with participants who default visit windows is discussed in section 5.4.

Participants in groups A and B (n = 110 each) will be enrolled for approximately 4 –10 months: 21 days of active participation during recruitment months January – June 2017 or January – June 2018, followed by attendance at the Sukuta Health Centre for assessment of any ILI that occurs up to the 31st of October in the year of recruitment into the study. This design has been chosen as data from Senegal show that peak influenza transmission occurs between July and October each year (John C. Victor, XVII International Symposium on Respiratory Viral Infections, Vancouver, CA, 2015).

Group C will be enrolled for 21 days, with participation finishing following the day 21 visit and receipt of LAIV (Table 1). Group D (n = 35) will be enrolled for approximately 4 – 10 months: 49 days of active participation during recruitment months January – June 2018, followed by attendance at the Sukuta Health Centre for assessment of any ILI that occurs up to 31.10.18. Those individuals presenting with possible ILI will have a limited history taken and if a sudden onset of a fever of ≥38°C (axillary) *and* either a cough or sore throat is present, a nasopharyngeal swab will be taken and diagnostic viral rRT-PCR performed.

The vaccine to be studied (Nasovac-S) has a WHO pre-qualification certificate and marketing authorization for the study population. However, the trial will provide detailed information on the determinants of immunogenicity and therefore inform the design of future generations of similar live intranasal influenza vaccines.

Table 1. Visit, vaccination and sampling schedule for groups A, B and C

Visit timing (from LAIV)	Visit window	Vaccinations and clinical samples	Group A (n = 110)	Group B (n = 110)	Group C (n = 110)
Day 0 visit	-	Intranasal LAIV	X	X	

		Oral swab	X	X	
		NPS	X	X	X
		Blood sample (5ml)	X	X	
Day 2 visit	+ 1 day	NPS	X	X	
		Blood sample (5ml)	X	-	
Day 7 visit	+7 days	NPS	X	X	X
		Blood sample (5ml)	-	X	
Day 21 visit	+7 days	Oral swab	X	X	
		NPS	X	X	X
		Blood sample (5ml)	X	X	
		Intranasal LAIV			X

LAIV - live attenuated influenza vaccine; NPS - nasopharyngeal swab

Table 2. Visit, vaccination and sampling schedule for group D

Visit timing	Visit window	Vaccination, antibiotics and clinical sampling	Group D (n = 35)
Day -28 visit	-	NPS	X
		Oral azithromycin	X
Day 0 visit	+7 days	Intranasal LAIV	X
		Oral swab	X
		NPS	X
		Blood sample (5ml)	X
Day 2 visit	+ 1 day	NPS	X
		Blood sample (5ml)	X
Day 7 visit	+7 days	NPS	X
Day 21 visit	+7 days	Oral swab	X
		NPS	X
		Blood sample (5ml)	X

LAIV - live attenuated influenza vaccine; NPS - nasopharyngeal swab

Table 3. Sampling schedule and use of clinical samples in laboratory assays

Visit timing from LAIV (window)	Clinical Sample	Laboratory assays
Day 0 visit	Oral swab	Influenza-specific IgA and IgG
	NPS^	Nasopharyngeal microbiome<br>Influenza-specific IgA<br>Host transcriptomic profiling<br>Viral multiplex RT-PCR
	Blood sample (5ml)	Innate cell profiling (FC)<br>Influenza-specific T-cell responses (FC)<br>B-cell phenotyping (FC)<br>Peripheral T-follicular helper cells (FC)<br>Host transcriptomic profiling<br>Serum haemagglutination inhibition
Day 2 visit<br>(+ 1 day)	NPS	Host transcriptomic profiling<br>LAIV virus shedding (RT-PCR)
	Blood sample (5ml)	Innate cell profiling (FC)<br>Host transcriptomic profiling

		Host DNA extraction
Day 7 visit (+ 7 days)	NPS^	Nasopharyngeal microbiome<br>LAIV virus shedding (RT-PCR)
	Blood sample (5ml)	Peripheral T-follicular helper cells (FC)<br>Influenza-specific T-cell responses (FC)<br>B-cell phenotyping (FC)
Day 21 visit (+ 7 days)	Oral swab	Influenza-specific IgA and IgG
	NPS^	Nasopharyngeal microbiome<br>Influenza-specific IgA
	Blood sample (5ml)	Peripheral T-follicular helper cells (FC)<br>Influenza-specific T-cell responses (FC)<br>B-cell phenotyping (FC)<br>Serum haemagglutination inhibition

LAIV - live attenuated influenza vaccine; NPS - nasopharyngeal swab; RT-PCR – reverse-transcriptase polymerase chain reaction; IgA – immunoglobulin A, IgG – immunoglobulin G; FC - Flow Cytometry
In addition, the nasopharyngeal swab from group D taken on the day -28 visit will be used for nasopharyngeal microbiome sequencing.
^NPS taken on days 0, 7 and 21 from group C will be used for nasopharyngeal microbiome sequencing

22 Allocation concealment, randomization and blinding procedures

23 Allocation concealment and randomization

Following informed consent, subjects will be assigned a unique subject identification number (section 3.2.2) and details recorded on an enrollment log. If eligibility is confirmed according to the defined inclusion and exclusion criteria (section 5.3), children will be randomized into one of the three equally sized groups as defined in section 3.1 above (for groups A, B and C).

Randomization will be undertaken using pre-prepared, sealed opaque envelopes to maintain allocation concealment. Block randomization stratified by sex will be undertaken using a computer generated randomization sequence pre-prepared by an individual not otherwise involved in the study or its analysis. The block size and randomization sequence will not be known by any member of the investigator team, hence ensuring allocation bias is avoided. Information regarding the group into which a participant has been randomized will be revealed to the team only when opening the envelope.

At the time of randomization, the person undertaking randomization will take the next envelope in numerical sequence. The randomization list will be cross-checked by a second individual both of whom will be required to sign to document the process.

The 35 subjects for group D will be recruited sequentially after recruitment in groups A, B and C have been completed, during the 2nd recruitment season (January – June 2018).

24 Participant identification

Once informed consent is provided, subjects will be allocated a unique subject identification number (and recorded on the screening log). The number will consist of the letters 'NAS' followed by 3 digits (e.g. NAS001). If eligibility is confirmed and the subject randomized, a suffix relating to the randomized group will be added to the identification number (e.g. NAS001A, NAS001B, NAS001C, NAS001D for groups A, B, C and D respectively). The subjects will subsequently be identified by this number alone on all further study related documentation and laboratory samples. This will facilitate accurate management of the different visit and sampling schedules, as well as laboratory assays, across the different groups.

25 Blinding of external laboratories undertaking analyses

Allocation concealment will be maintained as detailed above in section 3.2.1. Laboratory personnel processing samples and performing flow cytometry analyses on blood samples at the MRC Gambia will not be blinded to group allocation as sampling schedules vary across the groups (Tables 1 – 3). Laboratory personnel in external laboratories assessing the nasal and oral IgA, as well as serum HI, immunological endpoints will be blinded to group allocation, as well as whether samples are pre or post vaccine by allocation of a sample number. The code matching these sample numbers, to subject identification number and visit number will be held by investigators at the MRC Gambia.

26 Study Vaccine and medication

27 Nasovac-S

Nasovac-S is a trivalent live, attenuated influenza vaccine manufactured using the strains recommended by WHO for protection against seasonal influenza. The Nasovac-S to be used in this study will contain the 2016-17 and 2017-2018 WHO recommended northern hemisphere formulations, which consists of three viral strains from influenza A/H1N1, influenza A/H3N2 and an influenza B strain. The 2016-17 Nasovac-S will contain the following strains:

(i) A/17/California/7/2009/38 (H1N1)
(ii) A/17/Hong Kong/2014/8296 (H3N2)
(iii) B/Texas/02/2013-CDC-LV8B

SIIL will update the 2017-18 batch (if a change is required) once the WHO recommendations for that year are released. These strains are (a) **cold-adapted** (i.e., they can replicate at 25°C-33°C, a temperature that is restrictive for replication of many wild-type influenza viruses); (b) **temperature sensitive** (i.e., they cannot replicate at 40°C, a temperature at which many wild-type influenza viruses grow efficiently); and (c) **attenuated** (i.e., they may cause minor symptoms but do not produce classic influenza-like illness). The cumulative effect of the antigenic properties and the cold-adapted, temperature sensitive, and attenuated phenotypes is that the attenuated vaccine viruses replicate in the nasopharynx to induce protective immunity but do not cause influenza like illness or lower respiratory illness.

Within each of the LAIV's three types of reassortant viral strains, six RNA gene segments are derived from a MDV and are responsible for cold adapted, temperature sensitive, and attenuated phenotypes; and two RNA gene segments are derived from the antigenically relevant wild-type viruses recommended by WHO and encode the two surface immunogenic glycoproteins, HA and NA. Thus, the virus contained in Nasovac-S maintains the replication characteristics and phenotypic properties of the MDV and expresses the HA and NA of the current WHO recommended influenza vaccine strains.

28 Manufacturer

Nasovac-S is manufactured by the Serum Institute Of India Pvt. Ltd

29 Composition

Table 4. Composition of Reconstituted Nasovac-S:

INGREDIENT	CONCENTRATION
A/California/7/2009 (H1N1)	Not less than 10^7 EID_{50}/dose
A/Hong Kong/4801/2014 (H3N2)	Not less than 10^7 EID_{50}/dose
B/Brisbane/60/2008	Not less than $10^{6.5}$ EID_{50}/dose
Partially hydrolyzed gelatin	2.5%
Sorbitol	5.0%
L-Alanine	0.1%
L-Histidine	0.21%
Tricine	0.3%
L-Arginine hydrochloride	1.6%
Lactalbumin hydrolysate	0.35%
Phosphate buffer saline	Base

Nasovac-S is supplied as a vial containing a freeze-dried cake in a glass vial. Also supplied are an ampoule/vial containing sterile water (for inhalation as diluent), a syringe (for reconstitution), a syringe (for administration), a needle free device, and an intranasal spray device.

30 Therapeutic indications

Nasovac-S is indicated in individuals above 2 years of age for the active immunization for the prevention of influenza disease caused by two influenza A subtype viruses and one influenza type B virus expected to circulate in the season of vaccine manufacture.

31 Acquisition, storage, transportation and accountability

32 Acquisition

The study vaccine will be provided by SIIL and the investigators and their institutions will receive no monetary compensation from SIIL for their participation in this study. The investigator will be personally responsible for vaccine receipt and management or will designate a person who will be responsible for these activities at the MRC Gambia. SIIL will determine with the investigator or the person in charge at the site, the date(s) and time(s) of delivery of vaccine to the MRC Gambia.

The study vaccines will be supplied by SIIL in insulated shipping boxes at 2-8°C. Temperature will be monitored during the shipment. On receipt of the vaccines, the investigator or designee will acknowledge receipt of the vaccines and indicate shipment content, condition, and date. The condition of the packaging on arrival will be assessed and documented according to the applicable unit SOP. The number of vaccines received will be cross-checked with the shipping notification and documented. A reception record will be completed documenting the above and will be filed at the site, as well as being returned to SIIL to confirm receipt.

The investigator shall immediately inform the sponsor (MRC Gambia) and SIIL of any shipment temperature deviation and vaccines quarantined if necessary. The investigators will maintain an inventory/accountability record of study vaccine received. All study vaccines must be accounted for in the accountability record.

SIIL (through the shipping company) will also confirm that the cold chain was maintained during vaccine shipment by confirmation of continuous verification of temperature, freeze watch, and/or cold chain monitoring card. Temperature monitoring will be conducted and verified during transport between the vaccine manufacturer and the site.

33 Storage, transportation and handling temperature deviations

The vaccine will be stored according to vaccine storage requirements at the MRC Gambia, in access restricted refrigerators with a reliable back-up electricity generator and appropriate maintenance records as defined by Unit policy. Storage and transportation of vaccines will be continuously monitored with data logger devices to ensure a continuous temperature record is maintained. Vaccines should be stored and transported at 2-8°C until use. Vaccines will be transported to and from the field site in pre-prepared cold-boxes (e.g. Sofriboxes^{TM}) and temperature monitoring throughout the time the vaccines are in the field with the same data logger device. At the field site, the vaccines will be kept in the cool-boxes until reconstituted for administration. Although these will be opened and closed during these procedures, the time the boxes will remain open will be kept to a minimum. As the lyophilized vaccine is stable at standard room temperatures for 2 days, temporary increases in temperature from 2-8°C as boxes are opened and closed is not significant for vaccine stability. The temperature records for all the vaccines transported to and from the site on a given day will be downloaded and filed at the end of each day.

If there is any temperature deviation during transport/storage from SIIL to the study site or from the MRC Gambia to and from the vaccination site, it will be reported immediately to the sponsor according to the temperature monitoring SOP. SIIL state that the lyophilized vaccine is stable for a maximum of 2 days at room temperature. The lyophilized vaccine can also be frozen and is very stable at temperatures below -20 ^{o}C. However, the diluent provided should not be frozen. If any vaccine is kept at room temperature for 2 days or more in total (as recorded by the temperature data-loggers) or if the diluent is frozen, the vaccine pack should not be used in the study for vaccinating participants. As some vaccine stock will be used to develop controls for influenza rRT-PCR assays, these stock may still be used for laboratory *in vitro* assays.

34 Accountability

Strict vaccine accountability procedure will be maintained. The accountability process will ensure that vaccines given to each subject are traceable for monitoring purposes.

It is NOT permitted to use any of the supplies provided by SIIL for purposes other than those specified in this protocol (i.e. vaccinating participants enrolled in the study and *in vitro* assays). Once final accountability is completed, unused supplies will be destroyed at site. If required, destruction at the site will be carried out according to local biosafety standards for disposal of biological waste material after written approval from SIIL has been received.

35 Presentation, packaging and labelling

Study vaccine and diluent vials, along with the other vaccine supplies listed below, will be supplied by the manufacturer. For each participant, one carton will be provided containing the following:

1. Live attenuated influenza vaccine (single dose vial)
2. Diluent (0.5 ml) (single dose package)
3. Draw up needle
4. 1-ml Luerlock syringe
5. Intranasal spray device
6. Dose divider

36 Product storage and stability

All lyophilized study vaccines must be stored in a safe, locked, and secure place with no access by unauthorized personnel. Vaccines must be kept in the refrigerator (+2°C to +8°C) and must not be frozen. Study vaccine is packaged as a single-dose preparation, and is intended to be administered immediately following reconstitution. However, should the vaccine be reconstituted and not used immediately, it must be stored at +2°C to +8°C for no more than 6 hours after reconstitution after which it must be discarded.

Storage temperature should be monitored daily. Access to a backup refrigerator in case of power failure/breakdown is necessary. Vaccines that have been stored differently from the recommendations must not be used unless the sponsor provides written authorization for use. The lyophilized vaccine formulation is highly stable if stored according to manufacturer recommendations; however, the expiration date printed on the outer box must not be exceeded.

37 Dosage, preparation and administration

38 Dosage

A dose of 0.5 ml is administered as 0.25 ml per nostril using a syringe and a spray device while the subject is in an upright position with head tilted back slightly.

39 Preparation

Each vial of lyophilized vaccine must be reconstituted using 0.5 ml of sterile water that is supplied along with the vaccine.

40 Administration

The investigator, or designee, will be responsible for the preparation and administration of study vaccines to study participants according to the procedures in the study protocol and/or Study Specific Procedures (SSPs). All vaccines will be administered only by a study clinician or nurse who is qualified and trained to perform this function.

41 Contraindications and precautions

Nasovac-S is contraindicated in individuals with a history of hypersensitivity to eggs, egg proteins, gelatin, lactalbumin or other vaccine components listed in Table 5. Nasovac-S is also contraindicated in children receiving aspirin therapy, because of the association of Reye's syndrome with aspirin and wild-type influenza infection.

42 Safety data

Following a phase I of n = 40 adults which showed no safety concerns, in 2012, SIIL began a Phase II/III human trial of Nasovac-S. This study was conducted among 330 healthy children (aged 2 through 17 years, n=110), adults (n=110), and the elderly (n=110). Study vaccines included standard concentration LAIV (Formulation A) and a diluted LAIV comparator (Formulation B). In addition, the vaccine safety profile was favorable at both concentrations. There was no new onset of chronic medical conditions or SAEs identified among study participants, solicited adverse events were balanced between the groups, and 9 and 4 subjects in Formulations A and B respectively reported unsolicited AEs (SIIL Clinical Study Report, A Phase II/III clinical trial to assess safety and immune response of Trivalent, Live Attenuated Seasonal Influenza Vaccine of SIIL, 8 August 2012).

Out of 110 Pediatric subjects (≥ 2 years and <18 years) enrolled in the study, 46 children were ≤ 5 years, out of whom 24 received Formulation A (Group I) while 22 received Formulation B (Group II) (Table 5). Two children reported solicited local reactions. One child in Group I reported runny nose while another child from Group II reported stuffy nose. Both these events were of Grade I severity (mild), resolved without any sequelae within 2 days and didn't require concomitant medications. One child from Group II reported a solicited systemic reaction, cough, of Grade I severity (mild). It resolved without any sequelae, within one day and didn't require any concomitant medications. A total of ten unsolicited adverse events were reported from eight children. Unsolicited adverse events reported by children included abrasion on right forearm, abscess over left forearm, chickenpox, clean lacerated wound over chin, coryza, dog bite on right thumb, fever, loose motions and pain in abdomen. These were all judged to be unrelated to the vaccine. All of the unsolicited AEs resolved without sequelae within 1-9 days without need of concomitant medications. All except one of the AEs were Grade 1 (mild) in severity and no AE was reported as Grade 3 (severe). No death or SAE was reported in the study. None of the subjects experienced any hypersensitivity reaction. The safety data collected to date demonstrates that a single dose of both formulations of SIIL vaccine are safe and tolerable.

In June 2012, a Phase II safety and immunogenicity study was commenced in Bangladesh using Nasovac-S and the safety data are now published [31]. Of the 300 children included in the study and evaluated for safety and reactogenicity for the 42 day post-vaccination period, there were two unrelated SAEs (one fracture and one paracetamol overdose). There were no severe or life-threatening events. During the 7-day post-vaccination period, there were seven moderate solicited events and 102 mild solicited events (Table 5). During this same period there were 31 unsolicited events, of which 2 were moderate severity and the rest were mild (Table 6 shows unsolicited events across entire study). Episodes of protocol-defined wheezing (solicited) were not statistically different between LAIV and placebo groups during the 42-day period (n = 13 and n = 16 respectively, p = 0.697). These were all of mild severity. 15 of these events had positive laboratory tests for respiratory viruses at the time (five adenovirus, seven respiratory syncytial virus, three human metapneumovirus). All of these events resolved without sequelae.

Table 5. Solicited adverse event by vaccine group in first 7 days post-vaccination with Nasovac-S in Phase II safety and immunogenicity study in Bangladesh [31].

Solicited adverse event	LAIV group (n = 150) n (%)	Placebo group (n = 150) n (%)	*p*-value
Fever (measured 38C or higher)	5 (3.3)	5 (3.3)	1.00
Ear pain	2 (1.3)	2 (1.3)	1.00
Cough	10 (6.7)	8 (5.3)	0.81
Runny nose/nasal congestion	31 (20.7)	34 (22.7)	0.78
Sore throat	2 (1.3)	0 (0.0)	0.50
Headache	1 (0.7)	0 (0.0)	1.00
Irritability/decreased activity	0 (0.0)	0 (0.0)	-
Vomiting	2 (1.3)	3 (2.0)	1.00
Lethargy	0 (0.0)	0 (0.0)	-

All events were mild except for moderate events fever (2 LAIV vs. 1. placebo) and cough (3 LAIV vs. 1 placebo). There were no severe events.

Table 6. Participants with unsolicited adverse event by vaccine group over entire study period in Phase II safety and immunogenicity study of Nasovac-S in Bangladesh [31].

Adverse event classification	LAIV group (n = 150) n (%)	Placebo group (n = 150) n (%)	*p*-value
Diarrhoeal illness	38 (25.3)	32 (21.3)	0.50
Oral thrush	26 (17.3)	18 (12.0)	0.25
Boil/abscess/skin infection/ulcer/cyst	11 (7.3)	18 (12.0)	0.24
Helminthiasis	9 (6.0)	11 (7.3)	0.82
Scabies	8 (5.3)	6 (4.0)	0.79
Abdominal pain/distension	5 (3.3)	6 (4.0)	1.00
Allergy	6 (4.0)	6 (4.0)	1.00
Fungal infection	6 (4.0)	5 (3.3)	1.00
Others	55 (36.7)	51 (34.0)	0.72

A safety and efficacy study of Nasovac-S was recently completed in the Niakhar region, Senegal, recruiting children aged 24 – 71 months in a randomized, double-blind, placebo-controlled design (John C. Victor, XVII International Symposium on Respiratory Viral Infections, Vancouver, CA, 2015). N = 1174 Nasovac-S vaccinated and n = 587 placebo-vaccinated (2:1) children were included. Local reactogenicity (cough, sore throat, runny nose, nasal congestion, ear pain) up to day 7 following vaccination was similar between the groups: with 20.6% and 19.9% of the LAIV and placebo group reporting mild symptoms, 0.4% and 0.2% reporting moderate symptoms and no subjects reporting severe symptoms. Systemic reactogenicity (chills, headache, vomiting, irritability/decreased activity, muscle/joint pain, documented fever, tachypnea) in the same timeframe was rare with 2.7% and 3.2% reporting mild symptoms in LAIV and placebo groups, 0.4% and 0.7% reporting moderate symptoms and 0.1% and 0.0% reporting severe symptoms respectively.

Of the unsolicited adverse events documented as occurring at greater than 1% frequency, 2.3% reported gastrointestinal disturbance (diarrhea, abdominal pain, vomiting, gastroenteritis, enteritis), with these equally balanced between the two groups and no severe symptoms noted. 1.9% were noted to have respiratory, thoracic or mediastinal events (bronchiolitis, nasopharyngitis, oropharyngeal pain, pneumonia, pulmonary congestion, runny nose), with more in the placebo arm (2.7%) than LAIV (1.4%). No life threatening events were noted. Protocol defined wheezing occurred in only one subject (0.2%) in the placebo arm at day 0 – 42 following vaccination and in four children in both the LAIV (0.3%) and placebo (0.7%) groups between day 43 to six months following vaccination.

These data combined provide robust safety data on Nasovac-S in children aged 2 years and over.

43 Azithromycin

Azithromycin is a macrolide antibiotic widely used in the treatment of community acquired infections in children and adults due to organisms such as *Haemophilus influenzae, Moraxella Catarrhalis, Streptococcus pneumoniae, Streptococcus pyogenes, Chlamydophilia pneumoniae* and *Mycoplasma pneumoniae.* It has US FDA approval to treat acute otitis media in children ≥6 months, pharyngitis/tonsillitis in children ≥2 years and community acquired pneumonia in children ≥6 months.

Single-dose azithromycin has also been widely used in mass trachoma eradication campaigns, including those in The Gambia.

44 Manufacturer

Azithromycin (Zithromax®) for oral solution is produced by Pfizer corporation.

45 Composition

Zithromax® for oral suspension is supplied in bottles containing azithromycin dehydrate powder equivalent to 300mg, 600mg, 900mg, or 1200mg per bottle and the following inactive ingredients: sucrose; tribasic anhydrous sodium phosphate; hydroxypropyl cellulose; xanthan gum; FD&C Red #40; and flavoring including spray dried artificial cherry, crème de vanilla, and banana. After constitution, a 5mL suspension contains 100mg or 200mg of azithromycin.

46 Acquisition, transportation and accountability

Zithromax® will be procured via MRC pharmacy procurement procedures for medications from Pfizer corporation or authorized distributors and shipped according to manufacturer's

recommendations for clinical use. Once final accountability is completed, unused supplies will be donated to the MRC Unit The Gambia Clinical Services.

47 Product storage and stability

Zithromax® dry power is stable at temperatures below 30°C. As room temperature in The Gambia can exceed this, Zithromax ® dry powder will be stored refrigerated at 2 - 8°C in MRC pharmacy stores prior to use. Reconstituted suspension is stable below 30°C for 10 days. Reconstituted suspension will also be stored refrigerated at 2 - 8°C in MRC biobank and transported to and from the field site in a cool box prior to use. A date of reconstitution and date of expiry (i.e. 10 days from reconstitution) will be noted on the bottle and those that are not used the date of expiry will be discarded.

48 Dosage and administration

Azithromycin will be administered as a single dose, in oral suspension form for children. Dosing will follow the WHO recommendations for treatment of active trachoma, which is a single dose of 20mg/kg in children (up to the maximum adult dose of 1g).

49 Adverse reactions and precautions

Azithromycin is well tolerated, particularly in the single dose regimens recommended for mass trachoma eradication campaigns by the WHO [68]. The most common side effects of azithromycin are diarrhoea, nausea, abdominal pain and vomiting, each of which may occur in fewer than one in twenty persons who receive single dose azithromycin. These are typically short-lived, lasting less than 24 hours [68]. Allergic reactions are rare.

50 Selection and withdrawal of participants

51 Selection of participants

The study will randomize 330 children between 24 and 59 months, followed by sequential recruitment of a further 35 children within the same age range (see section 3.1), which allows for approximately 10% participant attrition during the study and required sample sizes to achieve primary objectives (Section 8). Screening of potentially eligible children will continue until this number or the recruitment end date (30.06.2018) is reached. Recruitment will take place over two six month periods, January – June 2017 and January – June 2018 (first subject first visit, FSFV to last subject first visit, LSFV of each season). These months have been chosen to coincide with periods when wild-type influenza infections are likely to be at their lowest, therefore least likelihood of interference with study endpoints. The recruitment target for season 1 (January – June 2017) will be 195 children (65:65:65 into groups A, B and C) and season 2 (January – June 2018) 170 children (45:45:45 into groups A, B and C, followed by 35 into group D – see section 3.1). This represents 29 – 33 children per month or approximately 7 - 8 children per week. Any shortfall in numbers caused by lack of recruitment or withdrawal in season 1 will be made up in season 2 by increasing target numbers.

52 Identification of potential study subjects

Healthy 24 – 59 month old children will be recruited via two main strategies: community sensitization will take place and parents encouraged to attend with their children to the Health Centre, where recruitment will take place. Older siblings of children attending the Health Centre for their expanded programme of immunization (EPI) schedule will also be recruited. Clinical trial staff stationed in this clinic will identify potential subjects in conjunction with the public health officers coordinating the clinic.

The parent/guardian will be approached by clinical trial staff to provide information and discuss participation. Under these circumstances, an opportunity will always be offered for a parent/guardian to discuss the study with other members of the family at home. Contact details for the parent/guardian will be obtained and stored in a sensitization log, to allow field staff to follow potential subjects up, and a further appointment will also be arranged at which recruitment may take place. Informed consent from parents or guardians will be taken prior to any study procedures being undertaken.

53 Eligibility of participants

Children must meet all of the inclusion criteria and none of the exclusion criteria to be eligible to participate in the trial.

54 Inclusion criteria

Each subject being initiated on the study protocol must satisfy the following inclusion criteria at study entry:

- Healthy male or female child at least 24 months of age and less than 60 months of age at the time of study entry.
- Resident in the study area and with no plans to travel outside the study area during the period of subject participation.
- Informed consent for the study participation obtained from a parent (or guardian only if neither parent is alive or if guardianship has been legally transferred (see section 11.2).
- Willingness and capacity to comply with the study protocol as judged by a member of the clinical trial team.

55 Exclusion criteria

No subject being initiated on the study protocol may have any of the following exclusion criteria at study entry:

- Serious, active, medical condition, including but not limited to:
  - chronic disease of any body system
  - severe protein-energy malnutrition (weight-for-height Z-score of less than -3)
  - known genetic disorders, such as Down’s syndrome or other cytogenetic disorder
- Active wheezing
- History of documented hypersensitivity to eggs or other components of the vaccine (including gelatin, sorbitol, lactalbumin and chicken protein), or with life-threatening reactions to previous influenza vaccinations.
- History of documented hypersensitivity to macrolide antibiotics
- History of Guillain-Barré syndrome.
- Receipt of aspirin therapy or aspirin-containing therapy within the two weeks before planned study vaccination.
- Any suspected or confirmed congenital or acquired state of immune deficiency including but not limited to primary immunodeficiencies including thymus

disorders, HIV/AIDS, hematological or lymphoid malignancies (blood tests will not be routinely undertaken with this regard as part of the study).

- Any current immunosuppressive/immunomodulatory treatment or receipt of any such treatment within the six months preceding trial enrolment (for corticosteroids this is defined as a dose of prednisolone (or equivalent) of greater than 2mg/kg/day for one week or 1mg/kg/day for one month. The use of topical corticosteroids is not an exclusion criterion.

- The use of inhaled corticosteroids within the last one month.

- Receipt of an influenza vaccine within the past 12 months.
- Has any condition determined by investigator as likely to interfere with evaluation of the vaccine or be a significant potential health risk to the child or make it unlikely that the child would complete the study.

- Any significant signs or symptoms of an acute illness or infection including:

    - an axillary temperature of 38.0°C or above or documented fever of 38°C or above in the preceding 14 days.

    - Any acute respiratory infection within 14 days of enrollment visit.

If the reason for ineligibility is likely to be temporary (e.g. a fever of 38°C or above or acute respiratory infection) and either will or may resolve before the infant reaches 60 months, they will not be recorded as a screening failure but instead will be re-screened within an appropriate future time-window (e.g. at least 14 days after the last documented fever of 38°C or above or resolution of respiratory illness) and a decision made regarding eligibility at that point.

If eligibility is confirmed, subjects will be recorded in an enrollment log.

Those that are not eligible based on one or more exclusion factors will be noted as screening failures on the screening log, with reasons for exclusion documented.

56 The role of laboratory investigations in confirming eligibility

No laboratory analysis will be routinely undertaken in the process of assessing a child's eligibility. Abnormal findings during history or examination may result in an infant being referred for further investigations or treatment outside the clinical trial itself according to local clinical practice. This would not, as a matter of course, preclude subsequent inclusion in the trial if an infant was confirmed to be eligible.

57 Withdrawal and discontinuation of subjects

If after signing of the informed consent form, the investigator ascertains that the subject does not fulfill all criteria for study entry, the case report form (CRF) pages need not be completed. Only the reason for subject exclusion should be noted on the screening log. Data on screening failures will therefore be collected.

If a subject who does not fulfill all criteria for study entry is inadvertently deamed eligible and included in the trial (either randomized in the case of Groups A, B and C or given azithromycin in group D), the investigator must terminate the subject's participation in the trial. If the subject

was randomized and administered the study vaccine, the subject should be followed for safety for 21 days following administration of Nasovac-S, if possible. However, no further samples will be taken from the subject and the data will be excluded from the analysis.

A parent/guardian may withdraw a child from the study at any time without giving a reason. This will not in any way affect their care otherwise. A parent/guardian may either withdraw from further participation only (in which case all clinical samples and data obtained prior to withdrawal will be evaluated as part of the study) or withdraw from further participation *and* from the use of any clinical data/samples already collected. If the latter option is requested by the parent/guardian of the child, collected samples will be destroyed, a request made for data withdrawal and this process documented. Irrespectively, the clinical trial team will make all reasonable efforts to monitor, follow-up and when necessary treat adverse events under such circumstances according to the procedures outlined for this trial (see section 7).

58 Missed visit windows and criteria for withdrawal

Subjects that miss visit windows will continue on the study protocol and all procedures relevant to the *subsequent* visit undertaken as planned at the correct time. Samples for the missed visit window will *not* be collected. Samples that have already been collected will still be used for inclusion in analyses addressing relevant objectives.

In the event that subjects receive unplanned antibiotic therapy after commencing the study but prior to their planned final visit (i.e. the day 21 visit), the subjects will be excluded from analyses of primary objectives relating to nasopharyngeal microbiome changes. Samples collected will still be included in analyses relating to other objectives.

59 Study procedures and evaluation

60 Study schedule

A summary of the study schedule for each group is detailed in Appendix 1.

61 Sensitization

Sensitizing the parents/guardians of potential study subjects will precede the formal recruitment period to ensure that parents with children of the necessary ages are aware of the trial as far in advance as is practical.

62 Community sensitization

A series of meetings with key leaders within the local community including the Alkalo (village/community chief), representatives of women’s and family groups, Muslim and Christian religious leaders and other community advocates will be undertaken at venues within the local community, generally identified by members of the community itself and also at the clinical trials site. During these meetings, details regarding the trial will be discussed and a chance given for questions to be asked to ensure everybody present gains a full understanding of the central purpose of the trial and other key information (e.g. risks and benefits) as outlined in the informed consent document. Following these meetings information regarding the trial is disseminated throughout the local community through well-established community networks. These meetings aim to ensure that all parents/guardians of potentially eligible children become aware of the trial and are thus encouraged to attend the Sukuta Health Centre, where recruitment will take place. The meetings also serve a critical role in

ensuring influential members of the community properly understand the trial and therefore that accurate information is passed on and misinformation is avoided.

63 Individual sensitization

Members of the clinical trial team will also base themselves within the clinic where the routine EPI schedule is administered at the Sukuta Health Centre, in order to identify parents/guardians who have older children potentially eligible for the study. A member of the clinical trial team will discuss the study with parents/guardians who are provisionally interested and agree to receive additional information. The discussion will be based on the contents of the informed consent document (ICD) and a copy of the ICD will be provided to the parent/guardian at the end of the discussion.

Parents/guardians with potentially eligible children who are interested will be encouraged to discuss the study with their spouse and other family members as appropriate. Following individual sensitization of the parent/guardian, a member of the field team may provide information to other family members (particularly the spouse) either by telephone or through a visit to the subject's residence according to the preference of the mother. This ensures that a lack of English literacy does not preclude anybody the parent/guardian feels is important from gaining a proper understanding of the proposed trial (the ICD is written in English only – see 6.1.2).

64 Informed consent

The exceptional circumstances under which a guardian rather than a parent can provide the consent are described in section 11.2. Parents/guardians will be allowed at least 24 hours from receipt of study information and sensitization prior to children being recruited into the study.

65 Provision of information – English literate parents or guardians of children

In the case of parents/guardians who are literate in English, they will be provided with a copy of the ICD but a member of the clinical trial team will nonetheless review the contents of the ICD line by line in English to ensure all details are covered and that the subject has the opportunity to ask questions regarding any aspect of the trial.

Prior to undertaking the informed consent process, the member of the clinical trial team undertaking consent will confirm and document English literacy by asking the subject to read a section of the ICD and explain the meaning briefly. If there is any doubt regarding the adequacy of English literacy, consent will be undertaken in a local language in the presence of an impartial witness (see 6.1.2.2). Of note, if consent is undertaken in English and without an impartial witness, the whole process must be completed in English; additional use of a local language must not occur under these circumstances.

66 Provision of information – non-English literate parents or guardians of children

The ICD regarding the trial will be written in English only. Although many subjects speak only local languages (e.g. Mandinka, Wolof and Fula), these languages are primarily spoken rather than written languages and extensive efforts to translate and back-translate ICD into local languages have proved to be unsuccessful. Thus, consistent procedures have been developed locally and are well established for the provision of trial related information to non-English literate subjects.

In the case of potential participants who are not literate in English but only in one of the local languages, a member of the clinical trial team fluent in the local language (as well as being English literate) will review the ICD line by line translating directly from the English ICD to the appropriate local language. In this case an impartial witness must be present throughout all elements of the consent process and must attest that that the information on the ICD has been given accurately and in full. The impartial witness will also be required to sign the ICD to confirm that this has been the case (Section 6.1.2.4).

Impartial witnesses are not part of the clinical trial team and are not employed by the MRC. They are generally recommended through the local community and may, for example, be retired teachers or other professionals. A potential participant may, if preferred, bring their own impartial witness, for example and English literate relative. Procedures related to consent and the role of the impartial witness are provided in detail in the relevant MRC Unit The Gambia policy document.

67 Assessment of understanding

Having reviewed the ICD line by line (section 6.1.2.1 and section 6.1.2.2) the parent/guardian of potential participants will be asked a standardized series of questions to assess their understanding of key information regarding the trial. The questions are asked by a different member of the clinical trial team from the individual undertaking the consent process.

A subject who fails to attain a pre-determined standard of correct answers will have all the information in the ICD reviewed with them again and the questions re-attempted. Failure to reach the required standard on a second occasion precludes enrolment.

68 Documentation of informed consent

Informed consent for study participation will be documented on the signature page of the ICD. The option to consent for the future use of any residual samples on study completion will also be given on the same page but the provision of such additional consent is not required for participation in the main trial.

English literate participants will be required to sign the ICD as well as writing the time and date that they completed the ICD signature page.

Participants who are not English literate may thumb-print the ICD (although some participants who are not literate in English may still prefer to sign or mark the ICD both of which are also acceptable). In any of these cases, the impartial witness will complete the time and date portion of the ICD to confirm the time and date the thumbprint/signature/mark was provided. The impartial witness is then required to sign, time and date the ICD to attest to the fact that the information in the ICD has been provided to the participant in full, that an opportunity to ask questions has been provided, that the questions have been answered to the participant's satisfaction and that consent appears to be given freely. Finally, the person taking consent signs, dates and times the ICD.

A certified copy of the ICD is provide to the participant unless it is refused for some reason in which case it will be filed at the trial site. Such refusal must be documented in the subject's file so it is clear that the subject has been offered a copy. The original ICD is also filed at the trial site.

69 Confirmation of ongoing willingness to participate

Written consent is obtained only at the first visit. However, an opportunity to ask further questions will be given to each visit and ongoing willingness to participate will be confirmed

verbally and documented. The presentation of a subject to the clinical trial site will be taken to represent the ongoing willingness to participate on the part of the parent/guardian in the event that another responsible carer brings the subject for a particular visit.

70 Screening and enrolment

71 History

A focused history based around inclusion and exclusion criteria will be obtained and documented in a structured CRF to establish eligibility for enrolment (see section 5.3).

72 Medical and drug history

A medical history will be obtained by the investigator, sub-investigator or study nurse. Any abnormal findings requiring further treatment will be managed according to local practice or an appropriate referral made according to the nature of the condition. A lack of exclusion criteria will be confirmed.

All concomitant medications a child is receiving will be recorded in the CRF. Data on antibiotic use within the last month will also be collected and recorded. The use of concomitant medication, other than those considered exclusion criteria, does not preclude recruitment (see section 5.3.2).

73 Clinical examination, vital sign and anthropometry

A physical examination is performed by the investigator or sub-investigator with particular focus on the inclusion and exclusion criteria set out for the study. All relevant positive and negative findings will be documented within the subject's clinical notes and key findings will be captured in the CRF. Any abnormal finding requiring further treatment will be managed according to local practice or an appropriate referral made according to the nature of the finding.

A subject's axillary temperature will be recorded at each visit, using a calibrated thermometer. At the same time points the respiratory rate and heart rates will be measured.

Height and weight will be measured and recorded at each visit using appropriately calibrated equipment. Calculate Z-score (a z-score of less than -3 indicated severe protein-energy malnutrition and is an exclusion criterion).

74 Inclusion and exclusion criteria checklist

Eligibility against a defined inclusion and exclusion criteria checklist will be confirmed prior to final enrolment and documented in the CRF.

75 Final confirmation of eligibility and definition of enrolment

Eligibility for enrolment will be confirmed on the basis of the above (Section 6.1.3). A child will only be defined as being enrolled in the trial once final confirmation of eligibility has been determined and randomization has occurred.

76 Randomization

Randomization will be undertaken using pre-prepared sealed envelopes as described in section 3.2.1.

77 Clinical samples

78 Blood sample

Blood samples will be collected by trained personnel who have had their competency confirmed and documented according to the written procedures defined for the trial. See section 6.3.1.

79 Nasopharyngeal swab

Nasopharyngeal swabs will be collected by trained personnel who have had their competency confirmed and documented according to the written procedures defined for the trial. See 6.3.2.

80 Oral swab

Oral swabs will be collected by trained personnel who have had their competency confirmed and documented according to the written procedures defined for the trial. See section 6.3.3.

81 Vaccination of children and immediate post-vaccination safety follow up

All vaccinations will be administered by trained personnel who have had their competency confirmed and documented according to the written procedures defined for the trial. Written procedures describing the administrations of Nasovac-S will be used to ensure consistency of administration following appropriate training (see section 4.1.7.3).

All personnel administering Nasovac-S will be trained to recognize and manage anaphylaxis as appropriate in the clinical setting and no vaccines will be administered in the absence of the necessary resuscitation equipment as set out in the relevant written procedure.

82 Immediate post-vaccination observations

Following vaccination with Nasovac-S, all children will be observed at the clinical trial site for 30 minutes. Vital signs (temperature, heart rate, respiratory rate) will be taken at the end of this period and all subjects will be assessed for local and systemic reactogenicity to confirm they are in stable a condition to go home. This period of observation may be extended if necessary based on the initial clinical assessment or vital signs.

83 Unscheduled visits with possible influenza-like illness

All subjects in the study will be eligible for medical care at the clinical trial site from study entry until the 31st of October during the year of recruitment (i.e. at least 4 months, taking into account final date of recruitment during each period will be the 30th of June). Parents/guardians will be encouraged to attend with children in the study especially if they develop symptoms consistent with an influenza-like illness (symptoms of fever, cough or sore throat).

Clinical trial staff will complete a basic evaluation of symptoms and subjects who have received Nasovac-S as part of the study and fulfil the case definition below will have a NPS taken:

- Sudden onset of fever of ≥38 ºC (axillary) and
- Either a cough or sore throat

All subjects will be provided with medical care on site or referred on as appropriate according to clinical needs.

84 Procedures for obtaining clinical samples

All clinical samples will be obtained at the clinical trial site only.

85 Blood samples

5.0ml blood samples will be obtained from subjects at the time-points specified in Tables 1 and 3. Blood samples will be obtained by peripheral venepuncture from children by a doctor or nurse from the clinical field team following appropriate training and confirmation of competency against relevant study specific procedures governing this process. Blood will be collected into appropriate serum separation tubes, anti-coagulated blood tubes for cellular immunology assays and Paxgene® tubes for human transcriptomic studies respectively, depending on the visit (See Table 3). If blood volumes are limited, priority will be given to Paxgene® tubes, anti-coagulated blood tubes and serum separation tubes in that order.

86 Obtaining nasopharyngeal samples

A single paediatric Copan flocked nasopharyngeal swab will be obtained from children at the time points specified in the schedule of events. Detailed practical instructions regarding the collection of NPS will be outlined in the relevant study specific procedure.

Once obtained, NPS will immediately be placed aseptically into a vial of RNAprotect® cell reagent, the swab snapped as per manufacturer's recommendations and the lid of the vial closed immediately.

87 Obtaining oral swabs

A saliva sample collected using an Oracol swab will be obtained from children at the time points specified in the schedule of events. Detailed practical instructions regarding the collection of Oracol swabs will be outlined in the relevant study specific procedure.

Once obtained, the swab will immediately be placed in the collection tube according to the manufacturer's instructions.

88 Additional clinical samples

Any additional blood or other samples (e.g. urine) required for the routine clinical assessment and care of an infant may be obtained as indicated with the verbal consent of the parent/guardian.

89 Sample transport

Samples will be transported from the Health Centre field site to the main MRC site laboratories at Fajara as follows:

90 Blood samples

Blood samples will be transported in cool boxes although must not be frozen. Samples must reach laboratory within 3 hours allowing serum separation to take place within 4 hours. Non-serum samples must reach the laboratory within the same timeframe. All samples will be processed in the research immunology laboratory according to study specific procedures.

91 Nasopharyngeal swabs

NPS vials will be placed on an ice block and transported to the laboratory within 3 hours for processing and storage within 4 hours. Samples will be processed in the research immunology laboratory according to study specific procedures, with the 1ml sample being split into five equal aliquots of 200ul and stored at -70 ºC.

92 Oral swabs

Oral swabs will be placed in a cool box and transported to the laboratory within 3 hours for processing and storage within 4 hours. Samples will be processed in the research immunology laboratory according to study specific procedures. Samples will be diluted 1:2 in buffer according to protocols developed at Public Health England Laboratories and stored at -70 ºC until further use.

93 Endpoint evaluations

94 Laboratory endpoints

95 Nasopharyngeal microbiome sequencing

Microbiome sequencing will be performed via collaboration with Prof. Debby Bogaert (University of Edinburgh, UK and University Medical Centre, Utrecht). NPS samples will be shipped frozen on dry ice to either the University of Edinburgh or Utrecht Medical Centre Bogaert laboratory (to be confirmed). DNA will be extracted from NPS collected material using phenol/bead beating in combination with the AGOWA Mag mini DNA extraction kit. Microbiota will be studied by Illumina Miseq sequencing of the 16S small subunit ribosomal DNA gene according to previously described methods by the Bogaert group [69]. After DNA isolation, the V4 region of the 16S-rDNA gene will be amplified by qPCR, fragments pooled and the library sequenced. Specific PCR for *S. pneumoniae* and *S. aureus* may be performed to validate any longitudinal changes in these species suggested by 16s fragment analysis.

96 Peripheral blood and nasopharyngeal transcriptomic studies

RNA will be extracted from whole blood in Paxgene® tubes and NPS collected material according to protocols optimized in the Ghazal laboratory (University of Edinburgh) and Bogaert laboratory respectively. Extracted RNA will be shipped frozen on dry ice to the University of Edinburgh and microarrays performed on the Affymetrix® platform. Key signatures identified will be validated further by gene-specific RT-PCR in the first instance.

97 Influenza-specific IgA and IgG

An aliquot of NPS collected material and oral fluid will be shipped frozen on dry ice to RIVM laboratories in the Netherlands, where anti-influenza IgA will be measured using a protein microarray [70]. This fluorescence-based assay utilizes very small amounts of material and preliminary data shows very good correlation with nasal fluid anti-influenza IgA estimated by more traditional indirect enzyme-linked immunosorbent assay (ELISA) methods (T. de Silva,

A. Meijer, unpublished). In addition, indirect ELISA assays to determine anti-influenza IgA and IgG from oral fluid, where more material is available will be set up and performed at the MRC Gambia immunology research laboratory. Total IgA and IgG will also be estimated in NPS material and oral fluid (by ELISA) and influenza-specific IgA and IgG expressed as a ratio of total IgA and IgG respectively. Further exploratory assays may be carried out after optimization to obtain a more functional output of anti-influenza activity in mucosal secretions.

98 Serum haemagglutination inhibition assays

Serum aliquots will be shipped frozen on dry ice to Public Health England laboratories, Colindale, UK and/or The University of Edinburgh and serum antibody titres measured against the three influenza strains included in the vaccine.

99 Flow cytometry assays

Fresh whole anti-coagulated blood will be used for optimized exploratory flow cytometry assays at several times points to assess innate cell phenotyping, influenza-specific T-cell responses, B-cell phenotyping and peripheral T-follicular helper cells following LAIV (Table 3). Influenza-specific T-cell responses will be assessed by intra-cellular cytokine staining following stimulation with overlapping peptide sets matching conserved internal proteins (matrix and nucleoprotein) and HA of the influenza A vaccine strains. All these assays will be performed at the MRC Unit The Gambia research immunology laboratory.

100 Viral RNA detection

An aliquot of NPS collected material will be used to extract viral RNA at the MRC Unit The Gambia molecular laboratories. The presence of several respiratory viruses at vaccination (e.g. parainfluenza, adenovirus, coronavirus, rhinovirus) will be evaluated by viral multiplex rRT-PCR. Shedding of LAIV strains (pdmH1N1, H3N2 and influenza B) will be assessed at day 2 and 7 post-LAIV (Table 3).

101 IFITM3 gene polymorphisms

Genomic DNA will be extracted from an aliquot of whole blood (Table 3). DNA will be shipped frozen on dry ice to Prof. Dong’s laboratory in Oxford, who will perform genotyping of the IFITM3 rs12252 gene of all vaccinated subjects (n = 255) in the study for exploratory analyses to see whether polymorphisms in IFITM3-rs12252 impact on immunogenicity of LAIV. IFITM3 CC genotype is associated with greater expression of interferon-stimulated genes in wild-type pdmH1N1 infections and the 1000 genome project suggests a relatively high prevalence of this genotype in the Gambia at 21% (Tao Dong, personal communication). It is possible, therefore, that different innate immune responses to LAIV due to IFITM3 polymorphisms may account for differences in downstream adaptive immune responses.

102 Safety considerations

103 Definitions

104 Adverse event

An adverse event (AE) is any untoward medical occurrence in a child enrolled in the study which does not necessarily have a causal relationship with the either the vaccine or drug administered or study participation. An adverse event therefore includes any unfavorable and unintended sign (including an abnormal laboratory finding), symptom or disease occurring

during the study.

Any AE which occurs after an immunization may be termed and AE following immunization (AEFI).

105 Serious adverse event

An AE will be considered serious (i.e. an SAE) if:

- It results in death
- Is life-threatening[1]
- Requires inpatient hospitalization
- Results in persistent or significant disability/incapacity

[1] This refers to an event when the child was at risk of death at the time of the event; it does not refer to an event which hypothetically might have caused death if it were more severe

The start date for an SAE is defined as the day the relevant SAE criteria (as above) was met and not the day an AE which subsequently developed into an SAE began. The end date for an SAE is the day the criteria rendering the SAE serious resolves, i.e. the end of the hospitalization period or the time the SAE was no longer considered life-threatening. In these cases the related AE may be ongoing after the end of the SAE (e.g. if a child has ongoing pneumonia but is discharged from hospital). 'Death' and 'Persistent or significant disability/incapacity' will be defined as ongoing and will be closed for the purposes of further reporting once relevant clinical details have been captured (e.g. cause of death) and, in the case of disability/incapacity, the condition is considered stable in the short term.

All SAEs occurring throughout the study must be reviewed and evaluated by a study clinician (SAE relationship to study vaccine must be evaluated as outlined in Section 7.1.4) and recorded on an SAE form and reported. All such SAEs should also be followed until satisfactory resolution or until the investigator deems the event to be chronic or the patient to be stable.

106 Assessment of severity

All AE will be graded for severity using the terms mild, moderate and severe. The event itself, however, may be of relatively minor medical significance (such as a severe headache). This is not the same as 'serious', which is based on the outcome or action required (see section 7.1.2 above).

The following terms are used to define severity:

Mild (Grade 1): Mild signs or symptoms which appear to be no more than a minor irritation. Symptoms do not require therapy or a medical evaluation; signs and symptoms are transient. Little or no change in normal activities (e.g. feeding, sleeping, behavior etc); minimal parent/guardian concern.

Moderate (Grade 2): Events introduce some inconvenience or concern to the parent/guardian. Usually improved by simple therapeutic measures (e.g. paracetamol). Moderate change in normal activities (e.g. feeding, sleeping, behavior etc); moderate parent/guardian concern.

Severe (Grade 3): Events significantly alter the child's normal daily activities (feeding, sleeping, behavior, etc) and generally require systemic drug therapy or other treatment; high parent/guardian concern.

The following specific AEs will be graded according to the following parameters:

Measured fever will be graded as follows:

1. Mild: measured temperature from 38.0°C to 38.4°C axillary
2. Moderate: measured temperature from 38.5°C to 39.0°C axillary
3. Severe: measured temperature >39.0°C axillary

Tachypnea will be graded as follows:

1. Mild: measured respiratory rate 31 to 40 breaths/min
2. Moderate: measured respiratory rate 41 to 50 breaths/min
3. Severe: measured respiratory rate 51 breaths/min or greater

7.1.4 Assessment of relatedness

The clinician's assessment of an AE's relationship to the vaccine or drug is part of the documentation process, but it is not a factor in determining what is or is not recorded in the study. If there is any doubt as to whether a clinical observation is an AE, the event should be recorded. All AEs must have their relationship to study vaccines assessed using the terms: related or unrelated. To help assess, the following guidelines will used:

- Related – The event is temporally related to the administration of the study vaccine or drug ***and*** no other etiology explains the event.
- Not Related – The event is temporally independent of study vaccine or drug and/or the event appears to be explained by another etiology.

Solicited local and systemic AEs occurring in the week following vaccination with Nasovac-S will, in most cases, be considered to be related to the study vaccine in the absence of a clear alternative diagnosis (e.g. malaria, lower respiratory tract infection). Details of expected AEs in the Summary of Product Characteristics will be used to aid this decision.

Any AE which is judged to be related to the study vaccine will be defined as a suspected adverse reaction (SAR).

Any SAE which is judged to be related to the study vaccine but unexpected will be defined as a suspected unexpected serious adverse reaction (SUSAR).

107 Methods and timing for assessing, recording, and analyzing safety parameters

As the safety profiles of both interventions (Nasovac-S and azithromycin) are well established and the study does not have defined safety endpoints, data on adverse events will be collected by passive follow up and questions asked during scheduled visits to the clinical trial site.

108 Time windows for the collection of adverse event data

All adverse events that occur through day 21 post-vaccination with Nasovac-S in groups A, B and D will be assessed and recorded (during scheduled visits at day 2, 7 and 21 after vaccination or if subjects present to the clinical trial site due to illness in between these visits). All adverse events occurring through Day 28 following administration of azithromycin (Group

D only) will be assessed and recorded (at the scheduled visit at day 28 after azithromycin or if subjects present to the clinical trial site due to illness before this visit). Parents/guardians will be advised to contact the study team via telephone or attend the clinical trial site if their child is unwell in between scheduled study visits.

Group C will receive Nasovac-S after NPS has been taken on the day 21 visit, so they are also afforded the same potential benefit of influenza vaccination by participating in the study as groups A, B and D. Given the existing safety data on Nasovac-S (Section 4.1.9), no extra visits will be scheduled for group C specifically for the purpose of gathering adverse event data. Parents/guardians will, however, be advised to contact the study team or bring their child to the health centre if they have any concerns and adverse event data collected, graded and investigated as per AEs in other groups detailed in this section. This will be particularly relevant for post-vaccine AEs for 7 days following vaccination, but aligns with the agreement to assess all study subjects if they are unwell until October 31st of the year of vaccination.

109 Solicited safety data

All participants will be assessed for local and systemic reactions through 7 days post-vaccination with Nasovac-S. Using a standardized data collection instrument, the following local and systemic reactions will be documented and be graded on magnitude of reaction on the scheduled day 2 visit (for dya 0 – 2 symptoms) and day 7 visit (for day 3 – 7 symptoms) following vaccination (see section 7.1.3).

Local reactions:

- Cough
- Nasal discomfort
- Runny nose
- Sore throat
- Stuffy nose
- Ear pain

Systemic Reactions:

- Chills
- Headache
- Vomiting
- Fever (measured)
- Irritability/Decreased Activity
- Muscle/Joint pain
- Tachypnea (measured respiratory rate)

110 Response to solicited local and systemic reactogenicity in children

Any local or systemic reactogenicity (grade 1 or above) will be reviewed at the day 2 or 7 post-vaccine visits or as judged to be clinically indicated in the event a participants parent contacts the study team in the interim.

Any solicited local or systemic reactogenicity ongoing at day 7 will be documented as an adverse event and followed up according to clinical need. The frequency of follow-up in this case will be determined by the nature of the adverse event by a research clinician.

Any grade 3 local or systemic reactogenicity or a child with a fever (≥38℃) will be reviewed by a study clinician the same day or within 24 hours at the Sukuta Health Centre.

111 Unsolicited safety data collection

All AE will be classified according to their seriousness (section 7.1.2), severity (section 7.1.3) and relatedness to vaccination or azithromycin (section 7.1.4).

112 Investigation of unsolicited adverse events

Additional investigations undertaken if a child is unwell will be based on clinical judgment. However, any child with a fever of >38.5℃ beyond days 1 to 7 following vaccination will have a blood culture, a NPS (for respiratory virus diagnostic tests) and a malaria rapid diagnostic test performed.

113 Verbal autopsies

Following any maternal or infant death a verbal autopsy will be conducted using the latest WHO verbal autopsy form or applicable locally modified form. Autopsies are not otherwise performed in The Gambia and would not be considered to be culturally acceptable.

114 Recording and coding of safety data including concomitant medication

Prior to data entry and for the purposes of safety reporting all unsolicited AE will be classified using the Medical Dictionary for Regulatory Activities (MedDRA) (Version 18.0 or subsequent updates) according to System, Organ, Class (SOC), Preferred Term (PT) and Lowest Level Term (LLT).

Concomitant medication use will be documented using generic drug names and standardized administration routes and frequencies.

All data coded in this way will also have the AE/medication indicated in a free-text field for subsequent manual validation of the coded data.

115 Safety reporting procedures

SAE/IME will be reported by the PI to the sponsor and the local safety monitor within 24 hours of the investigator becoming aware. The report should be submitted on the designated SAE reporting form for the trial. The reports will be submitted by e-mail. Follow-up reports will subsequently be submitted as new information becomes available until the SAE/IME is closed or defined as ongoing (Section 7.1.2).

SAE/IME that are judged to be related to the study product but unexpected (SUSAR) will be reported by letter by the PI to The Gambia Government/MRC Joint Ethics Committee within 15 calendar days or within 7 calendar days if the event is fatal or life threatening. All SAE/IME will be reported by the PI to the Gambia Government/MRC joint ethics committee in the annual report

All SAE/IME will be reported to the Republic of the Gambia Medicines Control Agency by the sponsor according to the latest requirements of the agency.

All deaths occurring in the trial, irrespective of whether judged to be related, will be reported by the investigator by letter to the Gambia Government/MRC Joint Ethics Committee at their next meeting.

If new information becomes available that may alter the safety or conduct of the trial the investigator will inform the sponsor, the Local Safety Monitor, The Gambia Government/MRC Joint Ethics Committee and The Republic of the Gambia Medicines Control Agency in writing as soon as they become aware and generally within 5 calendar days.

SAE/IME that are judged to be related to the study product and unexpected (SUSAR) will be reported to SIIL by the sponsor via email, working closely with the PI.

All fatal or life threatening SUSAR will be reported to the Medicines Control Agency as soon as possible and within 7 calendar days of the sponsor becoming aware of the case. Other SUSAR will be reported as soon as possible and within 15 calendar days of the sponsor becoming aware of the case.

116 Safety database

All safety data required for pharmacovigilance will be captured in the clinical trial database developed for the trial (section 9.3). A separate pharmacovigilance database will not be used.

117 Pause rules

As there is robust safety data on the study vaccine Nasovac-S, as well as azithromycin, this study does not have any defined safety endpoints. However, the occurrence of any SUSAR will always result in a pause in recruitment. An evaluation will be made on whether the risk-benefit balance of the study has changed in light of the SUSAR. The decision on whether to continue the trial will be made by the sponsor, taking into account the information provided by the PI, the local safety monitor, the manufacturer (SIIL) and if necessary external experts (e.g. pharmacists). The sponsor's representative will collect all data and consult with MRC (the sponsor) head office prior to a decision being made.

118 Study discontinuation

Study discontinuation could occur following the assessment of the conditions related to a given pause rule being activated such that ongoing recruitment was no longer judged to be appropriate for any reason (see section 7.7). Also, if further information, external to the trial, becomes available (e.g. though newly published literature) which is considered to alter the safety profile of the trial such that it is no longer justified.

119 Statistical considerations

120 Sample size

Sample size calculations have been considered with relation to the three main primary objectives in the study (section 2.1.1).

121 Identifying novel early systemic and mucosal signatures following LAIV that are associated with robust nasal and oral influenza-specific IgA responses

A precise power calculation is difficult to perform as a number of markers will be studied in an unbiased analysis, rather than testing whether a single defined marker is associated with vaccine endpoints. Microarrays will be performed (peripheral blood and NPS-derived RNA) on a subset of children at the opposite ends of the IgA response spectrum following LAIV (n = 30 in each group), thus identifying those at the extremes of response. A further 30 children, given azithromycin, will also have microarrays performed at baseline and day 2 following LAIV and results compared to n = 30 age, sex and baseline microbiome profile-matched children.

Helder Nakaya has successfully identified markers of vaccine immunogenicity in cohorts of

similar or smaller sizes than those proposed following several vaccines including LAIV, IIV, yellow fever and polysaccharide-based meningococcal vaccines [66, 67, 71]. Over 2000 genes were differentially expressed at day 3 post-LAIV when compared to baseline [66].

Although there are no data in Gambian children given LAIV to use in sample size calculations, estimates can be provided based on microarray data from healthy Gambian children (2 – 59 months) recruited as controls in a pneumonia study (James Jafali, unpublished data), which is the best available data to use. The *ssize* package [72] in R was used to estimate the number of children required to identify differentially expressed genes between two groups. Using the strict Bonferroni correction to adjust for multiple testing, 30 subjects in each group is required to ensure that at least 90% of genes (of 49,386) have a power of ≥ 90% at alpha 0.01 to detect a 2-fold change (see Figure 1 below). Using the more relaxed false discovery rate to control for multiple testing, 10 subjects in each group would be sufficient.

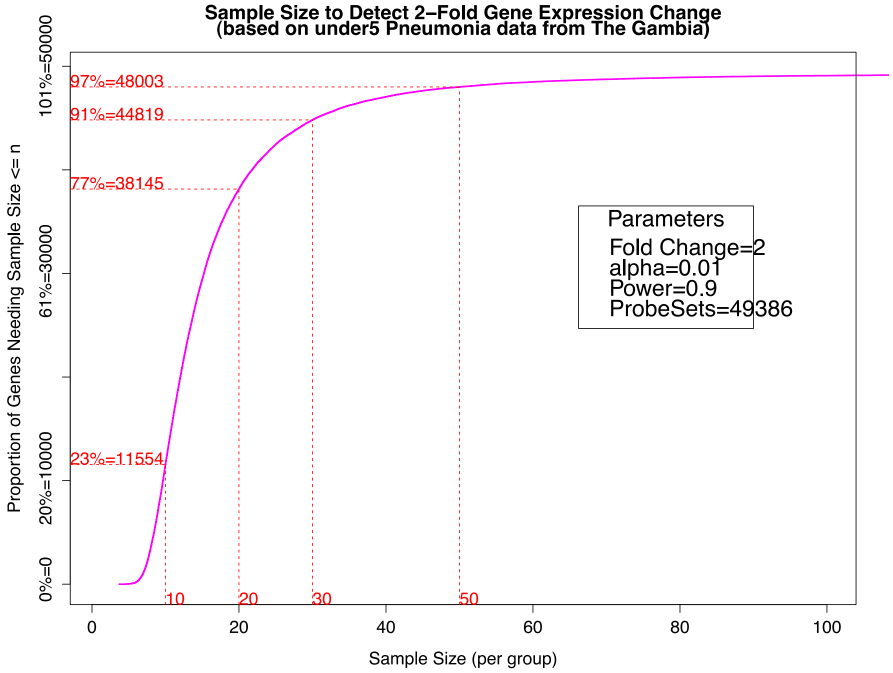

Figure 1. Sample size estimates to detect 2-fold gene expression changes

122 Identifying associations between nasopharyngeal microbiota and nasal and oral influenza-specific IgA responses post-LAIV in Gambian children, to explore whether microbiome variability can explain suboptimal immune responses in some individuals

Calculations were performed using the Shiny application for determining sample size calculations for case-control microbiome studies (https://fedematt.shinyapps.io/shinyMB/) [73]. Simulations were run using the anterior nares-derived microbiome data available on the application, estimating that analyses would consider the most abundant 150 operational taxanomic units (OTUs). IgA responders and non-responders were defined as cases and controls respectively and sample size/power calculations performed to detect a 1.5-fold

difference in abundance in the 10 most abundant OTUs and 2-fold difference in abundance in the next 10 most abundant OTUs, between the two groups (Figure 2).

Power calculations were performed using these parameters, for a range of sample sizes from 50 – 150 per group and using the Wilcoxon-Mann-Whitney (WMW) test applied to each OTU individually and the resulting *P*-values multiplicity adjusted with the Bejamini and Hochberg method to control the false discovery rate ('WMWavg' – Figure 3). Prior data suggest that approximately 50% of children receiving LAIV are IgA responders, using a definition of ≥2-fold rise in IgA from baseline [7]. At an alpha of 0.05, 82 subjects per group would give a power of 80%. A sample size of n = 100 per group was selected (i.e. n = 200 children given LAIV, accounting for approximately 50% showing an IgA response), which even accounting for some drop outs would give a power of ≥80%.

If required, the complexity of data may be reduced by defining microbiota composition according to 8 distinct profiles described by the Bogaert group [1] rather than variability across 20 different OTUs/species, thus reducing the need for correction for multiple testing and increasing the power significantly.

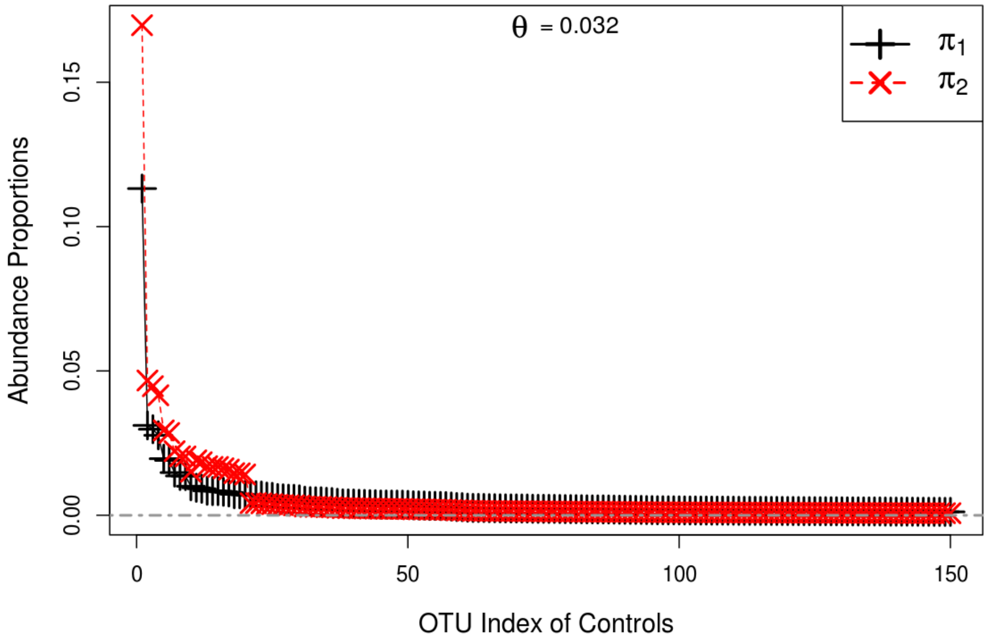

Figure 2. Simulations of anterior nares derived microbiome data showing differences between cases and controls of the 20 most abundant OTUs.

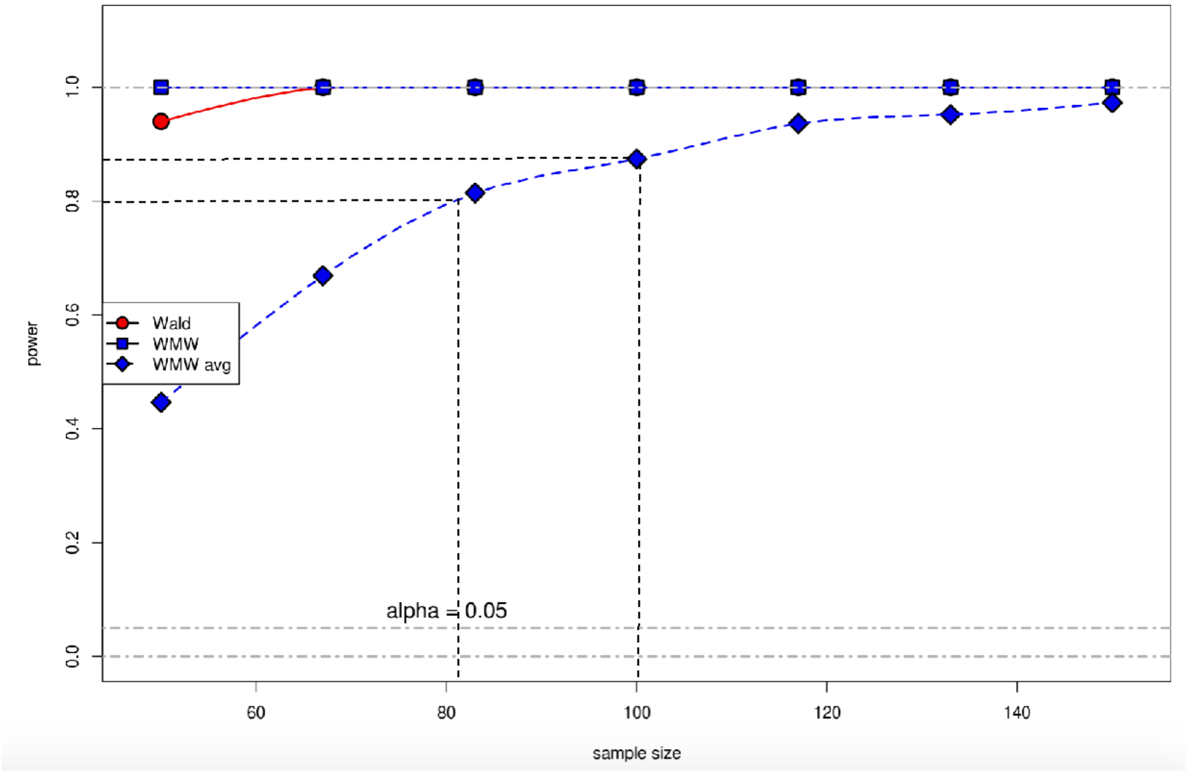

Figure 3. Sample size and power estimates to detect differences in the top 20 most abundant OTUs between two groups (1.5-fold difference in the top 10 abundant OTUs and 2-fold difference in the next 10 most abundant OTUs). WMWavg refers to the Wilcoxon-Mann-Whitney test as applied to each OTU and multiplicity adjusted to control for false discovery rate.

123 Establishing whether LAIV impacts the nasopharyngeal microbiome, with a specific focus on the burden of *Streptococcus pneumoniae*

The complex issue of sample size requirements to assess bacterial carriage dynamics in humans following LAIV has received in depth scrutiny in the literature [74] and needs to take into account likely colonization rates in the study population. Sample size calculations have been estimated in the literature using a stochastic-stimulation approach, with variance in carriage density parameterized by data previously collected in the authors' laboratory. Taking into account a likely *S. pneumoniae* carriage prevalence of 80% in the study population [64], a sample size of n = 100 vaccinated children would provide ≥90% power at an alpha of 0.05, to detect a ≥2-fold increase in carriage density of *S. pneumoniae* from baseline following LAIV [74]. At a carriage rate of 90%, a sample size of n = 230 (the number of children planned to be given LAIV) would provide ≥80% power at an alpha of 0.05, to detect a 1.5-fold increase in carriage density of *S. pneumoniae.* It has been reported that 1.5 to 2-fold increases in carriage density of *S. pneumoniae* may represent clinically relevant values with respect to a potential for increased transmission of *S. pneumoniae* [74, 75].

124 Planned analyses

125 Microarray data analysis:

These analyses will be conducted via collaboration with the Ghazal group (University of Edinburgh), facilitated by collaboration with Helder Nakaya (University of Sao Paulo) and the MRC statistics and bioinformatics team.

Analyses will be conducted using Perl and Python scripts, R/bioconductor packages, as well as a broad range of tools and databases. Similar methods will be applied to both nasal and blood transcriptome data.

Microarray data will initially be normalized to background values and expression fold change calculated for each gene by comparison of day 2 with baseline values. Non-informative genes will be filtered out at a defined threshold of expression from baseline (e.g. 1.25-fold seen in at least 20% of vaccines [66]). Several independent statistical tests will be performed and only those significant in all tests taken forward for further analysis. Comparison of normalized data between groups (e.g. those with and without a ≥ 2-fold increase in influenza-specific nasal IgA [7]) will utilize linear modeling of the $\log_2$ scale expression values between groups and subsequent empirical Bayesian statistical testing with vertical p-value adjustment for multiple testing to control for false discovery rate. Pearson's correlation will be used to investigate relationships between fold-expression changes in genes and the magnitude of antibody or T-cell responses to identify probe sets that correlate with an immunological response [66].

Computational network-based approaches such as ingenuity pathway analyses will be used to examine relationships in the data using correlation of gene expression and biological relationships. Several groups have compiled compendia of coordinately expressed modules of genes and can be visualized as nodes linked by connections to other genes with correlated expression level. As such, vaccine responses can be analyzed basis of a number of co-regulated genes rather than individual expressed genes.

Minimum sets of genes capable of predicting an immunological response will be identified via supervised learning classification methods such as Discriminant Analysis via Mixed Integer Programming (DAMIP) [66] and supervised vector machine learning techniques. RT-PCR of identified genes will be used for validation of these findings. Initial analysis will be conducted on phase I collected samples and this dataset used as a training set to identify predictive genes. The predictive accuracy of these classification rules will be tested using phase II collected samples.

Deconvolution meta-analysis of gene expression will be performed on blood transcriptional responses to reveal immune cell type related to the identified sets of genes. Similar analyses will be attempted on nasal transcriptomic data, although this will rely on the availability of *in vitro* epithelial cell microarray data. In addition, the cell types picked up in nasal samples will also be less heterogeneous than in whole blood, with the majority of RNA likely to be from epithelial cells and very little from monocytes, dendritic cells, B and T-lymphocytes.

126 Microbiome sequencing:

These analyses will be performed via collaboration with the Bogaert group (University of Edinburgh), under consultation with the Antonio group at the MRC Gambia.

Quality checks will be performed on initial sequencing results to filter reads and fragments of poor quality. Sequences of similar identity will be clustered together to produce OTUs. The relative abundance of each OTU will be calculated. Representative sequences from each OTU will be compared with a reference database to identify genera (and species if sequence

resolution allows). Absolute abundance will also be calculated by combination of relative abundance data with 16s qPCR data.

127 Association between microbiome and mucosal response to LAIV

Several approaches will be taken to explore association between the nasopharyngeal microbiome and response to LAIV. Firstly, a supervised analysis will be performed to compare the relative abundance of the top 20 OTUs between mucosal IgA responders and non-responders, using appropriate parametric or non-parametric analyses (e.g. ANOVA, Kruskal-Wallis test) and *post*-hoc tests to adjust for multiple comparisons.

Secondly, an unsupervised analysis will be performed. Baseline data will be analyzed through Qiime, allowing statistical analyses [76, 77], clustering of data and/or individuals through Unifrac methodology and bacterial network analyses through CoNet analyses (http://systemsbiology. vub.ac.be/conet). This methodology allows differentiation of several microbiome 'clusters' or 'profiles', charazterized by a particular biomarker species (e.g. *Streptococcus* spp., *Staphylococcus* spp., *Haemophilus* spp. *Dolosigranulum/Corynebacterium* spp. combined profile) [77]. The proportion of IgA responders and non-responders within each cluster or profile can then be calculated.

Finally, univariate and multivariate linear regression models will be used to incorporate microbiome and other factors as predictors of an IgA response.

128 Impact of LAIV on the nasopharyngeal microbiome

Initial sequence data analysis will be performed as described above. Univariate and multivariate linear regression regression models will be used to study the effect of vaccination with LAIV on microbiota profiles at day 7 and 21 post-vaccination, adjusting for factors such as antibiotic use within the last 1 month, age and nutritional status in all multivariate analyses. Relative effect sizes and their 95% confidence intervals will be calculated for all significant OTUs. Inter-individual variability between vaccinated and unvaccinated children at day 7 and 21 will be calculated using Pearson correlations and tested for significance using the Mann-Whitney U test.

The relative and absolute abundance of the OTUs representing *S. pneumoniae* and *S. aureus* will be compared at day 7 and 21 following vaccination with baseline data and validated via qPCR. Differences in *S. pneumoniae* and *S. aureus* density at day 7 and 21 post-LAIV will be estimated using a regression model, including a fixed effect for the vaccine group and random effects for other relevant factors.

129 Other statistical analyses:

Chi-squared test will be used to compare baseline characteristics (e.g. nutritional status, recent antibiotic use) between groups such as IgA responders vs. non-responders or LAIV-vaccinated vs. unvaccinated. Regression modeling will be utilized to explore relationships between exploratory outputs post-LAIV (monocyte function, nasal soluble factors, pTfh cells) and vaccine endpoints (HI titre, nasal IgA, T-cell responses). For all analyses, model assumptions would be checked to confirm reliable estimates and all relevant co-variates would be taken into account.

130 Further exploratory analyses:

Integration of complex datasets such as the transcriptomic and microbiome data, alongside immunological data such as influenza-specific responses at baseline and post-LAIV will

require exploratory approaches that are not well established at present, although methods are emerging [78, 79]. Exploratory analyses will be done in conjunction with bioinformaticians in the Ghazal and Bogaert groups, as well as the MRC statistics and bioinformatics team.

131 Data management

Full details regarding the handling of trial data will be described in the trial data management plan (DMP) which will be consistent with appropriate data management SOP of MRC Unit The Gambia and applicable regulatory requirements. All documents used during the trial will be version controlled and dated.

132 Source data

Source will be defined for all data to be collected during the trial in a source data designation log. Paper CRF (pCRF) will act as the source for certain data, which will include reactogenicity record forms with data collected during scheduled visits to the clinical trial site. Certified photocopies of relevant source documents will be made for subsequent source data verification (SDV) purposes.

133 Data collection

Subject data will be collected or transcribed contemporaneously on pCRF according to written CRF completion guidelines for the trial. The pCRF will be developed with the input of sponsor, investigator and data management personnel. Draft CRF will be piloted by the investigator site and will be reviewed to ensure all protocol required data for the assessment of the trial endpoints are collected and also that the data required to confirm protocol compliance are captured.

All pCRF will be printed with an individual tracking number allowing their unique identification and providing a link between data in the database and the completed pCRF. At the time of completion, following randomization, the subject indentification number of the child will be written on each pCRF. In addition, each pCRF will include the date of the visit and, when relevant, the time that particular activities were undertaken (e.g. to confirm the time interval between vaccination and the recording of the post-vaccination vital signs). Each pCRF will also include a unique identifier documenting the member of the clinical trial staff who completed it. Such information will allow the designation of clinical trial staff completing certain CRF to be confirmed within the database.

The pCRF will be printed in duplicate on copy paper which will be separated on completion following a QC process (section 9.4). The original will be submitted for data entry while the copy will remain at the site with the investigator. A subject folder will be maintained for all children and will contain the pCRF and other documents including the informed consent document and copies of relevant source data for ease of review within the investigator team and also by the study monitor.

134 Clinical database

An OpenClinicaTM clinical database management system (CDMS) will be used for the trial. The CDMS will be developed based on an annotated copy of the final CRF in which the type of data to be entered into each field will be specified. Free text field will be limited and, when possible, coding will be undertaken by the investigator team at the time data is first captured (e.g. adverse events and concomitant medications).

Computer system validation and database testing, including user acceptance testing (UAT) using dummy data generated by the field team, will be undertaken in a test environment and documented prior to trial initiation. Details of the testing to be undertaken will be outlined in the DMP. Data will be 100% double entered from pCRF. Discrepancies will be resolved

contemporaneously by the second member of the data entry team. Missing, illegible or invalid data in a given field in the pCRF will generate a data query for resolution by the investigator site.

135 Data quality checks, cleaning and validation

Completed pCRF will be quality checked (QC) for completeness and consistency and accuracy prior to the separation of the copy. Any errors detected at this point will be rectified immediately by the investigator site and will be initialed, dated and explained (if necessary) using standard GCP correction ensuring that a trail is maintained and the initial entry is not obscured. Once separated, no further changes will be made to the original or copy pCRF itself (section 9.3) and a data query will be raised (see below).

Data entry errors will be minimized through a process of double data entry and verification (section 9.3).

Following data entry a process of data validation will be undertaken to identify inconsistencies (e.g. dates, times, visit chronology); incorrect and/or implausible data (e.g. birth weight outside plausible limits); protocol deviations (e.g. subject eligibility - age calculated from date of birth; visit time-windows); also to identify missing visit data (e.g. absence of expected follow-up visits and follow-up samples). Checks will include those run at the time of data entry and those run in batches, for example, on a weekly basis (i.e. batch validation). Additional manual checks of data listings (e.g. for AE and concomitant medication coding) will also be undertaken as specified in the DMP. Any data validation checks which 'fire' will be reviewed by the trial data manager and, unless related to data entry will prompt a data query to the investigator site using a Data Clarification Form (DCF). An audit trail will be maintained to ensure any changes made in the database are also reflected with the original and copy of the pCRF (through the attachment of the completed DCF). Data validation checks which fire as a result of data entry errors will be resolved by the data manager.

Completion of data cleaning and validation procedures will be documented using a defined check-list governing these procedures which will be signed by the sponsor and investigator prior to data-lock. The procedure to confirm the completion of database cleaning and describing database lock, including those governing changes of access and edit control will be defined in the DMP.

136 Laboratory data

Laboratory data generated at both the main MRC site and also in the laboratories of external collaborators will be produced in a format required for subsequent data analysis without the need for additional data transcription as defined in the trial data management plan.

137 Data archiving

138 Paper records

All clinical trial documents will be archived within the access controlled data archive facility at the MRC Unit for a minimum of 15 years following the end of the trial following SOP at The Unit covering these procedures. Contents of the Investigator site file and associated subject records will remain the responsibility of the PI or documented designee throughout this period. The sponsor trial master file (TMF) and associated documents will be archived separately and will remain the responsibility of the sponsor. Access to all archived material will be controlled and will require written permission of the investigator or sponsor as applicable.

139 Electronic data

All electronic data and metadata generated within the trial will be archived according to the relevant MRC Unit SOP covering this process. Reliable back-up will be maintained and processes will be in place to ensure the confidentiality and security/authenticity of the data archived. Ongoing accessibility of archived data will be assured (i.e. if a storage system or format becomes obsolete) as outlined in the applicable MRC Unit SOP.

140 Confidentiality

The Principal Investigators will maintain appropriate medical and research records for this study in compliance with the principles of good clinical practice and regulatory and institutional requirements for the protection of confidentiality of participants. The study team members will have access to records.

The authorized representatives of the sponsor, the ethics committee(s) or regulatory bodies may inspect all documents and records required to be maintained by the investigator, including but not limited to, medical records for the children in this study. The clinical study site will permit access to such records.

141 Protocol deviations

A protocol deviation (PD) is any noncompliance with the clinical trial protocol, good clinical practice (GCP), or other applicable regulatory requirements. The noncompliance may be either on the part of the participant or the investigator including the study team members, and may result in significant added risk to the study participant. As a result of a deviation, corrective actions will be developed and implemented promptly.

If a deviation from, or a change of, the protocol is implemented to eliminate an immediate hazard(s) to trial participant without prior ethics approval, the PI or designee will submit the implemented deviation or change, the reasons for it, and, if appropriate, the proposed protocol amendment(s) as soon as possible to the sponsor (MRC Unit The Gambia) for agreement and to the Gambia Government/MRC Joint Ethics Committee s committee for review and approval.

The PI or designee will document and explain any deviation from the approved protocol on the CRF, where appropriate, and record and explain any deviation in a protocol deviation form that will be maintained as an essential document.

142 Quality control and quality assurance

143 Study monitoring

Study monitoring will be undertaken to verify that the rights and well-being of children enrolled in the trial are protected, that the data reported are accurate, complete and verifiable from source documents and that the trial is conducted in compliance with the current trial protocol, with ICH-GCP guidelines and with applicable regulatory requirements.

Full details of the monitoring to be undertaken prior to, during and at the end of the trial will be documented in the risk-based trial monitoring plan which be agreed and signed off by the sponsor prior to trial initiation. Monitoring will include a site initiation visit which will be undertaken prior to the first subject consent, on-site monitoring visits throughout the trial and a close out visit.

144 Quality control of laboratory data

A laboratory analytical plan based on the clinical trial protocol will be written prior the trial initiation which will provide the details of all laboratory procedures to be undertaken during the trial and the quality systems to be followed.

145 Microbiome data

DNA extraction from NPS and microbiome sequencing will be undertaken at the University Medical Centre Utrecht and/or the University of Edinburgh, according to already established internal standard operating procedures and Quality Assurance (QA) processes. These have been optimized for DNA extraction and microbiome sequencing from low microbial abundance sites such as the human nasopharynx, including minimization of contamination.

146 Microarray data

Microarray assays will be undertaken at University of Edinburgh and designated laboratories, according to already established internal standard operating procedures and QA processes.

147 Influenza-specific IgA (protein microarray)

Protein microarray assays will be undertaken at RIVM, The Netherlands, according to already established internal standard operating procedures and QA processes.

148 Influenza-specific and total IgA and IgG (ELISA)

Standard operating procedures for ELISAs to measure influenza-specific and total IgA and IgG from nasal/oral fluid will be established at the MRC Gambia immunology research laboratories. These will be based on well-establised and QC'd procedures currently being used in line with Good Clinical Laboratory Practice (GCLP) at the mucosal immunology laboratory at Imperial College London. QC and QA procedures will be established for local protocols.

149 Haemagglutination inhibition assays

Serum HI assays will be performed at Public Health England Laboratories, Colindale or the University of Edinburgh, according to already established internal standard operating procedures and QA processes.

150 Viral polymerase chain reaction (PCR) data

rRT-PCR assays to detect both influenza (for LAIV shedding) and other respiratory viruses prior to vaccine will be established at the MRC Gambia. Standard operating procedures for these will be established and internal QC/QA procedures established.

151 IFITM3 gene sequencing

Genotyping of IFITM3 gene from human genomic DNA will be performed at the University of Oxford, according to already established internal standard operating procedures and QA processes.

152 Exploratory endpoints

Additional analysis of exploratory endpoints will be undertaken at MRC Unit The Gambia

(including all flow cytometry assays as detailed) or at other external laboratories. The shipment of any samples beyond those outlined above will require the prior approval of the Gambia Government/MRC Joint Ethics Committee.

153 Audit

The trial may be audited on the request of the donor, sponsor or the Medicines Control Agency.

154 Ethical considerations

This study is conducted in accordance with the principles set out in the ICH Harmonized Tripartite Guideline for Good Clinical Practice and the Declaration of Helsinki in its current version, whichever affords the greater protection to the participants.

155 Evaluation of risks and benefits

156 Risks

Like any vaccine, immunization with SIIL LAIV (Nasovac-S) may cause side effects in some children. The side effects of Nasovac-S may include reactions for the initial days following vaccination, such as cough, fever, headache, nasal congestion, and sore throat. Most reactions are mild. Allergic reactions are also possible with any vaccination, however, they are rare. Importantly, LAIVs (both US-derived and Russian-derived LAIVs) are used routinely each year in many countries worldwide and have been found to have an acceptable safety profile.

A physician will be on-site during immunizations and will be available and equipped for any immediate and/or emergency care that may be needed. Immediate care will be provided at the health centre through which recruitment is taking place. If ongoing care is needed under such circumstances this will be provided within the Clinical Services Department at MRC Fajara.

Although a primary objective of the study is to investigate any temporary change *S. pneumoniae* or *S. aureus* burden in the nasopharynx following LAIV, it is not anticipated that any such effect would have adverse health consequences to the children receiving LAIV themselves. The wealth of post-licensure safety data for both the US and Russian LAIVs have not shown any increased bacterial pneumonia following immunization. The importance of exploring this interaction would be a potential effect on onward transmission of *S. pneumoniae* at a community level.

The vaccine to be used in the trial (Nasovac-S) is licensed for use in India and has received a WHO pre-qualification certificate for use in children ≥2 years for the prevention of influenza infections. As such, its safety profile is well established.

Azithromycin is also a licensed and widely used antibiotic, with a well-established safety profile, especially in the context of single dose treatment regimens recommended by the WHO for mass trachoma treatment campaigns.

The expected AE associated with Nasovac-S and azithromycin are defined in the SmPC for the products (see Section 4 and appendix).

Blood sampling will cause some pain at the time and generally results in the development of a bruise but has no other risks. The volumes of blood to be taken, even in children, are not of physiological significance. Obtaining an NPS is briefly irritating but is not associated with

significant risk otherwise. Obtaining an oral swab is usually well tolerated and not associated with any significant risks.

157 Benefits

Children in The Gambia are not routinely offered LAIV despite WHO recommendations that as a high risk group, children aged 2 – 5 years old should be considered for vaccination. By participating in this study, children will receive active influenza vaccine and may therefore be protected from seasonal influenza viruses circulating during the year of vaccination. However, given the contrasting efficacy data to date (section 1.1.3.3), we cannot guarantee a direct benefit to all participants in this trial. Regardless of vaccination received in the study, all children may benefit from enhanced medical observation during the course of the study.

N = 35 children in the study will receive a single dose of azithromycin, a widely used antibiotic to treat infections such as trachoma, otitis media and pneumonia. The children in this study, however, will be healthy and not require antibiotic treatment for their own health.

No other formal incentives/monetary benefits will be offered to participant in the trial although transport costs and the cost of credit for mobile phones will be remunerated. During their participation and until the 31st of October of the year of recruitment, children will have access to health care, which is consistent with the care provided through government health centres, but in this case offered through the clinical trial sites. Such access is important to ensuring all AE are collected and their follow-up is completed prior to transition back the care by the governmental health centres.

By participating in this study, the child and consenting parent/guardian will also contribute greatly needed information regarding the immunogenicity of mucosal live attenuated influenza vaccines and specifically with relevance to populations in sub-Saharan Africa. These data will inform the influenza vaccine field how best to optimize mucosal live attenuated influenza vaccines in the future. Development of new influenza vaccines that are easier and faster to produce, more immunogenic and potentially less expensive, and more appropriate for developing world populations would be a great contribution to global public health. A participant or their parent/guardian may feel benefit from knowing they have contributed to public health in this way.

158 Rationale for participant selection

The inclusion and exclusion criteria for the trial are outlined in section 5.3. They aim to be broadly inclusive while restricting the recruitment of children who may suffer from adverse effects following vaccination with Nasovac-S. The age group included is also consistent with those recommended for vaccination by the WHO as they are identified as a group at high risk from influenza infections. While this adds to the potential benefits of the study, it also means that study findings will be more widely relevant and generalizable.

159 Definition of legal guardian in the absence of a parent

There is rarely a formal legal process in The Gambia by which parental responsibility is transferred to another adult in the absence of a parent. Under these circumstances, a single adult who is identified by the family as being primarily responsible for an infant's care, will be defined. If the parents of an infant are not alive, such a guardian will be asked to sign a 'guardian statement'. They will then be considered to be solely responsible for all decisions made regarding the trial on behalf of the infant. If such an individual is not identified or is not willing to sign the statement, the infant will not be recruited.

Consent will only be obtained from a guardian when neither the infant's father nor their

mother is alive. Consent will not be accepted from a guardian if the mother or father are not present of other reasons (e.g. as a result of travel) unless guardianship has been legally handed over to another person and documentation is available to confirm this.

160 Informed consent

The process for obtaining informed consent and the controls in place to ensure consent is given voluntarily and without coercion are outlined in detail in section 6.1.2. Given the low rate of English literacy, the process of obtaining informed consent is of exceptional importance and will be a focus for trial monitoring activities. No remuneration for enrolling in the trial will be provided. Transport fares will be provided. All ICD in use will be approved by the Gambia Government/MRC Joint Ethics Committee. Should any new information which is considered to have potential implications for informed consent become available during the trial this will be made available to study participant and informed consent will be repeated using a further approved ICD. The provision of informed consent is an ongoing process and can be withdrawn at any time.

161 Participant confidentiality

All participant-identifiable information (names, addresses, contact details etc) will be held securely and will not be available to anybody other than those in the investigator team, external monitors or auditors working on behalf of the sponsor and, on request, rightful individuals from the Gambia Government/MRC Joint Ethics Committee and the National Regulatory Authority (The Medicine's Board). Information with this regard will be included as part of the informed consent process. Paper records including identifiable source documents will be held securely in access restricted filing cabinets. Electronic record will be on access restricted servers in line with the applicable data security and data access policies.

162 Future use of stored specimens

Additional consent will be obtained for the storage and future use of any specimens remaining following the assessment of the specified endpoints for research of relevance to the people of The Gambia and related to vaccination, immunity and infection. Such additional consent is not required for enrolment in the main trial. The future use of any stored specimens would require the approval of the Gambia Government/MRC Joint Ethics committee.

163 Financing

164 Funding

This study is funded through a Wellcome Trust Intermediate Clinical Fellowship award to the PI, Dr. Thushan de Silva

165 Clinical trial insurance

The MRC is not insured but has indemnity arrangements in place such that public funding is provided to meet claims. The MRC accepts that it might face claims for damages in cases where it sponsors the research (that is it has responsibility for securing the arrangements for initiating, managing and financing the study including any research carried out by its Units); and the MRC, or any of its employees, or any person formally acting with the MRC’s authority, have been negligent or have failed to adhere to the relevant guidelines/guidance, legislation or procedure on good practice in relation to medical research; and that negligence or failure to adhere to legislation, etc has caused or has materially contributed to the personal injury suffered by the individual making the claim.

Ex gratia payments – where the MRC is sponsor of research: In relation to instances where

the MRC is the sponsor of research the MRC may consider making an ex gratia payment when a significant adverse reaction in the form of a personal injury has occurred which is likely to have been caused by, or materially contributed to, by participation in a research study. In deciding whether to make such a payment, the MRC will not require the research participant to demonstrate that the personal injury has been caused by a breach of any duty of care that may have been owed by the MRC.

166 Publication policy

Trial results will be published in high-impact peer reviewed scientific journals as soon as they are available and will also be disseminated through presentations at scientific conferences and other academic meetings.

Results will be made available to parents/guardian of research-participants and their wider families and to the participating communities at large through their presentation at community open-days held at the clinical trial site (Sukuta).

No information on individual participants will be presented either in publications or other presentations and individual participants will not have access to their own results. This will be specified as part of the informed consent process.

167 References

1. Biesbroek G, Tsivtsivadze E, Sanders EA, Montijn R, Veenhoven RH, Keijser BJ, et al. Early respiratory microbiota composition determines bacterial succession patterns and respiratory health in children. Am J Respir Crit Care Med. 2014;190(11):1283-92. Epub 2014/10/21. doi: 10.1164/rccm.201407-1240OC. PubMed PMID: 25329446.
2. WHO weekly epidemiological record. 2012;21(87):201-16.
3. Bracco Neto H, Farhat CK, Tregnaghi MW, Madhi SA, Razmpour A, Palladino G, et al. Efficacy and safety of 1 and 2 doses of live attenuated influenza vaccine in vaccine-naive children. Pediatr Infect Dis J. 2009;28(5):365-71. Epub 2009/04/28. doi: 10.1097/INF.0b013e31819219b8
00006454-200905000-00001 [pii]. PubMed PMID: 19395948.
4. Belshe RB, Gruber WC, Mendelman PM, Cho I, Reisinger K, Block SL, et al. Efficacy of vaccination with live attenuated, cold-adapted, trivalent, intranasal influenza virus vaccine against a variant (A/Sydney) not contained in the vaccine. J Pediatr. 2000;136(2):168-75. Epub 2000/02/05. doi: S0022-3476(00)70097-7 [pii]. PubMed PMID: 10657821.
5. Belshe RB, Mendelman PM, Treanor J, King J, Gruber WC, Piedra P, et al. The efficacy of live attenuated, cold-adapted, trivalent, intranasal influenzavirus vaccine in children. N Engl J Med. 1998;338(20):1405-12. Epub 1998/05/15. doi: 10.1056/NEJM199805143382002. PubMed PMID: 9580647.
6. Vesikari T, Fleming DM, Aristegui JF, Vertruyen A, Ashkenazi S, Rappaport R, et al. Safety, efficacy, and effectiveness of cold-adapted influenza vaccine-trivalent against community-acquired, culture-confirmed influenza in young children attending day care. Pediatrics. 2006;118(6):2298-312. Epub 2006/12/05. doi: 118/6/2298 [pii]
10.1542/peds.2006-0725. PubMed PMID: 17142512.
7. Ambrose CS, Wu X, Jones T, Mallory RM. The role of nasal IgA in children vaccinated with live attenuated influenza vaccine. Vaccine. 2012;30(48):6794-801. Epub 2012/09/25. doi: S0264-410X(12)01328-X [pii]

10.1016/j.vaccine.2012.09.018. PubMed PMID: 23000125.
8. Katz MA, Schoub BD, Heraud JM, Breiman RF, Njenga MK, Widdowson MA. Influenza in Africa: uncovering the epidemiology of a long-overlooked disease. J Infect Dis. 2012;206 Suppl 1:S1-4. Epub 2012/11/28. doi: jis548 [pii]
10.1093/infdis/jis548. PubMed PMID: 23169953.
9. Radin JM, Katz MA, Tempia S, Talla Nzussouo N, Davis R, Duque J, et al. Influenza surveillance in 15 countries in Africa, 2006-2010. J Infect Dis. 2012;206 Suppl 1:S14-21. Epub 2012/11/28. doi: jis606 [pii]
10.1093/infdis/jis606. PubMed PMID: 23169960.
10. Schoub BD, Gessner BD, Ampofo W, Cohen AL, Steffen CA. Afriflu2--second international workshop on influenza vaccination in the African continent--8 November 2012, Cape Town (South Africa). Vaccine. 2013;31(35):3461-6. Epub 2013/04/23. doi: S0264-410X(13)00460-X [pii]
10.1016/j.vaccine.2013.04.021. PubMed PMID: 23602535.
11. Nair H, Brooks WA, Katz M, Roca A, Berkley JA, Madhi SA, et al. Global burden of respiratory infections due to seasonal influenza in young children: a systematic review and meta-analysis. Lancet. 2011;378(9807):1917-30. Epub 2011/11/15. doi: S0140-6736(11)61051-9 [pii]
10.1016/S0140-6736(11)61051-9. PubMed PMID: 22078723.
12. Lafond KE, Nair H, Rasooly MH, Valente F, Booy R, Rahman M, et al. Global Role and Burden of Influenza in Pediatric Respiratory Hospitalizations, 1982-2012: A Systematic Analysis. PLoS medicine. 2016;13(3):e1001977. doi: 10.1371/journal.pmed.1001977. PubMed PMID: 27011229; PubMed Central PMCID: PMC4807087.
13. Niang MN, Dosseh A, Ndiaye K, Sagna M, Gregory V, Goudiaby D, et al. Sentinel surveillance for influenza in Senegal, 1996-2009. J Infect Dis. 2012;206 Suppl 1:S129-35. Epub 2012/11/28. doi: jis576 [pii]
10.1093/infdis/jis576. PubMed PMID: 23169958.
14. Niang MN, Diop OM, Sarr FD, Goudiaby D, Malou-Sompy H, Ndiaye K, et al. Viral etiology of respiratory infections in children under 5 years old living in tropical rural areas of Senegal: The EVIRA project. J Med Virol. 2010;82(5):866-72. doi: 10.1002/jmv.21665. PubMed PMID: 20336732.
15. Levine OS, O'Brien KL, Deloria-Knoll M, Murdoch DR, Feikin DR, DeLuca AN, et al. The Pneumonia Etiology Research for Child Health Project: a 21st century childhood pneumonia etiology study. Clin Infect Dis. 2012;54 Suppl 2:S93-101. Epub 2012/03/21. doi: cir1052 [pii]
10.1093/cid/cir1052. PubMed PMID: 22403238; PubMed Central PMCID: PMC3297546.
16. Chan W, Zhou H, Kemble G, Jin H. The cold adapted and temperature sensitive influenza A/Ann Arbor/6/60 virus, the master donor virus for live attenuated influenza vaccines, has multiple defects in replication at the restrictive temperature. Virology. 2008;380(2):304-11. Epub 2008/09/05. doi: S0042-6822(08)00486-8 [pii]
10.1016/j.virol.2008.07.027. PubMed PMID: 18768193.
17. Jin H, Lu B, Zhou H, Ma C, Zhao J, Yang CF, et al. Multiple amino acid residues confer temperature sensitivity to human influenza virus vaccine strains (FluMist) derived from cold-adapted A/Ann Arbor/6/60. Virology. 2003;306(1):18-24. Epub 2003/03/07. doi: S0042682202000351 [pii]. PubMed PMID: 12620793.
18. Rudenko L, van den Bosch H, Kiseleva I, Mironov A, Naikhin A, Larionova N, et al. Live attenuated pandemic influenza vaccine: clinical studies on A/17/California/2009/38 (H1N1) and licensing of the Russian-developed technology to WHO for pandemic influenza preparedness in developing countries. Vaccine. 2011;29 Suppl 1:A40-4. doi: 10.1016/j.vaccine.2011.04.122. PubMed PMID: 21684428.

19. Ambrose CS, Levin MJ, Belshe RB. The relative efficacy of trivalent live attenuated and inactivated influenza vaccines in children and adults. Influenza Other Respir Viruses. 2011;5(2):67-75. doi: 10.1111/j.1750-2659.2010.00183.x. PubMed PMID: 21306569; PubMed Central PMCID: PMC3151550.
20. Osterholm MT, Kelley NS, Sommer A, Belongia EA. Efficacy and effectiveness of influenza vaccines: a systematic review and meta-analysis. Lancet Infect Dis. 2012;12(1):36-44. doi: 10.1016/S1473-3099(11)70295-X. PubMed PMID: 22032844.
21. Rhorer J, Ambrose CS, Dickinson S, Hamilton H, Oleka NA, Malinoski FJ, et al. Efficacy of live attenuated influenza vaccine in children: A meta-analysis of nine randomized clinical trials. Vaccine. 2009;27(7):1101-10. doi: 10.1016/j.vaccine.2008.11.093. PubMed PMID: 19095024.
22. Breiman RF, Brooks WA, Goswami D, Lagos R, Borja-Tabora C, Lanata CF, et al. A multinational, randomized, placebo-controlled trial to assess the immunogenicity, safety, and tolerability of live attenuated influenza vaccine coadministered with oral poliovirus vaccine in healthy young children. Vaccine. 2009;27(40):5472-9. doi: 10.1016/j.vaccine.2009.07.002. PubMed PMID: 19616493.
23. Toback SL, Ambrose CS, Eaton A, Hansen J, Aukes L, Lewis N, et al. A postlicensure evaluation of the safety of Ann Arbor strain live attenuated influenza vaccine in children 24-59 months of age. Vaccine. 2013;31(14):1812-8. doi: 10.1016/j.vaccine.2013.01.055. PubMed PMID: 23395734.
24. Rudenko LG, Lonskaya NI, Klimov AI, Vasilieva RI, Ramirez A. Clinical and epidemiological evaluation of a live, cold-adapted influenza vaccine for 3-14-year-olds. Bulletin of the World Health Organization. 1996;74(1):77-84. PubMed PMID: 8653819; PubMed Central PMCID: PMC2486837 attenuated influenza vaccines, with most attention having been directed to the development of cold-adapted (CA), attenuated reassortant vaccines. Such vaccines are widely used in the Russian Federation to immunize children. Findings are reported from a study of live, CA reassortant mono-, di-, and trivalent influenza type A and B vaccines in a series of controlled clinical and epidemiological investigations involving almost 130,000 children aged 3-15 years. Clinical, immunological, and morbidity investigations of the vaccinated children and a control group over a six-month follow-up period indicate that the vaccines were completely attenuated by the children. Transient febrile reactions occurred in less than 1% of the children after vaccination, including double seronegative individuals with low antibody titres. The type A reisolates examined were genetically stable. Furthermore, the reassortants did not suppress each other after simultaneous inoculation of children and stimulated antibody response to influenza virus strains A1, A3, and B. The incidence of influenza-like diseases was approximately 30-40% lower among the vaccinated group than among the control group. It is noted that this study demonstrates, for the first time, the efficacy of CA vaccine against infections caused by influenza B virus.
25. Alexandrova GI, Budilovsky GN, Koval TA, Polezhaev FI, Garmashova LM, Ghendon Yu Z, et al. Study of live recombinant cold-adapted influenza bivalent vaccine of type A for use in children: an epidemiological control trial. Vaccine. 1986;4(2):114-8. PubMed PMID: 3524050.
26. Rudenko LG, Slepushkin AN, Monto AS, Kendal AP, Grigorieva EP, Burtseva EP, et al. Efficacy of live attenuated and inactivated influenza vaccines in schoolchildren and their unvaccinated contacts in Novgorod, Russia. J Infect Dis. 1993;168(4):881-7. PubMed PMID: 8376833.
27. Slepushkin AN, Obrosova-Serova NP, Burtseva EI, Rudenko LG, Govorkova EA, Vartanyan RV, et al. Comparison of live attenuated and inactivated influenza vaccines in

schoolchildren in Russia: I. Safety and efficacy in two Moscow schools, 1987/88. Vaccine. 1993;11(3):323-8. PubMed PMID: 8447161.
28. Rudenko LG, Arden NH, Grigorieva E, Naychin A, Rekstin A, Klimov AI, et al. Immunogenicity and efficacy of Russian live attenuated and US inactivated influenza vaccines used alone and in combination in nursing home residents. Vaccine. 2000;19(2-3):308-18. PubMed PMID: 10930686.
29. Kulkarni PS, Raut SK, Dhere RM. A post-marketing surveillance study of a human live-virus pandemic influenza A (H1N1) vaccine (Nasovac ((R))) in India. Hum Vaccin Immunother. 2013;9(1):122-4. doi: 10.4161/hv.22317. PubMed PMID: 23442586; PubMed Central PMCID: PMC3667925.
30. Kulkarni PS, Agarkhedkar S, Lalwani S, Bavdekar AR, Jog S, Raut SK, et al. Effectiveness of an Indian-made attenuated influenza A(H1N1)pdm 2009 vaccine: a case control study. Hum Vaccin Immunother. 2014;10(3):566-71. PubMed PMID: 24406308; PubMed Central PMCID: PMC4130283.
31. Ortiz JR, Goswami D, Lewis KD, Sharmeen AT, Ahmed M, Rahman M, et al. Safety of Russian-backbone seasonal trivalent, live-attenuated influenza vaccine in a phase II randomized placebo-controlled clinical trial among children in urban Bangladesh. Vaccine. 2015;33(29):3415-21. doi: 10.1016/j.vaccine.2015.04.048. PubMed PMID: 25917680.
32. Belshe RB, Gruber WC, Mendelman PM, Mehta HB, Mahmood K, Reisinger K, et al. Correlates of immune protection induced by live, attenuated, cold-adapted, trivalent, intranasal influenza virus vaccine. J Infect Dis. 2000;181(3):1133-7. Epub 2000/03/18. doi: JID990902 [pii]
10.1086/315323. PubMed PMID: 10720541.
33. Clements ML, Betts RF, Tierney EL, Murphy BR. Serum and nasal wash antibodies associated with resistance to experimental challenge with influenza A wild-type virus. J Clin Microbiol. 1986;24(1):157-60. Epub 1986/07/01. PubMed PMID: 3722363; PubMed Central PMCID: PMC268857.
34. Clements ML, O'Donnell S, Levine MM, Chanock RM, Murphy BR. Dose response of A/Alaska/6/77 (H3N2) cold-adapted reassortant vaccine virus in adult volunteers: role of local antibody in resistance to infection with vaccine virus. Infect Immun. 1983;40(3):1044-51. Epub 1983/06/01. PubMed PMID: 6852910; PubMed Central PMCID: PMC348156.
35. Forrest BD, Pride MW, Dunning AJ, Capeding MR, Chotpitayasunondh T, Tam JS, et al. Correlation of cellular immune responses with protection against culture-confirmed influenza virus in young children. Clin Vaccine Immunol. 2008;15(7):1042-53. Epub 2008/05/02. doi: CVI.00397-07 [pii]
10.1128/CVI.00397-07. PubMed PMID: 18448618; PubMed Central PMCID: PMC2446637.
36. Sasaki S, Jaimes MC, Holmes TH, Dekker CL, Mahmood K, Kemble GW, et al. Comparison of the influenza virus-specific effector and memory B-cell responses to immunization of children and adults with live attenuated or inactivated influenza virus vaccines. J Virol. 2007;81(1):215-28. Epub 2006/10/20. doi: JVI.01957-06 [pii]
10.1128/JVI.01957-06. PubMed PMID: 17050593; PubMed Central PMCID: PMC1797237.
37. Edwards KM, Dupont WD, Westrich MK, Plummer WD, Jr., Palmer PS, Wright PF. A randomized controlled trial of cold-adapted and inactivated vaccines for the prevention of influenza A disease. J Infect Dis. 1994;169(1):68-76. Epub 1994/01/01. PubMed PMID: 8277200.
38. Mallory RM, Malkin E, Ambrose CS, Bellamy T, Shi L, Yi T, et al. Safety and immunogenicity following administration of a live, attenuated monovalent 2009 H1N1 influenza vaccine to children and adults in two randomized controlled trials. PLoS One. 2010;5(10):e13755. Epub 2010/11/10. doi: 10.1371/journal.pone.0013755. PubMed PMID: 21060780; PubMed Central PMCID: PMC2966412.

39. He XS, Holmes TH, Zhang C, Mahmood K, Kemble GW, Lewis DB, et al. Cellular immune responses in children and adults receiving inactivated or live attenuated influenza vaccines. J Virol. 2006;80(23):11756-66. Epub 2006/09/15. doi: JVI.01460-06 [pii]
10.1128/JVI.01460-06. PubMed PMID: 16971435; PubMed Central PMCID: PMC1642596.
40. Hoft DF, Babusis E, Worku S, Spencer CT, Lottenbach K, Truscott SM, et al. Live and inactivated influenza vaccines induce similar humoral responses, but only live vaccines induce diverse T-cell responses in young children. J Infect Dis. 2011;204(6):845-53. Epub 2011/08/19. doi: jir436 [pii]
10.1093/infdis/jir436. PubMed PMID: 21846636; PubMed Central PMCID: PMC3156924.
41. Huda MN, Lewis Z, Kalanetra KM, Rashid M, Ahmad SM, Raqib R, et al. Stool microbiota and vaccine responses of infants. Pediatrics. 2014;134(2):e362-72. Epub 2014/07/09. doi: peds.2013-3937 [pii]
10.1542/peds.2013-3937. PubMed PMID: 25002669; PubMed Central PMCID: PMC4187229.
42. Praharaj I, John SM, Bandyopadhyay R, Kang G. Probiotics, antibiotics and the immune responses to vaccines. Philos Trans R Soc Lond B Biol Sci. 2015;370(1671). Epub 2015/05/13. doi: rstb.2014.0144 [pii]
10.1098/rstb.2014.0144. PubMed PMID: 25964456.
43. Chinen T, Rudensky AY. The effects of commensal microbiota on immune cell subsets and inflammatory responses. Immunol Rev. 2012;245(1):45-55. Epub 2011/12/16. doi: 10.1111/j.1600-065X.2011.01083.x. PubMed PMID: 22168413.
44. Goto Y, Panea C, Nakato G, Cebula A, Lee C, Diez MG, et al. Segmented filamentous bacteria antigens presented by intestinal dendritic cells drive mucosal Th17 cell differentiation. Immunity. 2014;40(4):594-607. Epub 2014/04/02. doi: S1074-7613(14)00085-5 [pii]
10.1016/j.immuni.2014.03.005. PubMed PMID: 24684957; PubMed Central PMCID: PMC4084624.
45. Hapfelmeier S, Lawson MA, Slack E, Kirundi JK, Stoel M, Heikenwalder M, et al. Reversible microbial colonization of germ-free mice reveals the dynamics of IgA immune responses. Science. 2010;328(5986):1705-9. Epub 2010/06/26. doi: 328/5986/1705 [pii]
10.1126/science.1188454. PubMed PMID: 20576892; PubMed Central PMCID: PMC3923373.
46. Oh JZ, Ravindran R, Chassaing B, Carvalho FA, Maddur MS, Bower M, et al. TLR5-Mediated Sensing of Gut Microbiota Is Necessary for Antibody Responses to Seasonal Influenza Vaccination. Immunity. 2014;41(3):478-92. Epub 2014/09/16. doi: S1074-7613(14)00303-3 [pii]
10.1016/j.immuni.2014.08.009. PubMed PMID: 25220212; PubMed Central PMCID: PMC4169736.
47. Abt MC, Osborne LC, Monticelli LA, Doering TA, Alenghat T, Sonnenberg GF, et al. Commensal bacteria calibrate the activation threshold of innate antiviral immunity. Immunity. 2012;37(1):158-70. Epub 2012/06/19. doi: S1074-7613(12)00237-3 [pii]
10.1016/j.immuni.2012.04.011. PubMed PMID: 22705104; PubMed Central PMCID: PMC3679670.
48. Ichinohe T, Lee HK, Ogura Y, Flavell R, Iwasaki A. Inflammasome recognition of influenza virus is essential for adaptive immune responses. J Exp Med. 2009;206(1):79-87. Epub 2009/01/14. doi: jem.20081667 [pii]
10.1084/jem.20081667. PubMed PMID: 19139171; PubMed Central PMCID: PMC2626661.
49. Ichinohe T, Pang IK, Kumamoto Y, Peaper DR, Ho JH, Murray TS, et al. Microbiota regulates immune defense against respiratory tract influenza A virus infection. Proc Natl Acad Sci U S A. 2011;108(13):5354-9. Epub 2011/03/16. doi: 1019378108 [pii]

10.1073/pnas.1019378108. PubMed PMID: 21402903; PubMed Central PMCID: PMC3069176.
50. Blaser MJ, Falkow S. What are the consequences of the disappearing human microbiota? Nat Rev Microbiol. 2009;7(12):887-94. Epub 2009/11/10. doi: nrmicro2245 [pii] 10.1038/nrmicro2245. PubMed PMID: 19898491.
51. Bogaert D, De Groot R, Hermans PW. Streptococcus pneumoniae colonisation: the key to pneumococcal disease. Lancet Infect Dis. 2004;4(3):144-54. Epub 2004/03/05. doi: 10.1016/S1473-3099(04)00938-7
S1473309904009387 [pii]. PubMed PMID: 14998500.
52. Garcia-Rodriguez JA, Fresnadillo Martinez MJ. Dynamics of nasopharyngeal colonization by potential respiratory pathogens. J Antimicrob Chemother. 2002;50 Suppl S2:59-73. Epub 2003/01/31. PubMed PMID: 12556435.
53. McCullers JA. Insights into the interaction between influenza virus and pneumococcus. Clin Microbiol Rev. 2006;19(3):571-82. Epub 2006/07/19. doi: 19/3/571 [pii] 10.1128/CMR.00058-05. PubMed PMID: 16847087; PubMed Central PMCID: PMC1539103.
54. McCullers JA, Rehg JE. Lethal synergism between influenza virus and Streptococcus pneumoniae: characterization of a mouse model and the role of platelet-activating factor receptor. J Infect Dis. 2002;186(3):341-50. Epub 2002/07/23. doi: JID020206 [pii] 10.1086/341462. PubMed PMID: 12134230.
55. Nakamura S, Davis KM, Weiser JN. Synergistic stimulation of type I interferons during influenza virus coinfection promotes Streptococcus pneumoniae colonization in mice. J Clin Invest. 2011;121(9):3657-65. Epub 2011/08/16. doi: 57762 [pii]
10.1172/JCI57762. PubMed PMID: 21841308; PubMed Central PMCID: PMC3163966.
56. Plotkowski MC, Puchelle E, Beck G, Jacquot J, Hannoun C. Adherence of type I Streptococcus pneumoniae to tracheal epithelium of mice infected with influenza A/PR8 virus. Am Rev Respir Dis. 1986;134(5):1040-4. Epub 1986/11/01. PubMed PMID: 3777666.
57. Sweet C, Bird RA, Husseini RH, Smith H. Differential replication of attenuated and virulent influenza viruses in organ cultures of ferret bronchial epithelium. Brief report. Arch Virol. 1984;80(2-3):219-24. Epub 1984/01/01. PubMed PMID: 6721679.
58. Avadhanula V, Rodriguez CA, Devincenzo JP, Wang Y, Webby RJ, Ulett GC, et al. Respiratory viruses augment the adhesion of bacterial pathogens to respiratory epithelium in a viral species- and cell type-dependent manner. J Virol. 2006;80(4):1629-36. Epub 2006/01/28. doi: 80/4/1629 [pii]
10.1128/JVI.80.4.1629-1636.2006. PubMed PMID: 16439519; PubMed Central PMCID: PMC1367158.
59. Didierlaurent A, Goulding J, Patel S, Snelgrove R, Low L, Bebien M, et al. Sustained desensitization to bacterial Toll-like receptor ligands after resolution of respiratory influenza infection. J Exp Med. 2008;205(2):323-9. Epub 2008/01/30. doi: jem.20070891 [pii]
10.1084/jem.20070891. PubMed PMID: 18227219; PubMed Central PMCID: PMC2271005.
60. Sun K, Metzger DW. Inhibition of pulmonary antibacterial defense by interferon-gamma during recovery from influenza infection. Nat Med. 2008;14(5):558-64. Epub 2008/04/29. doi: nm1765 [pii]
10.1038/nm1765. PubMed PMID: 18438414.
61. van der Sluijs KF, van Elden LJ, Nijhuis M, Schuurman R, Pater JM, Florquin S, et al. IL-10 is an important mediator of the enhanced susceptibility to pneumococcal pneumonia after influenza infection. J Immunol. 2004;172(12):7603-9. Epub 2004/06/10. doi: 172/12/7603 [pii]. PubMed PMID: 15187140.

62. Mina MJ, McCullers JA, Klugman KP. Live attenuated influenza vaccine enhances colonization of Streptococcus pneumoniae and Staphylococcus aureus in mice. MBio. 2014;5(1). Epub 2014/02/20. doi: mBio.01040-13 [pii]
10.1128/mBio.01040-13. PubMed PMID: 24549845; PubMed Central PMCID: PMC3944816.
63. Thors V, Christensen H, Morales-Aza B, Vipond I, Muir P, Finn A. The Effects of Live Attenuated Influenza Vaccine on Nasopharyngeal Bacteria in Healthy 2 to 4 Year Olds. A Randomized Controlled Trial. Am J Respir Crit Care Med. 2016;193(12):1401-9. doi: 10.1164/rccm.201510-2000OC. PubMed PMID: 26742001; PubMed Central PMCID: PMC4910891.
64. Kwambana BA, Barer MR, Bottomley C, Adegbola RA, Antonio M. Early acquisition and high nasopharyngeal co-colonisation by Streptococcus pneumoniae and three respiratory pathogens amongst Gambian new-borns and infants. BMC Infect Dis. 2011;11:175. Epub 2011/06/22. doi: 1471-2334-11-175 [pii]
10.1186/1471-2334-11-175. PubMed PMID: 21689403; PubMed Central PMCID: PMC3129300.
65. Belshe RB, Edwards KM, Vesikari T, Black SV, Walker RE, Hultquist M, et al. Live attenuated versus inactivated influenza vaccine in infants and young children. N Engl J Med. 2007;356(7):685-96. Epub 2007/02/16. doi: 356/7/685 [pii]
10.1056/NEJMoa065368. PubMed PMID: 17301299.
66. Nakaya HI, Wrammert J, Lee EK, Racioppi L, Marie-Kunze S, Haining WN, et al. Systems biology of vaccination for seasonal influenza in humans. Nat Immunol. 2011;12(8):786-95. Epub 2011/07/12. doi: ni.2067 [pii]
10.1038/ni.2067. PubMed PMID: 21743478; PubMed Central PMCID: PMC3140559.
67. Querec TD, Akondy RS, Lee EK, Cao W, Nakaya HI, Teuwen D, et al. Systems biology approach predicts immunogenicity of the yellow fever vaccine in humans. Nat Immunol. 2009;10(1):116-25. Epub 2008/11/26. doi: ni.1688 [pii]
10.1038/ni.1688. PubMed PMID: 19029902; PubMed Central PMCID: PMC4049462.
68. Ayele B, Gebre T, House JI, Zhou Z, McCulloch CE, Porco TC, et al. Adverse events after mass azithromycin treatments for trachoma in Ethiopia. The American journal of tropical medicine and hygiene. 2011;85(2):291-4. doi: 10.4269/ajtmh.2011.11-0056. PubMed PMID: 21813850; PubMed Central PMCID: PMC3144828.
69. Biesbroek G, Sanders EA, Roeselers G, Wang X, Caspers MP, Trzcinski K, et al. Deep sequencing analyses of low density microbial communities: working at the boundary of accurate microbiota detection. PLoS One. 2012;7(3):e32942. Epub 2012/03/14. doi: 10.1371/journal.pone.0032942
PONE-D-11-19618 [pii]. PubMed PMID: 22412957; PubMed Central PMCID: PMC3295791.
70. Koopmans M, de Bruin E, Godeke GJ, Friesema I, van Gageldonk R, Schipper M, et al. Profiling of humoral immune responses to influenza viruses by using protein microarray. Clinical microbiology and infection : the official publication of the European Society of Clinical Microbiology and Infectious Diseases. 2012;18(8):797-807. doi: 10.1111/j.1469-0691.2011.03701.x. PubMed PMID: 22212116.
71. Li S, Rouphael N, Duraisingham S, Romero-Steiner S, Presnell S, Davis C, et al. Molecular signatures of antibody responses derived from a systems biology study of five human vaccines. Nat Immunol. 2014;15(2):195-204. Epub 2013/12/18. doi: ni.2789 [pii]
10.1038/ni.2789. PubMed PMID: 24336226; PubMed Central PMCID: PMC3946932.
72. Warnes GR. Sample Size Estimation for Microarray Experiments. Department of Biostatistics and Computational Biology, University of Rochester, 2006.

73. Mattiello F, Verbist B, Faust K, Raes J, Shannon WD, Bijnens L, et al. A web application for sample size and power calculation in case-control microbiome studies. Bioinformatics. 2016;32(13):2038-40. doi: 10.1093/bioinformatics/btw099. PubMed PMID: 27153704.
74. Mina MJ. Reply to 'No Clinical Association of Live Attenuated Influenza Vaccine with Nasal Carriage of Bacteria or Acute Otitis Media': Specific Recommendations for Future Studies. MBio. 2014;5(3):e01173-14.
75. Short KR, Reading PC, Wang N, Diavatopoulos DA, Wijburg OL. Increased nasopharyngeal bacterial titers and local inflammation facilitate transmission of Streptococcus pneumoniae. MBio. 2012;3(5). doi: 10.1128/mBio.00255-12. PubMed PMID: 23015738; PubMed Central PMCID: PMC3518912.
76. Wang Q, Garrity GM, Tiedje JM, Cole JR. Naive Bayesian classifier for rapid assignment of rRNA sequences into the new bacterial taxonomy. Appl Environ Microbiol. 2007;73(16):5261-7. Epub 2007/06/26. doi: AEM.00062-07 [pii]
10.1128/AEM.00062-07. PubMed PMID: 17586664; PubMed Central PMCID: PMC1950982.
77. Biesbroek G, Wang X, Keijser BJ, Eijkemans RM, Trzcinski K, Rots NY, et al. Seven-valent pneumococcal conjugate vaccine and nasopharyngeal microbiota in healthy children. Emerg Infect Dis. 2014;20(2):201-10. Epub 2014/01/23. doi: 10.3201/eid2002.131220. PubMed PMID: 24447437; PubMed Central PMCID: PMC3901477.
78. de Steenhuijsen Piters WA, Heinonen S, Hasrat R, Bunsow E, Smith B, Suarez-Arrabal MC, et al. Nasopharyngeal Microbiota, Host Transcriptome and Disease Severity in Children with Respiratory Syncytial Virus Infection. Am J Respir Crit Care Med. 2016. doi: 10.1164/rccm.201602-0220OC. PubMed PMID: 27135599.
79. Tsang JS, Schwartzberg PL, Kotliarov Y, Biancotto A, Xie Z, Germain RN, et al. Global analyses of human immune variation reveal baseline predictors of postvaccination responses. Cell. 2014;157(2):499-513. doi: 10.1016/j.cell.2014.03.031. PubMed PMID: 24725414; PubMed Central PMCID: PMCPMC4139290.

168 Appendix

Times and events for groups in the study

		Informed consent			Eligibility assessment						Clinical samples					Clinical samples			Clinical samples			Clinical samples						
	Sensitization	Provision of information	Confirmation of understanding	Documentation of informed consent	History	Examination	Temperature, heart and respiratory rate measurement	Height and weight measurement	Inclusion and exclusion criteria checklist	Randomization and subject number allocation	Blood sample	Oral swab	Nasopharyngeal swab	Vaccination	Assessment of AE (direct enquiry)	Blood sample	Nasopharyngeal swab	Assessment of AE (direct enquiry)	Blood sample	Nasopharyngeal swab	Assessment of AE (direct enquiry)	Blood sample	Oral swab	Nasopharyngeal swab	Vaccination	Assessment of AE (direct enquiry)	Assessment for ILI	Nasopharyngeal swab
	Day 0 visit															Day 2 visit			Day 7 visit			Day 21 visit					On presentation*	
Pre-recruitment	X	X																										
Pre-randomization (all subjects)		X	X	X	X	X	X	X	X	X																		
Group A											X	X	X	X	X	X	X	X		X	X	X	X	X		X	X	X
Group B											X	X	X	X	X		X	X	X	X	X	X	X	X		X	X	X
Group C													X							X				X	X			

		Informed consent			Eligibility assessment									Clinical samples					Clinical samples					Clinical samples					
	Sensitization	Provision of information	Confirmation of understanding	Documentation of informed consent	History	Examination	Temperature, heart and respiratory rate measurement	Height and weight measurement	Inclusion and exclusion criteria checklist	Randomization and subject number allocation	Nasopharyngeal swab	Oral azithromycin	Assessment of AE (direct enquiry)	Blood sample	Oral swab	Nasophreyngeal swab	Vaccination	Assessment of AE (direct enquiry)	Blood sample	Nasophreyngeal swab	Assessment of AE (direct enquiry)	Nasophreyngeal swab	Assessment of AE (direct enquiry)	Blood sample	Oral swab	Nasophreyngeal swab	Assessment of AE (direct enquiry)	Assessment for ILI	Nasophreyngeal swab
	Day -28 visit													Day 0 visit					Day 2 visit			Day 7 visit		Day 21 visit				On presentation*	
Pre-recruitment	X	X																											
Pre-randomization		X	X	X	X	X	X	X	X	X																			
Group D											X	X	X	X	X	X	X	X	X	X	X	X	X	X	X	X	X	X	X

* Subjects presenting with possible influenza-like illness, following completion of day 21 post-LAIV visit, up to and including the 31st of October of the year of recruitment.
